# Priming Cells with Modulators of Cellular Signaling Ensures Enhanced Selectivity of HSPG-Specific Protein–Drug Conjugate Delivery

**DOI:** 10.1021/jacs.6c08409

**Published:** 2026-08-28

**Authors:** Aleksandra Chorążewska, Krzysztof Ciura, Magdalena Milczarek, Dagmara Kłopotowska, Adam Pomorski, Anna Więch-Walów, Błażej Łukianowski, Paweł Gajdzis, Artur Krężel, Rafał Bartoszewski, Joanna Wietrzyk, Liliana Schaefer, Natalia Porębska, Łukasz Opaliński

**Affiliations:** † Department of Medical Biotechnology, Faculty of Biotechnology, 220033 University of Wroclaw, Joliot-Curie 14a, Wroclaw 50-383, Poland; ‡ Department of Experimental Oncology, 89217 Hirszfeld Institute of Immunology and Experimental Therapy, Weigla 12, Wroclaw 53-114, Poland; § Department of Chemical Biology, Faculty of Biotechnology, 49572 University of Wroclaw, Joliot-Curie 14a, Wroclaw 50-383, Poland; ∥ Department of Biophysics, Faculty of Biotechnology, University of Wroclaw, Joliot-Curie 14a, Wroclaw 50-383, Poland; ⊥ Department of Clinical and Experimental Pathology, Wroclaw Medical University, T. Marcinkowskiego 1, Wroclaw 50-368, Poland; # Department of Pathomorphology, Fourth Military Hospital, Weigla 5, Wroclaw 53-114, Poland; ∇ Institute of Pharmacology and Toxicology, Goethe University, Theodor-Stern Kai 7, Frankfurt 605090, Germany

## Abstract

Protein–drug conjugates (PDCs) constitute a rapidly growing group of precise anticancer agents. Receptor-mediated endocytosis is a critical step in PDC action, which ensures the delivery of PDCs into the interior of cancer cells. Here, we report TriF_HS_-MMAE, the first multivalent PDC specifically targeting heparan sulfate proteoglycans (HSPGs) overexpressed by pancreatic cancer cells, which induces ultrafast and highly efficient aggregation-dependent endocytosis (ADE) of HSPGs. Using high-content screening with a near-kinome-wide library of inhibitors, we identified signaling pathways that govern ADE of HSPGs and discovered cascades that selectively operate in pancreatic cancer cells versus healthy cells. We show that priming cells with identified endocytic chemical modulators improves the targeting of the TriF_HS_-MMAE conjugate *in vitro* and *in vivo*. Overall, these findings provide insights into the interplay among signaling, endocytosis, and PDCs and support the development of specific therapies for pancreatic cancer.

## Graphical abstract

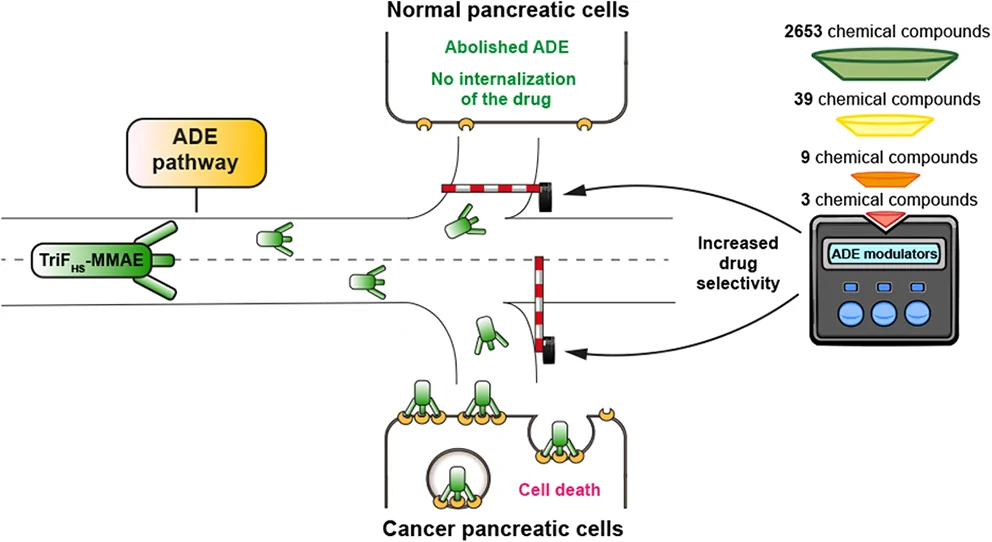

## Introduction

Protein-drug conjugates (PDCs), especially in the form of antibody-drug conjugates (ADCs), are rapidly developing as precise anticancer modalities. The foremost advantage of ADCs, their high selectivity, is determined by the interplay between the characteristics of the cancer-specific antigen and the molecular architecture of the targeting agent within the ADC, ensuring efficient delivery of the cytotoxic payload to the interior of cancer cells by endocytosis. Endocytic dynamics of individual cell-surface proteins may differ considerably and also depend on the cellular context.^1^ According to the current selection criteria, antigens designated for the ADC approach should preferably display a high expression level on the surface of cancer cells and minimal expression in healthy tissues, and should internalize to support intracellular drug delivery.^2^ Such static parameters might not necessarily reflect the capability of individual cell-surface proteins to selectively deliver an ADC into the cell interior, thereby excluding numerous potentially attractive targets from the ADC approach. On the other hand, the efficiency of endocytosis and the use of particular endocytic mechanisms might differ between normal and cancer cells.^3^ A cell-surface protein might be endocytosed much more efficiently by cancer cells than by healthy cells, supporting ADC delivery and ensuring treatment selectivity even with virtually no differences in protein expression.^4^ Modulation of the endocytosis-ADC interconnection could therefore allow targeting new cell-surface proteins and the development of novel ADCs for hitherto incurable cancers.^5^ Recent reports indicate that clustering of cell-surface receptors with extracellular multivalent ligands triggers a novel, highly efficient internalization pathway, termed aggregation-dependent endocytosis (ADE).^6–9^ The exact mechanisms and regulation of ADE are unknown; yet it is clathrin-independent and Rac1- and VAV-dependent.^6^ Since monoclonal antibodies (mAbs), the targeting agents in current ADCs, are bivalent proteins, they are incapable of inducing receptor clustering and ADE and thus have to passively rely on the endocytic activity of the cognate receptor.^5^ New multivalent architectures of targeting agents and their application to novel cancer-relevant target antigens are required to enable investigation of the therapeutic potential of ADE in selective drug delivery.^10^

Heparan sulfate proteoglycans (HSPGs) are cell-surface and secreted glycoproteins bearing linear heparan sulfate (HS) chains that play pivotal roles in cell physiology.^11^ Due to their HS, HSPGs are characterized by a highly negative charge, which facilitates interactions with several extracellular proteins, including growth factors (e.g., fibroblast growth factors (FGFs), heparin-binding EGF-like growth factor (HB-EGF), vascular endothelial growth factor (VEGF)), cytokines, and chemokines.^12^ Importantly, numerous studies have established HSPGs as key players that promote the aggressiveness of highly lethal pancreatic cancer.^13^ Glypican-1 (GPC1) is expressed at very low levels in the normal, noncancerous pancreas, whereas it is strongly overexpressed by pancreatic cancer cells and cancer-associated fibroblasts (CAFs), and has therefore been considered a pancreatic tumor marker for more than 20 years.^14^ Glypican-4 (GPC4) is significantly upregulated in pancreatic tumors compared with normal tissues, promoting chemoresistance and cancer stem-like properties, and its expression levels are associated with patient survival.^15,16^ The overexpression of HSPGs by pancreatic cancer cells, and their important role in the development and aggressiveness of pancreatic cancer, make these proteins attractive targets for the development of precision medicine modalities.^17^ HSPGs are considered to be cell-surface endocytosis receptors, and diverse HSPG-specific targeting molecules have been developed that exploit HSPG endocytosis (e.g., cell-penetrating peptides, peptide-nucleic acid complexes, exosomes, or liposomes).^18–22^ Importantly, there is no HSPG-specific ADC approved for pancreatic cancer treatment, highlighting the urgent need for the development of novel targeted therapeutics.

Here, we explored the possibility of simultaneously manipulating endocytosis both externally and internally to enhance the selectivity of HSPG targeting in pancreatic cancer cells. To follow this idea, we developed a novel multivalent, intrinsically fluorescent PDC, the TriF_HS_-MMAE, which is highly selective for the HS of HSPGs and capable of triggering ADE. We uncovered, in an unbiased manner, the signaling pathways governing ADE in pancreatic cells. Using the acquired knowledge of HSPG endocytosis/signaling interplay, we were able to prevent TriF_HS_-MMAE uptake by healthy cells while simultaneously priming cancer cells to more efficiently internalize the conjugate. By rational modulation of TriF_HS_-MMAE-selective endocytosis, we were also able to prevent TriF_HS_-MMAE toxicity to normal cells expressing HSPGs *in vivo*. Altogether, our data on PDC/endocytosis/signaling interconnection pave the way for developing innovative, precise anticancer modalities that may enable targeting of so-far “undruggable” cell surface proteins.

## Results

### Engineering of Multivalent Drug Carriers Specific for HSPGs

The aim of this study was to develop the first oligomeric HS-targeting macromolecule, capable of highly specific, tight, and multivalent binding to HSPGs that could serve as a drug carrier in a PDC approach (Figure 1a). The wild-type FGF1 binds with high affinity to both FGFRs and HSPGs, forming the ternary signaling complex at the plasma membrane.^23^ In order to develop a universal HSPG-targeting agent, we employed the natural ability of FGF1 to recognize HS and eliminated its affinity for FGFRs (Figure 1a). We constructed an FGF1_HS_ variant (Supplementary Figure 1), and with biolayer interferometry (BLI) and cellular signaling assays, we demonstrated that FGF1_HS_ is uncoupled from FGFR binding (Figure 1b) and activation (Figure 1c).

**Figure 1:**
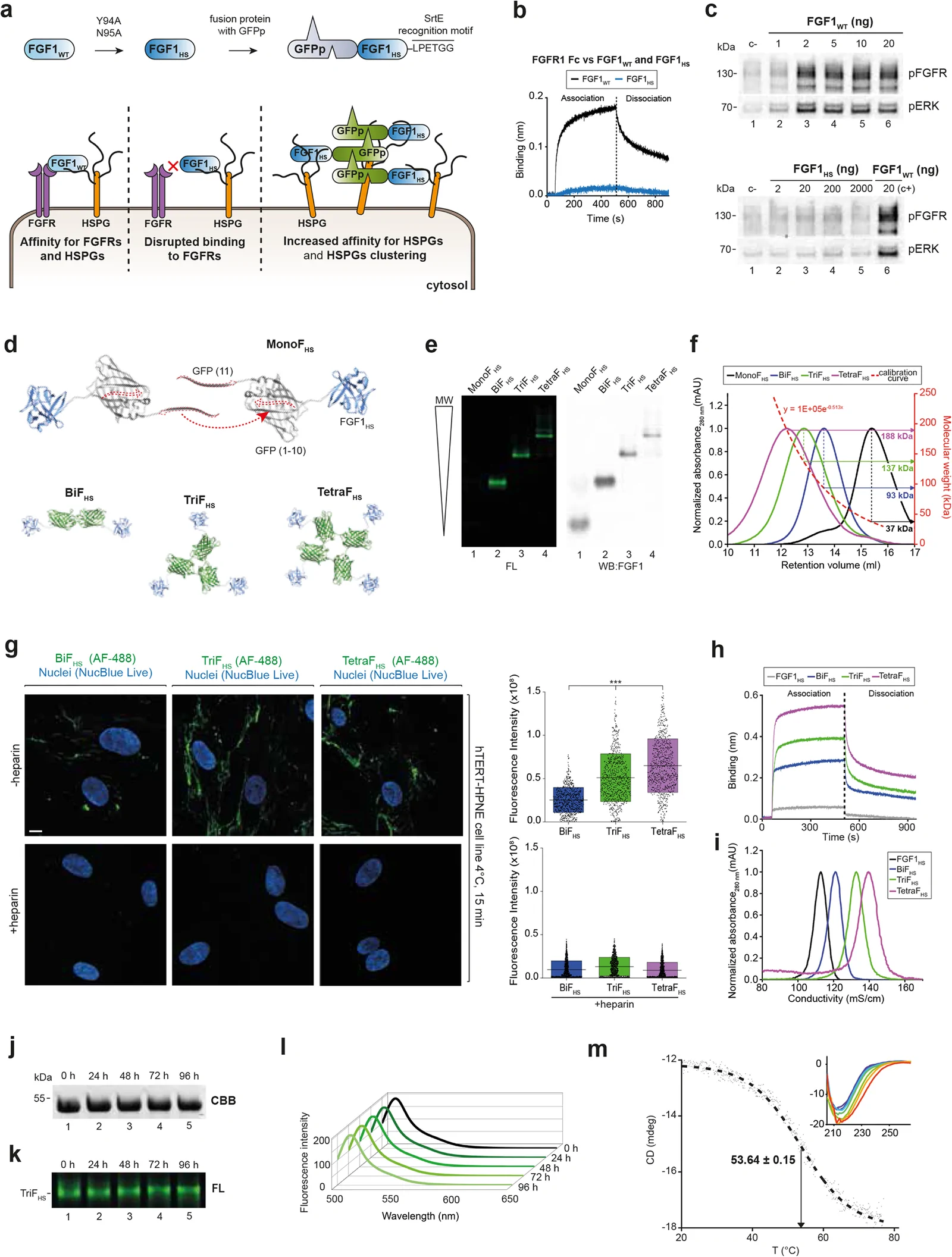
TriF_HS_ is an intrinsically fluorescent multivalent ligand selectively targeting HSPGs. a. Schematic representation of the engineered GFPp-FGF1_HS_ fusion protein and its molecular interactions with physiological binding partners of wild-type FGF1 (FGF1_WT_). **b.** Analysis of FGF1_WT_ (black) and FGF1_HS_ (blue) binding to FGFR1 using biolayer interferometry (BLI). **c.** FGFR1 phosphorylation and downstream signaling activation induced by increasing concentrations of FGF1_WT_ (top panel) or FGF1_HS_ (bottom panel) were assessed by immunoblotting. **d.** Schematic illustration of the oligomerization of GFPp-FGF1_HS_ into multivalent forms. **e.** Native PAGE and Western blot analysis of isolated GFPp-FGF1_HS_ forms (MonoF_HS_, BiF_HS_, TriF_HS_, and TetraF_HS_). **f.** Size-exclusion chromatography (SEC) profiling of isolated GFPp-FGF1_HS_ oligomers. **g.** Cell-surface binding of BiF_HS_, TriF_HS_, and TetraF_HS_ to normal pancreatic epithelial cells (hTERT-HPNE) in the presence of soluble heparin. Each point on the plot represents the fluorescence intensity of a single cell; at least 547 cells were analyzed per condition (*n* = 3). Statistical significance was determined using one-way ANOVA with Tukey’s HSD post hoc test (*p < 0.05; **p < 0.005; ***p < 0.001). **h.** Binding of distinct GFPp-FGF1_HS_ oligomeric states to GPC4, a representative heparan sulfate proteoglycan. **i.** Heparin affinity chromatography (FPLC) elution profiles of FGF1_HS_, BiF_HS_, TriF_HS_, and TetraF_HS_. **j**–**k**. Stability assessment of TriF_HS_ in 100-fold-diluted human serum under denaturing (**j**) and native (**k**) conditions. **l.** Intrinsic fluorescence spectra of TriF_HS_ incubated for up to 96 h in diluted human serum. **m.** Thermal stability of TriF_HS_ assessed by circular dichroism spectroscopy, with determination of the apparent melting temperature (*T*_m_). Statistical analyses were performed for at least three independent experiments.

To enhance the affinity of FGF1_HS_ for HS, we increased its valency in a controllable manner (Figure 1d). To this end, we genetically fused FGF1_HS_ with GFP polygons (GFPp), intrinsically fluorescent scaffolds that enable controllable oligomerization of proteins.^24^ GFPp are GFP variants in which the last β-strand is transplanted to the N-terminus of the β-barrel, facilitating the intermolecular assembly of fluorescent GFPp oligomers.^24^ This strategy resulted in the formation of bivalent, trivalent, and tetravalent FGF1_HS_, named BiF_HS_, TriF_HS_, and TetraF_HS_ respectively (Figure 1d). Next, we incorporated the LPETGG sequence at the C-terminus of GFPp-FGF1_HS_ to allow for the site-specific attachment of the cytotoxic payload via sortase A-mediated ligation (Figure 1a). We produced the GFPp-FGF1_HS_ fusion in *E. coli* (Supplementary Figure 2) and, using native PAGE, we confirmed the assembly of its distinct oligomeric forms (Supplementary Figure 3). Using HS-affinity chromatography, we isolated the particular GFPp-FGF1_HS_ oligomeric forms (Supplementary Figure 4), yielding highly pure nonfluorescent MonoF_HS_ and intrinsically fluorescent BiF_HS_, TriF_HS_, and TetraF_HS_ (Figures 1e, Supplementary Figures 5 and 6) and confirmed their oligomeric state with calibrated gel filtration (Figure 1f) and dynamic light scattering (DLS) (Supplementary Figure 7). Using the intrinsic fluorescence of the developed proteins and quantitative confocal microscopy, we demonstrated that the tested variants specifically bind to HS on the surface of model healthy pancreatic hTERT-HPNE cells (Figure 1g). Notably, an excess of soluble HS fully outcompeted the interaction of BiF_HS_, TriF_HS_, and TetraF_HS_ with the cells (Figure 1g). With BLI, we also confirmed a direct interaction of MonoF_HS_, BiF_HS_, TriF_HS_, and TetraF_HS_ with a model HSPG, GPC4, overproduced by pancreatic cancer cells (Figure 1h).^15,16^ A stretch of lysine (L112, L113, L118, L128) and arginine (R119, R122) residues of the FGF1 part of the GFPp-FGF1_HS_ fusion protein constitutes the region responsible for the direct recognition of HS (Supplementary Figure 8). HS-affinity chromatography with a linear salt gradient revealed that the higher the valency of GFPp-FGF1_HS_, the stronger the affinity for HS (Figure 1i). Based on the high production yield required in subsequent steps and the high affinity for HS, we decided to focus on the trivalent TriF_HS_. Drug carriers must be to sustain precise drug delivery. We incubated TriF_HS_ in serum and, using SDS-PAGE (Figure 1j), native PAGE (Figure 1k) and fluorimetry (Figure 1l) we demonstrated that the protein is stable and retains its oligomeric state for up to 96 h. Denaturation experiments in conjunction with CD spectroscopy revealed that TriF_HS_ is highly stable, with *T*_den_ over 53 °C (Figure 1m).

These data clearly indicate that TriF_HS_ is a high-affinity trivalent ligand that is highly stable and HSPG-specific.

### HSPGs Are Endocytic Receptors for TriF_HS_

The multivalent design of TriF_HS_ was intended to enable formation of highly stable HSPG clusters on the cell surface that could stimulate rapid and efficient internalization of the TriF_HS_/HSPG complex. To ensure highly effective intracellular drug delivery, we decided to employ the recently discovered aggregation-dependent endocytic (ADE) pathway (Figure 2a).^6,9,10^ We measured the internalization of BiF_HS_, TriF_HS_, and TetraF_HS_ into hTERT-HPNE cells using quantitative confocal microscopy (Figure 2b). The highest endocytic efficiency was observed for TriF_HS_ (Figure 2b). We also showed that internalization of BiF_HS_, TriF_HS_, and TetraF_HS_ is fully abolished by the presence of soluble HS (Figure 2b). On the basis of the highest efficiency of endocytosis, we mainly focused on TriF_HS_ in subsequent studies. We used BaF3 cells devoid of cell-surface HS and demonstrated that these cells are not capable of TriF_HS_ internalization (Figure 2c). Using heparinase III, we depleted the surface of hTERT-HPNE cells of HS and demonstrated that TriF_HS_ endocytosis was blocked in heparinase-III-treated cells (Figure 2d). hTERT-HPNE cells pretreated with sodium chlorate, an inhibitor of HS biosynthesis, were unable to internalize TriF_HS_ (Figure 2e). We also performed endocytosis measurements in the presence of surfen, which is a small-molecule antagonist of HS. As shown in Figure 2f, surfen fully abolished the cellular uptake of TriF_HS_. Because of differences in monosaccharide composition, uronic acid epimerization, and sulfation patterns, HS, dermatan sulfate (DS), and chondroitin sulfate (CS) exhibit distinct spatial distributions of negative charges along their polymer chains. Using BLI, we demonstrated that TriF_HS_ directly binds only HS, not CS or dermatan sulfate (DS) (Figure 2g). We also measured the efficiency of TriF_HS_ endocytosis into hTERT-HPNE cells in the presence of HS, CS, and DS. As shown in Figure 2h, only soluble HS was able to efficiently inhibit TriF_HS_ internalization. Additionally, we treated hTERT-HPNE cells with chondroitinase ABC, an enzyme that preferentially removes CS and DS, leaving HS mostly intact. The activity of chondroitinase ABC (confirmed with chondroitin staining) had no effect on the cellular uptake of TriF_HS_ (Figure 2i). We also performed competition assays with recombinant GPC4 to further assess specificity of TriF_HS_ internalization. The presence of recombinant soluble GPC4 efficiently blocked endocytosis of TriF_HS_ into hTERT-HPNE cells (Figure 2j). To study the potential contribution of N-linked glycans to TriF_HS_ internalization, we treated hTERT-HPNE cells with PNGase F. We used galectin-3, a human lectin that binds N-glycosylated glycoconjugates, as a control to monitor the effectiveness of N-glycan enzymatic removal. To this end, we prepared recombinant galectin-3 and fluorescently labeled it with Alexa-488 dye.^8,25^ Using confocal microscopy, we demonstrated that removal of N-glycans with PNGase F was highly effective, as the cell-surface binding and endocytosis of galectin-3 were nearly fully blocked (Figure 2k). In contrast, treatment of cells with PNGase F had no effect on the cellular uptake of TriF_HS_ (Figure 2k).

**Figure 2:**
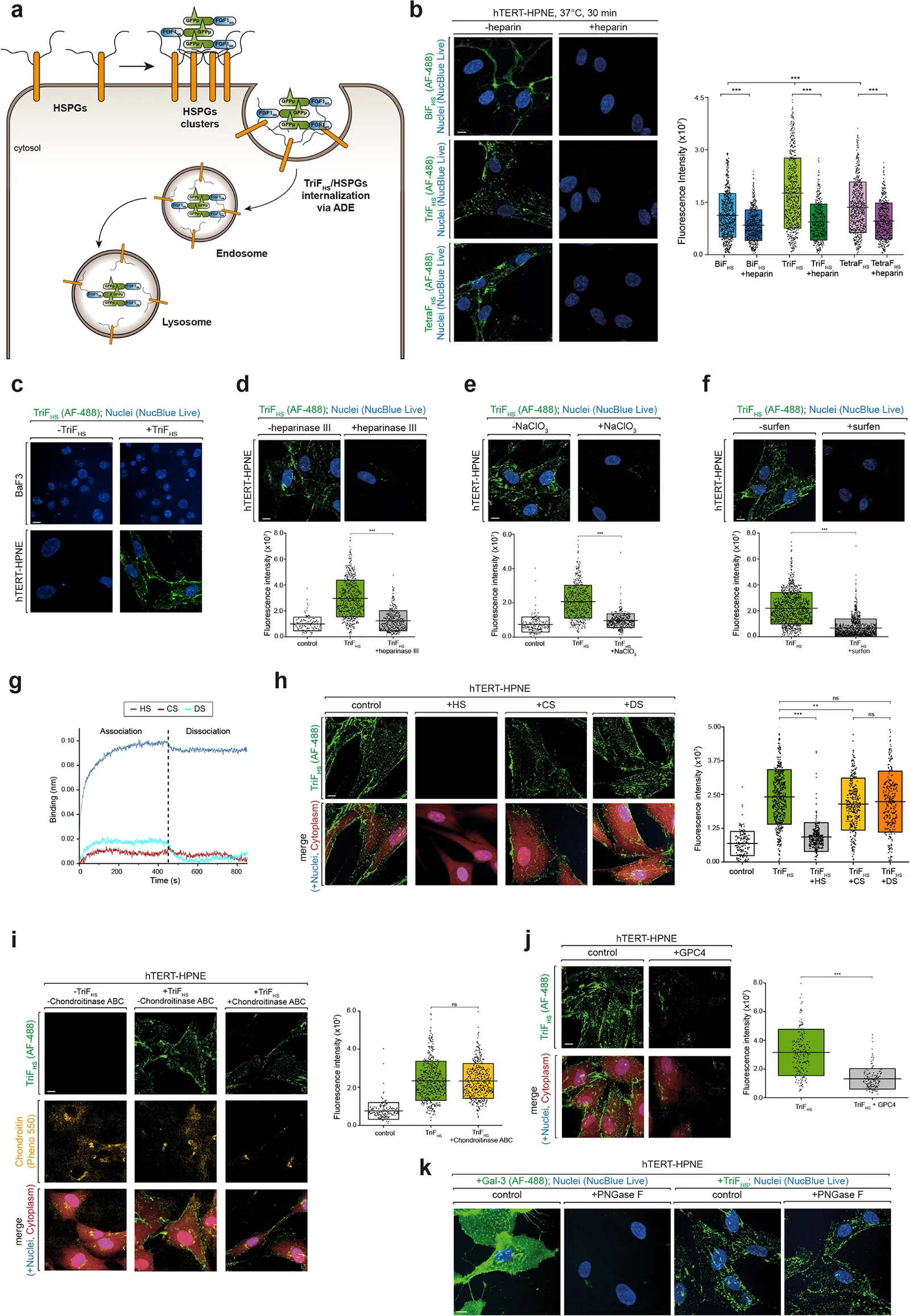
HSPGs constitute highly specific endocytic receptors for TriF_HS_. **a.** Schematic representation of TriF_HS_ internalization via the ADE pathway. TriF_HS_ triggers clustering of HSPGs on the cell surface and induces ADE of TriF_HS_/HSPG complexes. **b.** hTERT-HPNE cells treated with BiF_HS_, TriF_HS_, and TetraF_HS_ (Alexa 488) with or without heparin treatment. Nuclei were stained using NucBlue Live. Intracellular fluorescence intensity was measured in the Alexa 488 channel. Average values ± 1 SD are shown. For each group, a minimum of 457 cells was analyzed. Statistical analysis was performed using two-way ANOVA followed by Fisher’s LSD post hoc test (*p < 0.05, **p < 0.01, ***p < 0.001), *n* = 3. **c.** hTERT-HPNE and BaF3 cells were treated with TriF_HS_ or left untreated (control), *n* = 3. **d.** hTERT-HPNE cells were pretreated with heparinase III or left untreated (control), followed by TriF_HS_ treatment. For each group, a minimum of 139 cells was analyzed, *n* = 3. **e.** hTERT-HPNE cells were pretreated with sodium chlorate or left untreated (control), prior to TriF_HS_ addition. For each group, a minimum of 203 cells was analyzed, *n* = 3. **f.** hTERT-HPNE cells were pretreated with surfen or left untreated (control), prior to TriF_HS_ treatment. For each group, a minimum of 1128 cells was analyzed, *n* = 3. **g.** Analysis of TriF_HS_ binding to HS (blue), CS (red), and DS (cyan) using BLI. **h.** hTERT-HPNE cells were treated with HS, DS, or CS in combination with TriF_HS_. For each group, a minimum of 1128 cells was analyzed, *n* = 3. **i.** Microscopy images of hTERT-HPNE cells pretreated with chondroitinase ABC or left untreated (control), prior to TriF_HS_ addition. An anti-chondroitin antibody was used to verify the effectiveness of chondroitinase ABC treatment. For each group, a minimum of 170 cells was analyzed, *n* = 3. **j.** Microscopy images of hTERT-HPNE cells treated with TriF_HS_ (control) or GPC4 in combination with TriF_HS_. For each group, a minimum of 145 cells was analyzed, *n* = 3. **k.** Microscopy images of hTERT-HPNE cells pretreated with PNGase F or left untreated (control), prior to TriF_HS_ or galectin-3 treatment, *n* = 3. Statistical analyses were performed using analysis of variance with Tukey HSD test (panels d, e, h, (i) (*p < 0.05; **p < 0.005; and ***p < 0.001) or Student’s *t* test (panels f, j) (*p < 0.05; **p < 0.005; and ***p < 0.001). Scale bar represents 10 μm.

Collectively, these data indicate that TriF_HS_ enters cells in a highly specific manner through HSPG-dependent endocytosis.

### Multivalency of TriF_HS_ Induces Ultrafast Uptake of HSPGs via ADE

To investigate whether TriF_HS_ is capable of HSPG clustering *in vitro*, we employed particle sizing with dynamic light scattering (DLS) and a model recombinant HSPG, GPC4 (Figure 3a). Upon mixing TriF_HS_ with GPC4, we identified high-molecular-weight complexes, which were virtually absent in TriF_HS_ or GPC4 samples (Figure 3a). To analyze whether TriF_HS_ triggers HSPG aggregation on the surface of model cells, we incubated TriF_HS_ with hTERT-HPNE cells for 30 min and monitored the subcellular localization of TriF_HS_ (using its intrinsic fluorescence) and GPC4 (with immunofluorescence) using confocal microscopy. In control, TriF_HS_-untreated cells, we observed a uniform distribution of the GPC4 signal on the cell surface and no punctate intracellular signal (Figure 3b). Supplementation of cells with TriF_HS_ caused a rapid disappearance of the diffuse GPC4 signal on the cell surface and the concomitant prompt appearance of multiple TriF_HS_/GPC4-positive puncta on the cell surface (Figure 3b). These results indicate GPC4 clustering by TriF_HS_, and TriF_HS_/GPC4-positive intracellular spots suggest efficient TriF_HS_/GPC4 endocytosis (Figure 3b). We also demonstrated that the intracellular signal of TriF_HS_ largely colocalizes with the marker of early endosomes (EEA1) and with GPC4, which is in agreement with the proposed GPC4 targeting to endosomes (Figure 3c, Supplementary Figure 9).^26^ TriF_HS_ punctate signal was surrounded by the “donut-shaped” signals of EEA1 and GPC4, which reflects the membrane topology of the proteins studied in endosomes (EEA1 and GPC4 are integral membrane proteins, whereas TriF_HS_ is a soluble protein that binds GPC4) (Figure 3c). As a potential drug carrier for the PDC approach, TriF_HS_ should ultimately traffic to lysosomes for proteolytic degradation and intracellular drug release. Indeed, we demonstrated that, as early as 2 h, TriF_HS_ colocalizes with the lysosomal marker LAMP1 (Figure 3d, Supplementary Figure 9).

**Figure 3:**
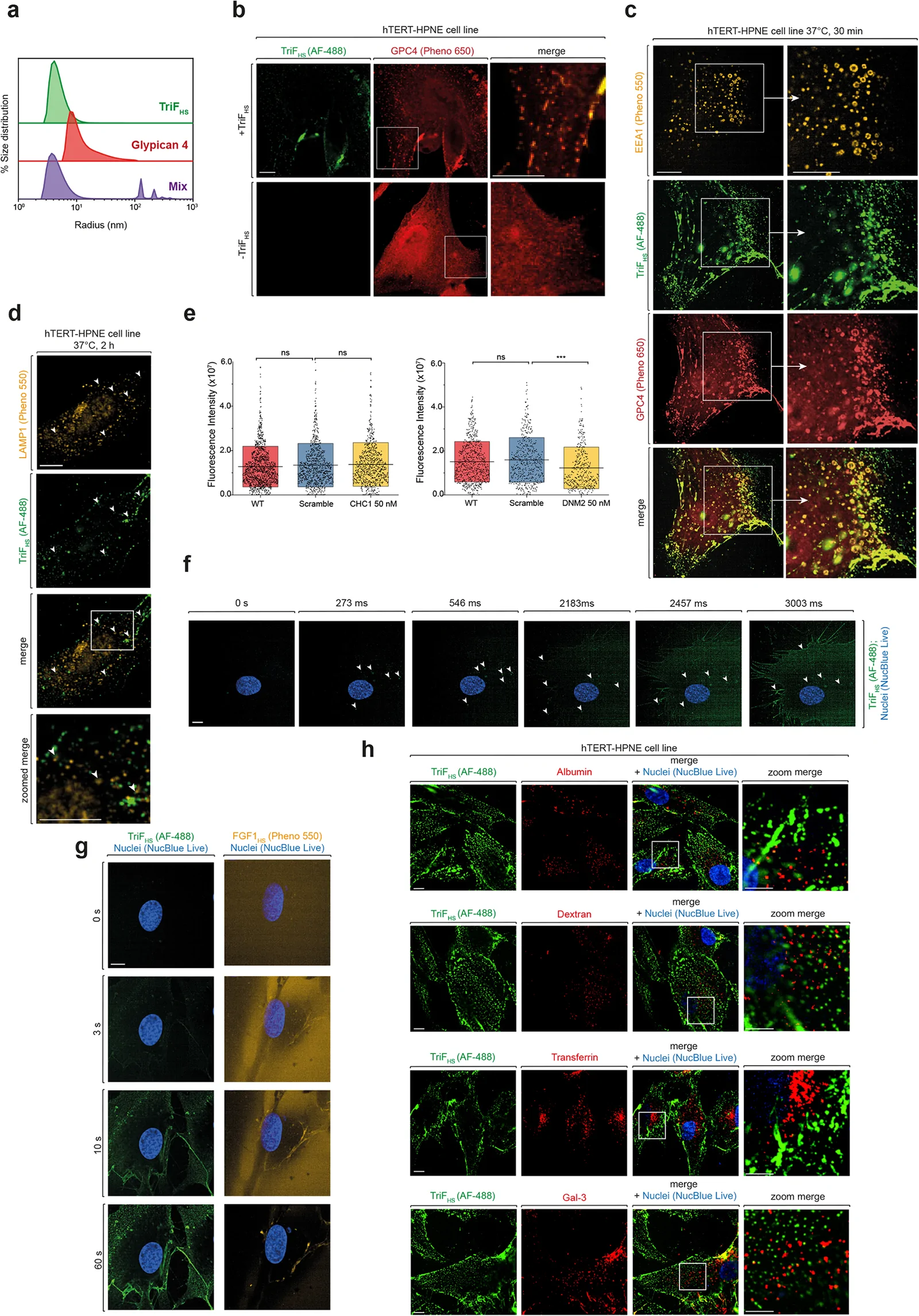
TriF_HS_ triggers ultrafast endocytosis of HSPGs via the ADE pathway. **a.** DLS measurements of the % size distribution of TriF_HS_ (green), GPC4 (red), and the mixture (violet). **b.** HSPG distribution without and with the addition of TriF_HS_ to the hTERT-HPNE cell line. Cells were incubated without or with TriF_HS_ for 30 min at 37 °C, fixed, and GPC4 was detected by immunolabeling (Pheno 650). **c.** Co-localization of TriF_HS_ with GPC4 and the early endosome marker protein EEA1. hTERT-HPNE cells were incubated with TriF_HS_ for 30 min at 37 °C, cells were fixed, and GPC4 (Pheno 650) and EEA1 (Pheno 550) were detected with immunolabeling. **d.** Co-localization of TriF_HS_ with the lysosome marker LAMP1. hTERT-HPNE cells were incubated with TriF_HS_ for 2 h at 37 °C; cells were fixed, and LAMP1 (Pheno 550) was detected with immunolabeling. **e.** Silencing of CHC1 and DNM2. Cells were treated with TriF_HS_ for 30 min at 37 °C and analyzed with confocal microscopy. Fluorescence intensity (Alexa 488) was measured in each cell and compared between cells treated and untreated with siRNA against CHC1 or DNM2. WT – cells untreated (red); Sc – scramble, cells treated with nontargated siRNA (blue); CHC1 50 nM – cells treated with 50 nM siRNA against CHC1 (yellow). For experiments with CHC1 silencing, a minimum of 796 cells per group were analyzed, *n* = 3. For experiments with DNM2 silencing, a minimum of 397 cells per group were analyzed, *n* = 3. Statistical analyses were performed using analysis of variance with Tukey’s HSD test (*p < 0.05; **p < 0.005; and ***p < 0.001). **f.** Internalization kinetics of TriF_HS_ (Alexa 488) in hTERT-HPNE cells. Nuclei were stained using NucBlue Live. Images were captured every 273 ms. **g.** Internalization kinetics of TriF_HS_ (Alexa 488) and FGF1_HS_ (Pheno 550) in hTERT-HPNE cells. Nuclei were stained using NucBlue Live. Images were captured every 1 s. **h.** Co-localization of TriF_HS_ with fluorescently labeled model cargoes of distinct endocytic pathways. hTERT-HPNE cells were incubated with TriF_HS_ and model cargoes (transferrin, galectin-3, dextran, and albumin) for 30 min at 37 °C; cells were fixed. Cells were analyzed with confocal microscopy. Statistical analyses were performed across at least three independent experiments. Scale bar represents 10 μm.

Recent reports indicate that ADE is clathrin-independent.^6,9^ Using siRNA technology, we efficiently knocked down clathrin heavy chain 1 (CHC1), a major component of clathrin-mediated endocytosis (CME; Supplementary Figure 10a) and studied the impact of CME downregulation on the efficiency of TriF_HS_ internalization. As shown in Figure 3e and Supplementary Figure 10a, downregulation of CHC1 had no impact on TriF_HS_ endocytosis. In contrast, silencing dynamin-2, a protein involved in CME and also in several clathrin-independent endocytic (CIE) pathways, partially blocked the cellular uptake of TriF_HS_ (Figure 3e, Supplementary Figure 10b).

To study the kinetics of TriF_HS_ internalization, we employed a quantitative confocal microscopy platform, the Opera Phenix Plus, in a setup that allowed imaging of ultrafast cellular processes. Indeed, we demonstrated that TriF_HS_ undergoes extremely fast endocytosis, with an intracellular signal observed as early as 273 ms (Figure 3f). We then compared the internalization kinetics of trivalent TriF_HS_ with monovalent FGF1_HS_ and showed that, within the measured timescale, FGF1_HS_ displayed only binding to the cell surface, with no detectable endocytosis (Figure 3h). We also observed that the massive accumulation of TriF_HS_ on the cell surface was associated with the appearance of numerous endocytic vesicles (Supporting Information Movie S1).

Endocytosis operates through several distinct molecular mechanisms, each utilizing specific membrane structures and coat proteins to internalize external materials and pathway-specific cargoes.^27^ To study the mechanism used by TriF_HS_ for cell entry, we examined potential cointernalization of TriF_HS_ with fluorescently labeled model cargoes of distinct endocytic pathways:^27^ transferrin (a model cargo of CME),^28^ galectin-3 (a protein involved in the clathrin-independent carriers/glycosylphosphatidylinositol-attached protein-enriched endosomal compartments (CLIC/GEEC),^29^ dextran (a cargo specific for macropinocytosis^30^ and albumin (a cargo that uses clathrin-independent endocytosis (CIE) or CME for cell entry^31,32^ ). None of the tested cargoes colocalized with TriF_HS_, confirming that TriF_HS_ is internalized predominantly via the ADE pathway (Figure 3h).

These data indicate that, due to its multivalency, TriF_HS_ is capable of clustering HSPG on the cell surface, which boosts ultrafast TriF_HS_/HSPG endocytosis via ADE and efficiently delivers TriF_HS_/HSPG to lysosomes.

### Development of Multivalent PDC Targeting HSPGs

Based on tight and selective HSPG binding and the capability to trigger highly efficient HSPG-mediated ADE, we employed multivalent TriF_HS_ as a cytotoxic drug carrier and developed a novel PDC strategy targeting HSPGs overexpressed by pancreatic cancer cells (Figure 4a). To covalently couple the highly potent cytotoxic drug monomethyl auristatin E (MMAE) to TriF_HS_ in a site-specific manner, we utilized an enzymatic ligation strategy using Sortase A. Sortase A recognizes the LPETGG sequence incorporated into the C-terminus of TriF_HS_ and mediates the ligation of a peptide linked to MMAE (Figure 4b). We synthesized the peptide and conjugated it to maleimide-vcMMAE, resulting in GGGS-MMAE (Supplementary Figure 11). Next, we optimized the conditions for drug attachment to TriF_HS_ and obtained TriF_HS_-MMAE conjugate, which we verified by Western blotting with MMAE-specific antibodies (Figure 4c). Additionally, we assessed the conjugate purity by size-exclusion chromatography (SEC) and confirmed its identity by mass spectrometry (MS) (Figure 4d). We confirmed that incorporation of MMAE into TriF_HS_ did not block HSPG binding (Supplementary Figure 12) or HSPG-dependent endocytosis (Supplementary Figure 13).

**Figure 4:**
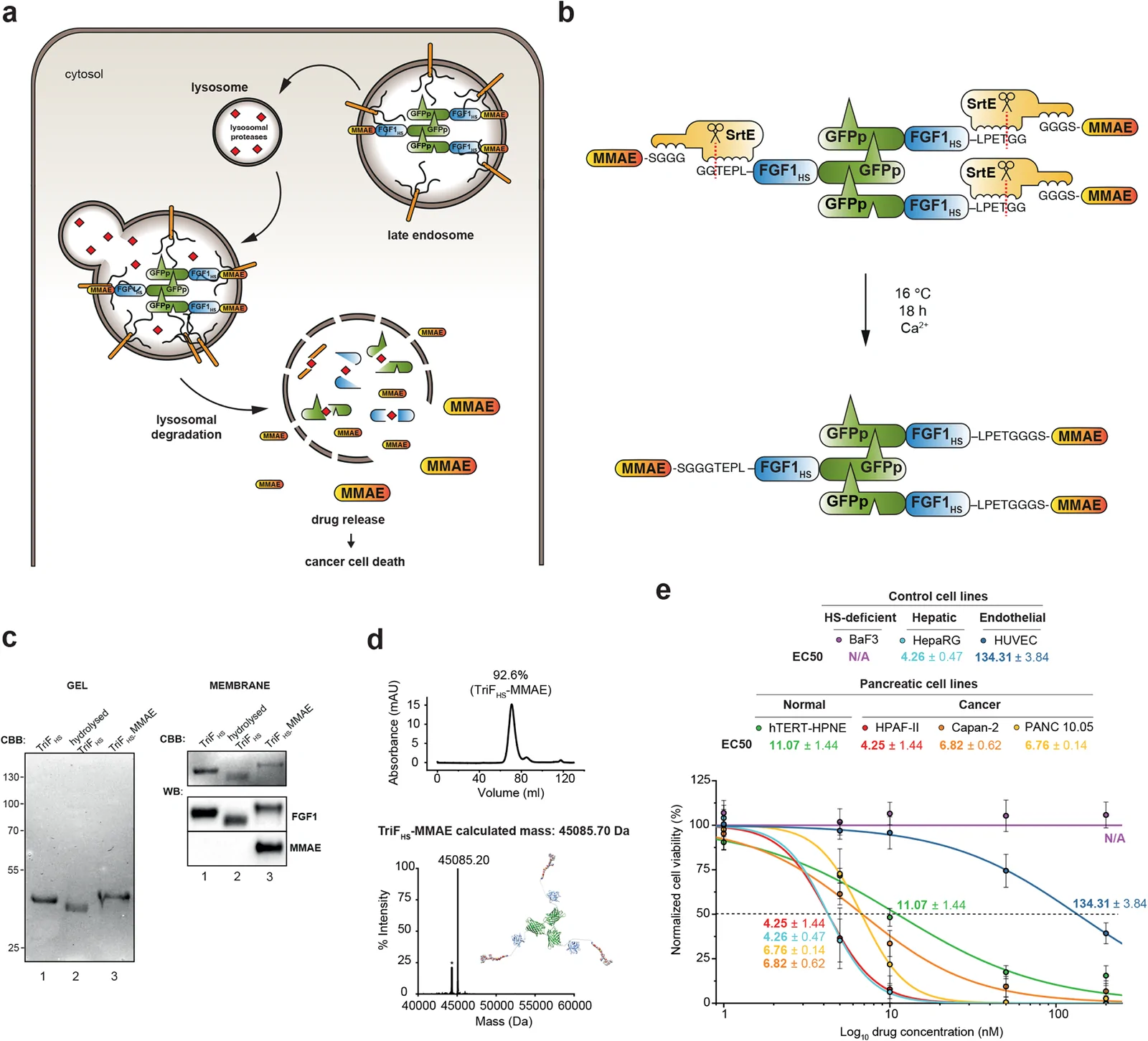
TriF_HS_-MMAE is a potent, HSPG-specific protein–drug conjugate exhibiting high cytotoxicity toward pancreatic cancer cells. **a.** Schematic illustration of TriF_HS_-MMAE internalization via HSPG-mediated uptake and subsequent induction of apoptosis in target cells. **b.** Generation of TriF_HS_-MMAE through sortase A-mediated site-specific conjugation of a peptide-linked monomethyl auristatin E (MMAE) to the C-terminus of TriF_HS_. **c.** Verification of TriF_HS_-MMAE conjugate formation by SDS-PAGE (left) and Western blot analysis (right). **d.** SEC and mass spectrometric characterization of TriF_HS_-MMAE. Top panel: SEC profile of TriF_HS_-MMAE. The purity of the conjugate was calculated as the percentage of the main TriF_HS_-MMAE peak area relative to the total integrated peak area, yielding a purity of 92.6%. Bottom panel: raw spectra were acquired and processed using MassLynx V4.2 software. Protein peaks were integrated, background-subtracted, and deconvoluted using the MaxEnt1 algorithm. The peak marked with an asterisk (*) corresponds to an in-source fragment generated by conjugate fragmentation during the MS experiment. **e.** Comparative analysis of normalized cell viability following treatment with TriF_HS_-MMAE in a panel of control cell lines and pancreatic cell lines (normal and cancer), with half-maximal effective concentration (EC_50_) values calculated for each line. The Hill model was used for EC_50_ values estimation.

Next, we evaluated the cytotoxicity of TriF_HS_-MMAE in normal pancreatic cells (hTERT-HPNE), normal hepatocytes (HepaRG), normal endothelial cells (HUVECs), model BaF3 cells devoid of cell surface HS, and in a panel of pancreatic cancer cell lines with varying HSPG expression.^33^ As shown in Figure 4e, TriF_HS_-MMAE displayed no toxicity toward BaF3 cells, confirming very high selectivity for HS. The toxicity of TriF_HS_-MMAE toward HUVECs was also minimal. Among the tested pancreatic cell lines, TriF_HS_-MMAE displayed the lowest toxicity for the normal pancreatic cells (IC_50_ = 11.07 nM) (Figure 4e). In contrast, TriF_HS_-MMAE conjugate was able to induce death in a larger pool of malignant cells and achieved this at lower concentrations, with the lowest IC_50_ = 4.25 nM measured for HPAF-II cells (Figure 4e). In the presence of excess HS, TriF_HS_-MMAE was nontoxic to HPAF-II cells, which further highlights its high specificity for HS (Supplementary Figure 14). The TriF_HS_-MMAE conjugate also displayed significant toxicity to hepatocytes, cells known to express high levels of HSPGs (Figure 4e). The results of cytotoxicity tests are consistent with the Western blotting analyses of HSPGs in the studied cells, in which we detected the highest HSPG and GPC4 amounts in the pancreatic cancer cell lines HPAF-II, Capan-2, and CFPac-1 (Supplementary Figure 15).

These data demonstrate that TriF_HS_-MMAE is an HSPG-specific cytotoxic conjugate which, due to the known overexpression of HSPGs on pancreatic cancer cells,^14–16^ displays higher toxicity toward malignant cells than toward healthy cells. As expected, the high abundance of HSPGs on the surfaces of other cell types results in only a moderate difference in TriF_HS_-MMAE toxicity between normal and cancer cells, implying the requirement for further improvement in TriF_HS_-MMAE uptake selectivity through novel approaches.

### HCS Identification of Signaling Pathways That Govern ADE of HSPGs

Next, we aimed to enhance the uptake of TriF_HS_-MMAE by pancreatic cancer cells and, at the same time, downregulate conjugate internalization by normal pancreatic cells. Endocytosis and signaling are mutually tightly linked, which enables coordinated action of the cell and prevents the development of disease due to dysregulated signal transmission, including cancer^34,35^ (Figure 5a). We hypothesized that modulation of partially distinct endocytic and regulatory signaling pathways in normal and cancer cells could increase the selectivity of TriF_HS_-MMAE delivery (Figure 5a). Because the signaling cascades that govern ADE and HSPG endocytosis are largely unknown, we first sought to identify kinases that regulate TriF_HS_/HSPG uptake in normal pancreatic cells to downregulate TriF_HS_-MMAE uptake by healthy cells.

**Figure 5:**
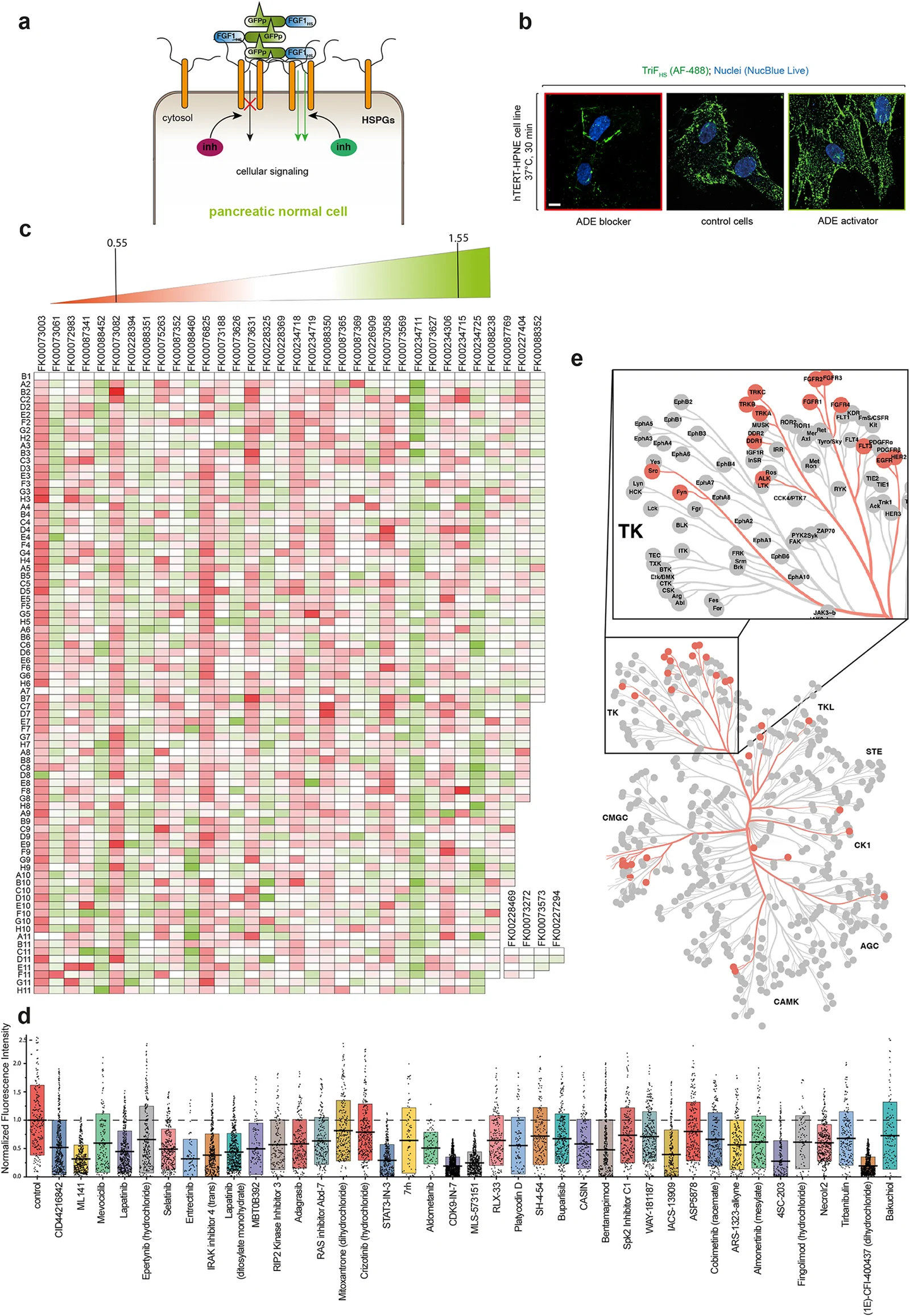
Selection of chemical compounds that affect the efficiency of endocytosis based on High-Content Screening measurements. **a.** Schematic representation of blocking TriF_HS_ internalization via the ADE pathway by an inhibitor. **b.** Examples of the impact of selected compounds on the efficiency of TriF_HS_ internalization in hTER-HPNE cells. Cells were pretreated with an ADE blocker/activator for 30 min at 37 °C, then TriF_HS_ was added, and cells were incubated for 30 min at 37 °C. Nuclei were stained using NucBlue Live. The ADE blocker (red frame) inhibits ADE, whereas the ADE activator (green frame) promotes ADE. **c.** Results from HCS with a library of kinase inhibitors and their impact on TriF_HS_ internalization. hTERT-HPNE cells were pretreated with an inhibitor for 30 min at 37 °C, then TriF_HS_ was added, and cells were incubated for 30 min at 37 °C; cells were fixed and analyzed with confocal microscopy. The fluorescence intensity (Alexa 488) was measured in each cell, the signal was normalized and compared across cells. Results are shown in the form of a microarray, where the color gradient indicates the efficiency of endocytosis (red – inhibition; green – activation). On the left side of the microarray are the well designations, and at the top of the microarray are the names of plates with chemical compounds. Based on normalized fluorescence intensity, inhibitors were selected – blockers with values less than 0.55; activators with values greater than 1.55. **d.** Compounds that significantly decrease TriF_HS_ endocytosis in hTERT-HPNE cells based on HCS. Each dot on the graph corresponds to the fluorescence intensity in one cell. For each group, a minimum of 75 cells were analyzed, *n* = 3. Statistical analyses were performed using analysis of variance with Tukey’s HSD test (*p < 0.05; **p < 0.005; ***p < 0.001). **e.** Kinase tree analysis of the targets (red dots) of selected inhibitors. The major signaling pathways that inhibit TriF_HS_ endocytosis belong mainly to the tyrosine kinase (TK) family. Graph generated in the CORAL application (http://phanstiel-lab.med.unc.edu/CORAL/). Statistical analyses were performed for at least three independent experiments.

For this purpose, we developed a high-content screening (HCS) method employing an Opera Phenix Plus microscopy platform, enabling quantitative measurement of TriF_HS_ internalization into hTERT-HPNE cells (Supplementary Figure 16). The stable intrinsic fluorescence of TriF_HS_ allowed us to conduct HCS experiments that require large quantities of high-quality, fluorescently labeled protein. Initially, we assessed the concentration of TriF_HS_ to allow quantitative microscopic monitoring of changes in the efficiency of TriF_HS_ endocytosis (Supplementary Figure 17). In HCS screens, hTERT-HPNE cells were grown in a multiwell format, pretreated with each of the compounds from the near-kinome-wide library of kinase inhibitors (2653 agents), incubated with TriF_HS_, and subjected to HCS imaging and data analyses (Supplementary Figure 16). The exemplary results of such HCS test are shown in Figure 5b, with model kinase inhibitors that block (left panel, red frame), have no effect (middle panel, white frame), or activate (right panel, green frame) TriF_HS_ endocytosis into hTERT-HPNE cells. To select the most interesting candidates from the library, we set a threshold for the change in intracellular TriF_HS_ signal in relation to the untreated control to less than 0.55 for ADE blockers (red) and more than 1.55 for ADE activators (green), and we developed a matrix based on HCS experiments (Figure 5c). HCS screening of the library revealed that the vast majority of tested compounds (2404) fell between the set boundaries and therefore had no effect on TriF_HS_ endocytosis (Figure 5c). A strong blockade of TriF_HS_ internalization into hTERT-HPNE cells was detected for 118 inhibitors (Figure 5c and Supplementary Table 1), whereas 131 inhibitors promoted the uptake of TriF_HS_ by the cells (Figure 5c and Supplementary Table 2). Subsequent one-way ANOVA statistical analyses narrowed the list of compounds that significantly modulate TriF_HS_ endocytosis to 39 downregulators (Figure 5d, Supplementary Figures 18–21, Supplementary Table 1) and 34 activators (Supplementary Figure 22–26, Supplementary Table 2 and 4).

The human genome encodes over 500 different kinases classified into several major groups, families, and subfamilies.^36^ Functional categorization of the identified kinases revealed that the major signaling pathways that block TriF_HS_ endocytosis via ADE involve specific receptor tyrosine kinases (RTKs) and Rac1-downstream signaling pathways (Figure 5e, Supplementary Table 3). Signaling cascades that promote TriF_HS_ uptake into normal pancreatic cells belong mainly to the CDK, MAPK, GSK, and CLK kinase groups (the CMGC kinase family) (Supplementary Figure 27, Supplementary Table 4). We measured changes in the turbidity of the TriF_HS_ solution over time, in the presence or absence of model-identified modulators of TriF_HS_ endocytosis, in order to evaluate the possibility of TriF_HS_ aggregation in the presence of inhibitors. As shown in Supplementary Figure 28, the tested compounds had no effect on the turbidity of the TriF_HS_ solution, indicating no aggregation of TriF_HS_ or of the inhibitors themselves. To determine whether the identified compounds themselves can affect endocytosis of HSPGs, we treated hTERT-HPNE cells with selected model inhibitors and assessed cellular localization of general HSPGs (anti-HS staining) and the specific HSPG GPC4. In the absence of TriF_HS_, all studied inhibitors had no impact on the internalization of HSPGs, indicating that they are modulators of endocytic events triggered by TriF_HS_ (Supplementary Figure 29). To determine whether detection of TriF_HS_ fluorescence in the HCS screen in the presence of inhibitors indeed monitors specific endocytosis of TriF_HS_/HSPGs complex, we treated hTERT-HPNE cells with TriF_HS_ and model inhibitors and studied colocalization of the intrinsic TriF_HS_ fluorescence with the signal of anti-HS antibodies. We observed colocalization of TriF_HS_ with HS in the presence of the tested inhibitors (Supplementary Figure 30).

These data confirm the unbiased identification of an endocytosis-signaling linkage in pancreatic cells. Importantly, by using the HCS screen, we revealed, for the first time and in an unbiased manner, signaling cascades that control the endocytosis of HSPG.

### Discovery of Signaling Pathways That Differentially Regulate ADE in Normal and Pancreatic Cancer Cells

Initially, we assessed whether TriF_HS_ triggers clustering of HSPGs on the surface of pancreatic cancer cells as it does on normal pancreatic cells. Supplementation of pancreatic cancer cells with TriF_HS_ induced changes in the GPC4 distribution, from a diffuse signal on the cell surface to bright puncta that colocalized with TriF_HS_, indicating effective TriF_HS_-mediated clustering of HSPGs on the surface of pancreatic cancer cells (Supplementary Figure 31). Next, we searched among the 39 identified inhibitors of TriF_HS_ endocytosis for compounds that either have no effect on TriF_HS_ internalization or, preferably, even stimulate TriF_HS_ endocytosis in pancreatic cancer cells, potentially increasing TriF_HS_-MMAE selectivity (Figure 6a). To this end, we pretreated normal pancreatic cells and four pancreatic cancer cell lines—HPAF-II, Capan-2, CFPAC 1 , and PANC-10.05—with the 39 identified compounds that block TriF_HS_ internalization into normal pancreatic cells and measured their impact on TriF_HS_ endocytosis in pancreatic cancer cells using high-content quantitative confocal microscopy. As shown in Figure 6b, the full spectrum of effects was detected, including compounds acting as universal endocytosis inhibitors in all studied cell lines (e.g., ARS-1323-alkyne) or exerting mixed effects on internalization in different cancer cell lines. Importantly, we identified nine agents that acted as inhibitors of TriF_HS_ internalization in normal cells and concomitantly displayed either no effect or stimulated TriF_HS_ endocytosis in at least three out of four tested pancreatic cancer cell lines (Figure 6b,c, Supplementary Figure 32).

**Figure 6:**
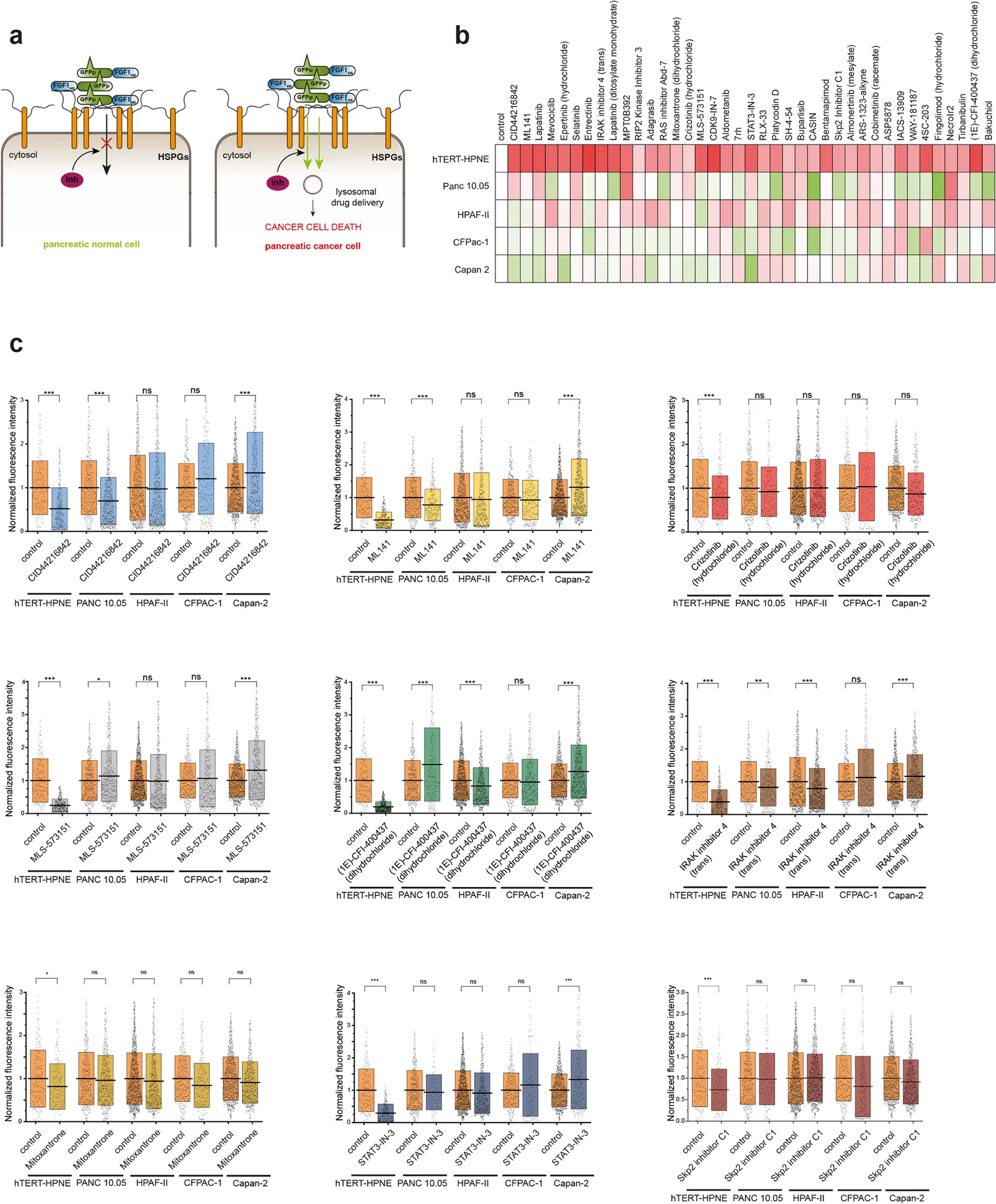
Selection of chemical compounds that differentially regulate ADE in normal and cancer pancreatic cells. **a.** Schematic representation of the action of the same chemical compounds in normal and cancer pancreatic cells. In normal pancreatic cells, the inhibitor blocks TriF_HS_ internalization via the ADE pathway, and at the same time induces ADE-dependent internalization in pancreatic cancer cells, which leads to lysosomal drug delivery and cell death. **b.** Results from HCS with selected compounds and their impact on TriF_HS_ internalization in normal pancreatic cells (hTERT-HPNE) and pancreatic cancer cells (PANC 10.05, HPAF-II, CFPac-I, Capan 2). Cells were pretreated with an inhibitor for 30 min at 37 °C, then TriF_HS_ was added, and cells were incubated for 30 min at 37 °C; cells were fixed and analyzed with confocal microscopy. The fluorescence intensity (Alexa 488) was measured in each cell, the signal was normalized and compared across cells. Results are shown in the form of a microarray, where the color gradient indicates the efficiency of endocytosis (red – inhibition; green – induction). **c.** Selection of nine inhibitors that significantly inhibit internalization in normal cells (hTERT-HPNE cell line) and have no impact or significantly promote internalization in pancreatic cancer cells (PANC 10.05, HPAF- II, CFPac-I, Capan 2 cell lines). For each group, a minimum of 119 cells were analyzed, *n* = 3. Statistical analyses were performed using analysis of variance with Tukey’s HSD test (*p < 0.05; **p < 0.005; ***p < 0.001). Statistical analyses were performed for at least three independent experiments.

Our findings imply selective, kinase-dependent control of TriF_HS_/HSPG endocytosis, as, from over 500 kinases present in the human kinome, the identified inhibitors predominantly target RTKs and their specific downstream effectors: HGFR, STAT3, Cdc42 (3 different compounds), Toll-like receptors (IRAK4), the proteasomal system (Usp11, Skp2), and PLK4 (Figure 6c). We demonstrated that none of these nine compounds alone had an impact on the subcellular localization of general HSPGs or the specific HSPG GPC4 in the absence of TriF_HS_ in pancreatic cancer cells, as was also observed in normal pancreatic cells (Supplementary Figure 33). We also showed that, under the conditions applied for measurements of TriF_HS_/HSPG endocytosis, none of the identified inhibitors had a negative impact on the viability of either normal or malignant pancreatic cells (Supplementary Figure 34).

Next, we assessed the effect of nine kinase inhibitors identified by us as differential TriF_HS_/HSPG ADE modulators in normal and pancreatic cancer cells on other cellular endocytic pathways. To this end, cells were pretreated with inhibitors, incubated with fluorescently labeled cargoes of specific endocytic routes, and the impact of inhibitors on cargo internalization was measured with quantitative confocal microscopy. ML141, STAT3-IN-3, and MLS-573151 blocked cellular uptake of transferrin, galectin-3, albumin, and dextran, indicating that these compounds act as general downregulators of endocytosis in pancreatic cells (Supplementary Figure 35). CID44216842 inhibited internalization of transferrin, albumin, and galectin-3, but had no effect on the uptake of dextran, implying that this compound simultaneously blocks ADE of HSPGs, CME, and CLIC/GEEC, but has no impact on macropinocytosis (Supplementary Figure 35). (1E)-CFI-400437 blocked only the uptake of galectin-3, indicating that, besides ADE of HSPGs, it may also affect the CLIC/GEEC pathway (Supplementary Figure 35). Importantly, four compounds, IRAK inhibitor 4, crizotinib (hydrochloride), mitoxantrone, and Skp2 inhibitor C1, had no significant impact on the uptake of the tested model cargoes of diverse endocytic pathways, implying that these inhibitors are highly specific and block ADE internalization of TriF_HS_/HSPG (Supplementary Figure 35).

Next, we evaluated whether increased selectivity of TriF_HS_ cellular targeting, achieved through rational signaling-based manipulation of endocytosis, is reflected in the elevated selectivity of the TriF_HS_-MMAE conjugate (Figure 7a). To this end, normal (hTERT-HPNE) and malignant pancreatic cells (HPAF-II, pancreatic cancer cells chosen due to their highest susceptibility to TriF_HS_-MMAE (Figure 4e)) were treated with nine HCS-identified inhibitors that had differing effects on normal and pancreatic cancer cells and with the TriF_HS_-MMAE conjugate. We observed a differential impact on the viability of normal and malignant pancreatic cells for some inhibitors. Out of nine tested compounds, four, crizotinib (hydrochloride), MLS-573151, CID44216842, and ML141, displayed mitogenic activity for normal hTERT-HPNE pancreatic cells and at the same time were highly toxic for HPAF-II pancreatic cancer cells (Figure 7b, Supplementary Figure 36). For combinations of TriF_HS_-MMAE with six of the nine tested ADE modulators, we observed a lack of cytotoxicity in the normal pancreatic cells and moderate (IRAK inhibitor 4 and (1E)-CFI-400437 (dihydrochloride)) to high (CID44216842, ML141, crizotinib (hydrochloride), MLS-573151) toxicity for the HPAF-II pancreatic cancer cell line (Supplementary Figure 36). We demonstrated that the toxicity of free MMAE, a drug that freely crosses the membrane without major involvement of endocytic processes, is not significantly modulated by the selected ADE modulator, crizotinib (hydrochloride) (Supplementary Figure 37).

**Figure 7:**
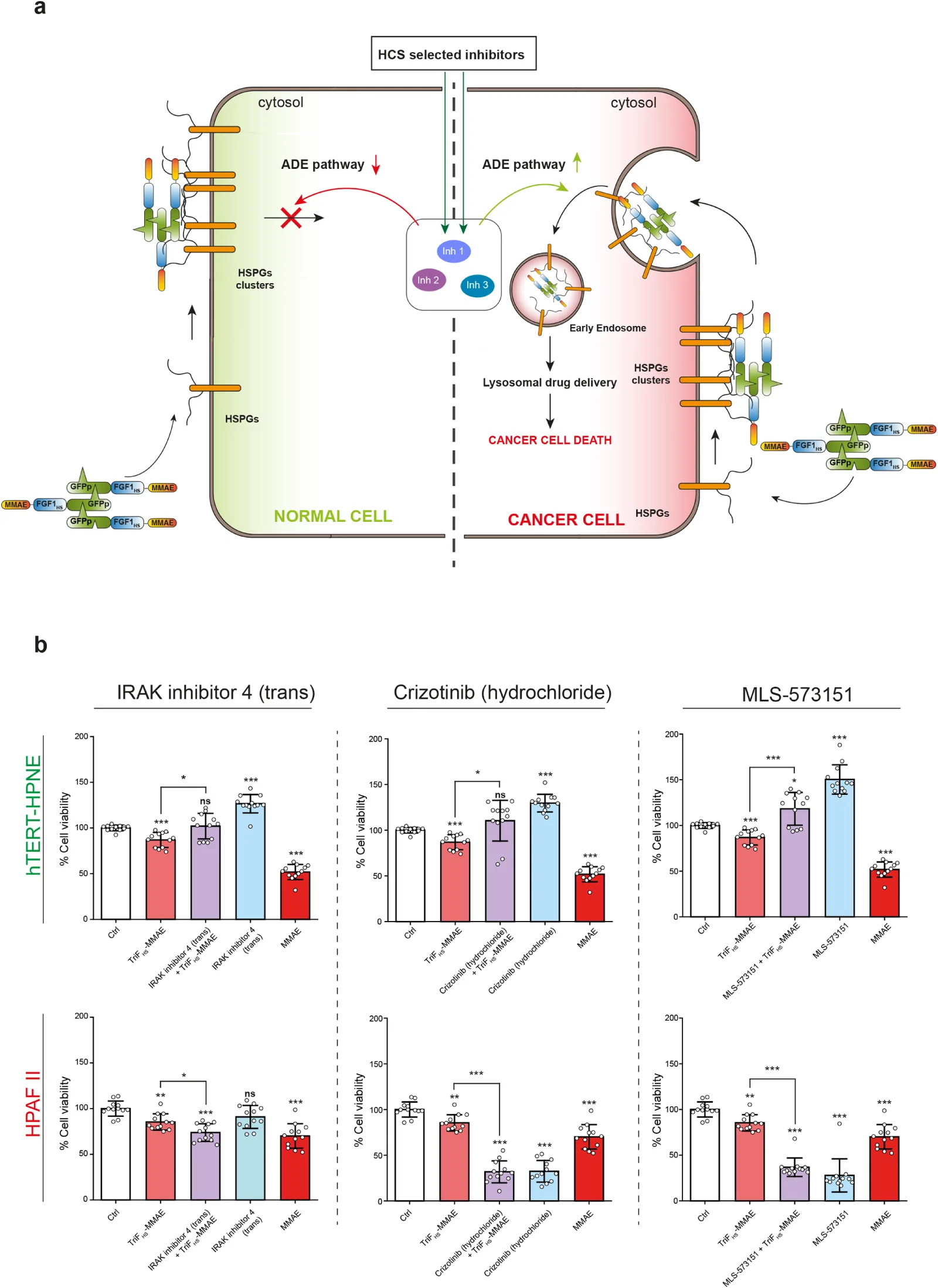
Rational modulation of TriF_HS_ triggered ADE using selected inhibitors *in vitro*. **a.** Schematic model of the rational modulation of ADE of TriF_HS_-MMAE by ADE-regulating signaling pathways for the improved selectivity of conjugate delivery. The trivalent TriF_HS_-MMAE specifically and very strongly binds HSPGs on the surface of cells and induces their clustering within the plasma membrane, thereby triggering ultrafast , highly efficient internalization and lysosomal targeting of TriF_HS_-MMAE/HSPG via the ADE pathway. Since HSPGs are overproduced by pancreatic cancer cells, ADE sustains more efficient TriF_HS_-MMAE delivery, which results in higher TriF_HS_-MMAE toxicity for malignant cells than for normal ones. To further improve the selectivity of drug delivery, signaling pathways that control ADE were identified, and it was demonstrated that ADE regulation differs in normal and cancer cells. Signaling modulators are then used to “teach” normal cells to internalize TriF_HS_-MMAE less efficiently and, at the same time endocytose TriF_HS_-MMAE with the same or even higher efficiency in cancer cells, improving the selectivity of the conjugate. **b**. Inhibitors have different impacts on TriF_HS_-MMAE action in normal and cancer pancreatic cells. Cells were pretreated with an inhibitor for 30 min at 37 °C; then TriF_HS_ was added, and cells were incubated for 24 h at 37 °C; afterward, cells were analyzed by confocal microscopy. Propidium iodide solution was added to visualize dead cells, and NucBlue Live was added to live cells. The numbers of total cells and cells undergoing apoptosis were determined using DAPI and propidium iodide, respectively. The number of dead cells was subtracted from the total number of nuclei stained with NucBlue. Each dot on the graph corresponds to a single cell. Ctrl – cells untreated; TriF_HS_-MMAE – cells treated with TriF_HS_-MMAE; Name of compound + TriF_HS_-MMAE – cells pretreated with the compound and then treated with TriF_HS_-MMAE; Name of compound – cells treated with that downregulator; MMAE – cells treated with MMAE. Results were normalized to 100% cell viability for control cells. Statistical analyses were performed using analysis of variance with the Games-Howell test (*p < 0.05; **p < 0.005; ***p < 0.001; *n* = 3). Statistical analyses were performed for at least three independent experiments.

After intracellular delivery, MMAE binds tubulin, resulting in destabilization of microtubules that precedes cell death (Supplementary Figure 36). Therefore, we analyzed the impact of a representative ADE modulator, crizotinib (hydrochloride), on the destabilization of microtubules by free MMAE (which reaches intracellular targets by diffusion) and by TriF_HS_-MMAE (which enters cells via HSPG-dependent ADE). The destabilizing effect of free MMAE was not abolished by pretreatment of cells with crizotinib (hydrochloride) (Supplementary Figure 38). In contrast, alterations in microtubule organization triggered by TriF_HS_-MMAE were partially restored by priming cells with crizotinib (hydrochloride) (Supplementary Figure 38).

These data indicate that signaling control over HSPG endocytosis via ADE differs between normal and malignant pancreatic cells. Importantly, we discovered highly specific chemical modulators of cellular signaling that elevate the selectivity of TriF_HS_ delivery via ADE between normal and malignant pancreatic cells. Furthermore, our experiments identified five kinase inhibitors with high standalone therapeutic potential for pancreatic cancer. These inhibitors, either alone or in combination with TriF_HS_-MMAE, display highly selective toxicity, preserving the viability of normal pancreatic cells while effectively inducing the death of pancreatic cancer cells.

### *In Vivo* Evaluation of a Rational Approach to Control Endocytosis through Cellular Signaling to Minimize Off-Target Effects of TriF_HS_-MMAE

We evaluated a strategy to rationally control the endocytosis of TriF_HS_-MMAE by manipulating cellular signaling in order to minimize its toxicity to healthy organs *in vivo* (Figure 8a). We successfully scaled up the production of the TriF_HS_-MMAE cytotoxic conjugate to obtain high yields (several dozen milligrams), high purity, and an endotoxin-free product suitable for *in vivo* studies in a mouse model.

**Figure 8:**
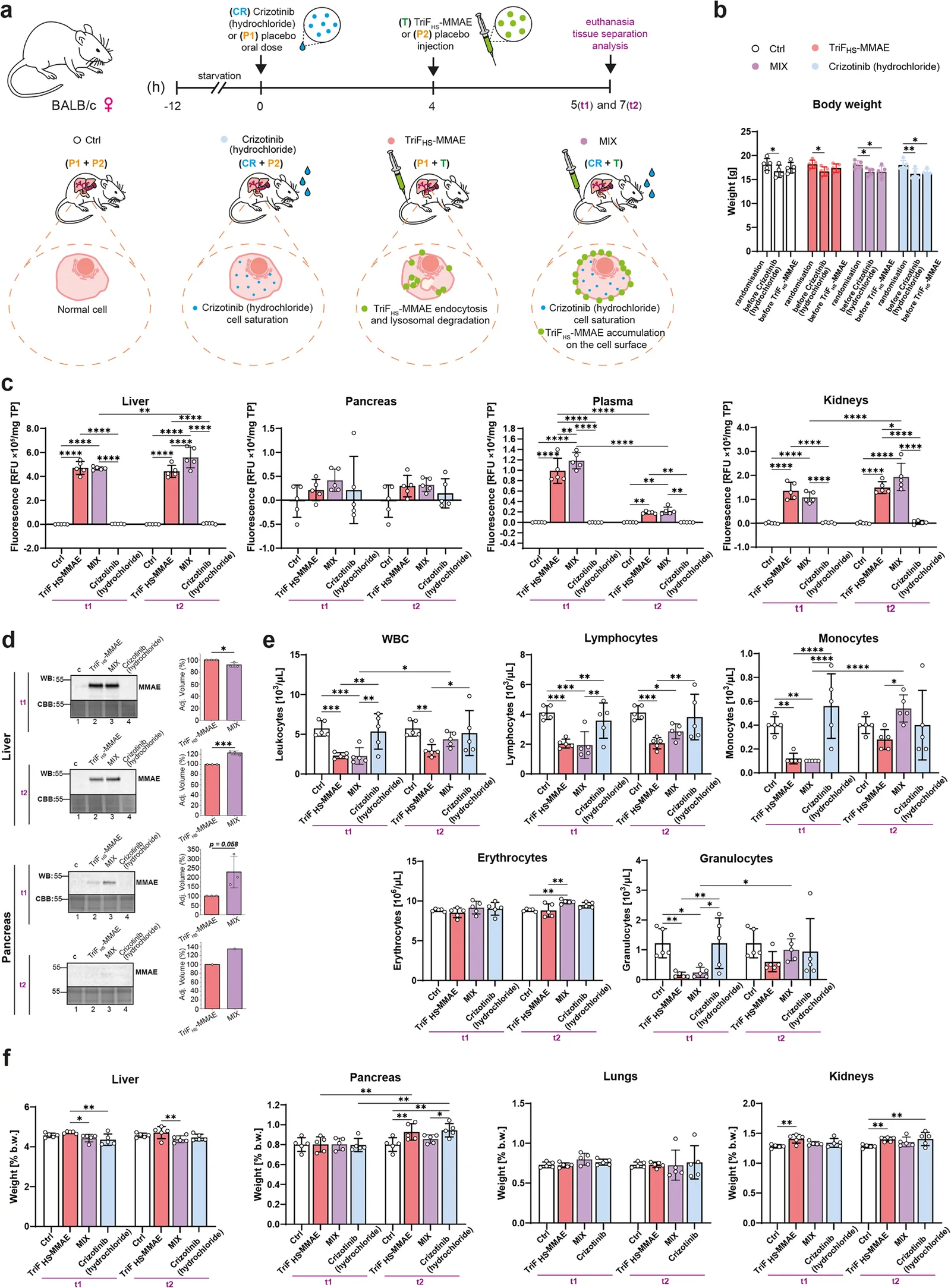
Selected inhibitor crizotinib (hydrochloride) acts as an ADE modulator, impairing the endocytosis of TriF_HS_-MMAE in healthy mice. **a.** Schematic representation of the mouse experiment. Each group in this study was represented by 5 mice, with two euthanasia time points—at 1 h (t1) and 3 h (t2) after treatment with TriF_HS_-MMAE. **b.** Body weight of mice before TriF_HS_-MMAE and crizotinib (hydrochloride) administration. **c.** Fluorescence-based biodistribution of TriF_HS_-MMAE following crizotinib coadministration. Fluorescence intensity derived from TriF_HS_-MMAE conjugate was quantified in plasma, pancreas, liver, and kidneys at t1 and t2 after treatment with TriF_HS_-MMAE. Raw fluorescence values were normalized to total protein content and background-subtracted using tissues and plasma from PBS-treated control animals processed in the same experimental batch. Data are presented as mean ± SD with individual data (*n* = 5 per group). Statistical analysis was performed using two-way ANOVA followed by Fisher’s LSD post hoc test. *p < 0.05, **p < 0.01, ***p < 0.001, ****p < 0.0001. **d.** Western blotting of liver and pancreas samples from mice in the experiment. The recorded signal was quantified and presented in the graph. Data are presented as mean ± SD with individual data (*n* = 3 per group). Statistical analysis was performed using *a t* test. *p < 0.05, **p < 0.01, ***p < 0.001, ****p < 0.0001. **e.** Hematological parameters following TriF_HS_-MMAE and crizotinib administration. Peripheral blood was collected t1 and t2 after intravenous administration of TriF_HS_-MMAE, oral administration of crizotinib (hydrochloride), or their combination. Data are presented as mean ± SD with individual data (*n* = 5 per group). Statistical analysis was performed using two-way ANOVA followed by Fisher’s LSD post hoc test. *p < 0.05, **p < 0.01, ***p < 0.001, ****p < 0.0001. **f.** Relative weights of the pancreas, spleen, liver, kidneys, and lungs were determined at t1 and t2 after treatment with TriF_HS_–MMAE. Relative organ weights were calculated as (organ weight [g] × 100%)/body weight [g]. Data are presented as mean ± SD with individual data (*n* = 5 per group). Statistical analysis was performed using two-way ANOVA followed by Fisher’s LSD post hoc test. *p < 0.05, **p < 0.01, ***p < 0.001, ****p < 0.0001.

We initially evaluated the time-dependent general toxicity of TriF_HS_-MMAE in a pilot study in order to establish conditions for the main experiments. For this purpose, TriF_HS_-MMAE was administered to mice at different times (0.5, 1, 6, 12, and 24 h) prior to euthanizing the animals. At the tested dose, TriF_HS_-MMAE did not affect mouse body weight or organ weights at any time point tested, indicating neither systemic toxicity nor procedure-related stress (Supplementary Figure 39). Histopathological analyses revealed that TriF_HS_-MMAE had no major impact on the examined tissues; only temporary alterations in the liver were observed (Supplementary Figure 40). Hematological analysis did not reveal marked abnormalities after TriF_HS_-MMAE administration, except for mild temporary changes in white blood cell counts and platelets (Supplementary Figure 41). We exploited the intrinsic fluorescence of TriF_HS_-MMAE to investigate its organ distribution. We observed rapid targeting of TriF_HS_-MMAE to the examined organs, with the strongest signals detected as early as 30 min after injection of the conjugate injection, followed by a gradual decline over time (Supplementary Figure 42). With Western blotting, we confirmed TriF_HS_-MMAE targeting to the pancreas and liver, two organs with high HSPG expression (Supplementary Figure 43). We also demonstrated that the liver and pancreas express different types of HSPGs (Supplementary Figure 44).

We next examined the effect of different exposure times to TriF_HS_-MMAE on the expression of genes encoding HSPGs in the pancreas and liver. No significant changes were detected in HSPG mRNA levels in the pancreas; however, in the liver, prolonged exposure to the conjugate resulted in decreased mRNA levels of several HSPGs, including syndecan-2 (SDC2), syndecan-4 (SDC4), glypican-1 (GPC1), and glypican-6 (GPC6) (Supplementary Figure 44). These data indicate that TriF_HS_-MMAE targets seem to be consistently available in the pancreas, whereas the liver may partially limit TriF_HS_-MMAE entry by repressing the expression of HSPGs. In summary, no overt systemic toxicity or hematological abnormalities were detected. These pilot experiments supported the selection of 1- and 3-h time points for subsequent experiments.

Subsequently, we explored a rational strategy to regulate TriF_HS_-MMAE endocytosis through modulation of cellular signaling, with the aim of reducing the conjugate’s toxicity in healthy organs *in vivo* (Figure 8a). For *in vivo* tests, from the highly specific inhibitors of TriF_HS_-MMAE/HSPG endocytosis via ADE that we identified, we selected crizotinib (hydrochloride), as it is a well-described compound used in the clinic for the treatment of metastatic nonsmall cell lung cancer (NSCLC), systemic anaplastic large cell lymphoma (ALCL), and inflammatory myofibroblastic tumor (IMT).^37^ Mice were pretreated with crizotinib (hydrochloride), followed by administration of TriF_HS_-MMAE for 1 or 3 h (Figure 8a). Following overnight fasting, body weights decreased uniformly after randomization and remained stable after drug administration across all experimental groups (Figure 8b).

After recognition of HSPGs at the cell surface, TriF_HS_-MMAE undergoes rapid internalization via ADE, leading to subsequent lysosomal trafficking and degradation of the conjugate (Figures 2 and 3). Pharmacological inhibition of TriF_HS_-MMAE cellular uptake *in vitro* using identified ADE modulators, including crizotinib (hydrochloride), resulted in pronounced accumulation of TriF_HS_-MMAE at the plasma membrane and concomitant impairment of its lysosomal degradation (Figure 5, Supplementary Figure 19). We determined whether a comparable effect occurs *in vivo* (Figure 8a). Organs were harvested from mice, and tissue-specific TriF_HS_-MMAE levels were assessed by measurements of the intrinsic TriF_HS_-MMAE fluorescence. Fluorescence analysis normalized to total protein and background-subtracted using tissues and plasma from PBS-treated control mice revealed marked, tissue-specific accumulation of TriF_HS_-MMAE (Figure 8c). In the combination group, plasma fluorescence was significantly increased at 1 h compared to TriF_HS_-MMAE monotherapy, whereas renal fluorescence was slightly reduced at this early time point (Figure 8c). At 3 h, increased fluorescence was detected in the liver and kidneys in the combination group, accompanied by declining plasma levels (Figure 8c). Pancreatic fluorescence remained low, yet a similar trend was detected after 1 h, with slightly increased TriF_HS_-MMAE fluorescence upon pretreatment with crizotinib (hydrochloride) in relation to TriF_HS_-MMAE standalone treatment (Figure 8c). Furthermore, with Western blotting, we evaluated changes in TriF_HS_-MMAE distribution to organs upon endocytic reprograming with specific ADE modulator crizotinib (hydrochloride). Mice pretreated with crizotinib (hydrochloride) exhibited increased accumulation of TriF_HS_-MMAE in the liver and pancreas compared with animals receiving the conjugate alone (Figure 8d). Since crizotinib (hydrochloride) has no impact on the interaction between TriF_HS_-MMAE and HSPGs *in vitro* (Supplementary Figure 45) or on HSPG binding at the cell surface (Supplementary Figure 30), but by blocking ADE causes accumulation of TriF_HS_-MMAE on the cell surface (Supplementary Figure 19), the enhanced levels of TriF_HS_-MMAE in organs of animals pretreated with crizotinib (hydrochloride) are most likely the result of its accumulation on the cell surface caused by blocked ADE-dependent lysosomal degradation. In agreement, at the earliest time point after the conjugate injection (1 h) virtually identical levels of TriF_HS_-MMAE were detected in the liver, independently of pretreatment with crizotinib (hydrochloride) (Figure 8c,d), and at that time the vast majority of TriF_HS_-MMAE fluorescent signal accumulated on the surface of hepatocytes (Supplementary Figure 46). These findings indicate altered systemic and tissue distribution kinetics of TriF_HS_-MMAE conjugate upon coadministration of crizotinib (hydrochloride).

Next, we assessed early tissue responses, organ weights, and hematological parameters. TriF_HS_-MMAE caused rapid depletion of leukocytes, lymphocytes, monocytes, and granulocytes, consistent with HSPG expression on these cells; erythrocytes, which express little to no HSPGs, remained unaffected (Figure 8e).^38^ Crizotinib (hydrochloride) alone had virtually no impact on white blood cells; however, priming animals with crizotinib (hydrochloride) prior to TriF_HS_-MMAE administration for 3 h almost completely prevented cytotoxicity in leukocytes and monocytes, and a similar trend was observed for the other cell types (Figure 8e). The other blood parameters remained largely unchanged (Supplementary Figure 47). Analysis of relative organ weights revealed early, tissue-specific changes. At 1 h, relative liver weight was increased in the TriF_HS_–MMAE group compared with those in the combination and crizotinib (hydrochloride) groups (Figure 8f). At 3 h, the relative weights of the pancreas and kidneys were significantly increased in the TriF_HS_-MMAE and crizotinib (hydrochloride) groups, which was not observed in the combination group (Figure 8f). The relative weight of the lungs remained virtually unaltered in all studied groups (Figure 8f). Plasma biochemical parameters were analyzed to assess hepatic and renal function following treatment. We detected transient, nonprogressive biochemical changes without evidence of acute hepatotoxicity or nephrotoxicity under the conditions tested (Supplementary Figure 48). Interestingly, at 3 h, crizotinib (hydrochloride) pretreatment resulted in a reduction of the elevated urea level observed in blood after TriF_HS_-MMAE or crizotinib (hydrochloride) standalone treatments (Supplementary Figure 48).

These results demonstrate that HSPG-specific TriF_HS_-MMAE targeting and toxicity toward healthy organs and blood cells can be effectively mitigated *in vivo* by priming animals with a selective blocker of HSPG-mediated ADE uptake, as identified in our study.

## Discussion

One of the most critical factors during the development of selective anticancer ADCs/PDCs is the selection of the target antigen.^39^ In current selection criteria, the target antigen should be expressed at a high level on the surface of cancer cells, have low expression in normal tissues, and display effective internalization.^40,41^ Such criteria exclude numerous cell-surface proteins that, due to differences in the kinetics and efficiency of endocytosis, ultimately could prove effective for ADCs/PDCs. Here, we demonstrate that, by rational manipulation of endocytic processes simultaneously from outside the cell (with a specific multivalent architecture of the PDC that triggers a highly efficient ADE internalization pathway) and from the cell interior (by adjusting specific endocytic signaling), it is possible to modulate PDC delivery both *in vitro* (Figure 7a) and *in vivo* (Figure 8a).

As a model target, we intentionally selected widely expressed HSPGs, which play critical roles in the initiation, progression, and invasion of distinct cancer types, especially in highly lethal pancreatic cancer.^13,17^ So far, only two PDCs targeting HSPGs have been developed: GPC1-ADC (MMAE) and GPC1-ADC (MMAF), which are of conventional architecture, composed of the same anti-GPC1 targeting proteinaceous component conjugated to MMAE or MMAF, respectively.^42,43^ Although demonstrating inhibition of pancreatic tumor growth, none of these ADCs have been approved for pancreatic cancer treatment so far. Targeting the proteinaceous portion of GPC1 makes such conjugates highly vulnerable to the development of drug resistance through suppression of GPC1 expression and compensation of its function by other HS-bearing proteoglycans.^44^ To overcome this limitation, we developed a novel multivalent PDC with a unique molecular architecture and functionalities that specifically targets HS chains of HSPGs, TriF_HS_-MMAE.

Endocytosis is a critical step in an ADC’s mechanism of action.^40^ Recent reports from us and others have demonstrated that clustering of cell-surface receptors with multivalent ligands induces a rapid and highly efficient CIE internalization that occurs via the recently identified ADE pathway, which morphologically resembles macropinocytosis.^6,7,9^ Besides the beneficial impact on endocytosis, the multivalency of drug carriers ensures a higher affinity for receptors, increasing the precision of conjugate delivery into tumor cells and implying the superiority of multivalent ligands over conventional bivalent mAbs.^10^ Therefore, using GFP polygons (GFPp) as an oligomerization scaffold, we developed, to our knowledge, the first inherently fluorescent multivalent PDC, TriF_HS_-MMAE, capable of directly clustering HSPG on the cell surface. Although GFP can be immunogenic, our *in vivo* experiments did not reveal an acute immune response to GFPp.^45,46^ Even if an immune response against GFPp were to arise during later stages of TriF_HS_-MMAE development, several established strategies could be employed to mitigate immunogenicity, such as sequence humanization or chemical modification.^47,48^ Alternatively, to achieve multivalency, GFPp can be replaced by other nonimmunogenic oligomerization scaffolds, including IgG-derived frameworks or coiled-coil motifs that are naturally abundant in human proteins.^49,50^

We demonstrate that TriF_HS_-MMAE-triggered HSPG cross-linking induces ultrafast, clathrin-independent, dynamin-2-dependent ADE of TriF_HS_-MMAE/GPC4 complex in pancreatic cells, effectively delivering TriF_HS_-MMAE/HSPG via endosomes to lysosomal compartments for degradation and intracellular drug release (Figure 7a). With these data, we have demonstrated that it is possible to force endocytosis of HSPG from outside the cell by applying a specific PDC architecture. To our knowledge, our studies constitute the first detailed report on HSPG endocytosis via ADE.

Although TriF_HS_-MMAE demonstrated higher selectivity for all tested pancreatic cancer cell lines than for healthy pancreatic cells, it also displays considerably high toxicity toward healthy cells, which was expected due to the high abundance of HSPG on healthy cells.^51^ These data highlight the need for the development of additional modalities that, in combination with multivalency-dependent ADE induction, will increase the selectivity of HSPG-specific cytotoxic conjugates. Endocytosis and signaling are intertwined processes, and their tight balance is critical for cell homeostasis.^52,53^ Signaling pathways can modulate the selectivity, efficiency, and kinetics of endocytic events, thus we decided to focus on kinase activity as a means to improve the selectivity of TriF_HS_-MMAE targeting (Figure 7a). Our idea was strengthened by the fact that numerous kinase inhibitors are approved drugs, including antitumor therapeutics,^54^ which could simplify their use in combination with the PDC. Yet the signaling pathways that control the endocytosis of HSPG in pancreatic cells were virtually unknown. Using HCS screening with a near-kinome-wide library of kinase inhibitors, we identified general HSPG endocytic regulators and compounds that act as highly specific ADE modulators. Among blockers of HSPG ADE, we identified a few compounds that act on Rac1-downstream kinases, such as Cdc42 (4 different inhibitors), CDK7, and CDK9, which is in line with the reported involvement of Rac1 in the ADE of HER2.^6^

Oncogenic transformation is associated with imbalanced cellular signaling.^55,56^ Thus, we hypothesized that differences in endocytic signaling between normal and pancreatic cancer cells could be used to modulate the selectivity of TriF_HS_ endocytosis. We intended to prime both normal and malignant pancreatic cells with the same kinase inhibitor in order to downregulate TriF_HS_-MMAE uptake via ADE into healthy cells and either have no effect or preferentially promote internalization of TriF_HS_-MMAE into malignant cells (Figure 7a). In the second HCS screen, we identified nine inhibitors that either had no effect on the endocytosis of TriF_HS_-MMAE by cancer cells or even promoted TriF_HS_-MMAE uptake in malignant cells. With these data, we demonstrated that it is possible to control the highly specific endocytosis of TriF_HS_-MMAE from within the cell via cellular signaling. We showed that it is feasible to prime normal and pancreatic cancer cells with the same kinase inhibitor in order to elicit a differential effect on ADE endocytosis: blocking TriF_HS_-MMAE uptake by normal cells while promoting TriF_HS_-MMAE internalization by malignant cells, thereby improving the selectivity of PDC toxicity (Figure 7).

## Conclusion

Typically, PDC targets are not exclusively expressed in tumors; their low-level presence in healthy tissues underlies the side effects of PDCs. Because of its very high HS selectivity, TriF_HS_-MMAE targets several organs, as they are rich in HSPGs. To our knowledge, we demonstrated for the first time *in vivo* that priming animals with highly specific ADE modulators identified by us in *in vitro* HCS screens enables the reduction of TriF_HS_-MMAE mistargeting to healthy organs and cells, thereby diminishing the conjugate’s side effects (Figure 8). Since kinase inhibitors are often well-studied compounds and approved drugs, their application as specific endocytic modulators of PDCs may accelerate the development of novel multimodal treatment strategies, which have recently gained momentum in the ADC/PDC field.^57,58^ Our study provides a proof-of-principle demonstration of the proposed strategy and establishes its feasibility under the tested conditions. However, the translation of therapies based on this approach into clinical practice will require extensive further investigation, including rigorous evaluation in subsequent stages of preclinical development and ultimately in clinical trials.

## Experimental Section

### Antibodies and Reagents

Anti-DNM2 primary antibody (#4390824), anti-GPC4 polyclonal antibody (#PA5-97801), secondary fluorescent antibody DyLight 650 (#84546), secondary fluorescent antibody Alexa Fluor 594 (#a11037) and DyLight 488 (#46402) were from Thermo Fisher Scientific (Waltham, MA, USA). The primary antibody directed against CHC1 (#610499) was from BD Transduction Laboratories (Bergen, NJ, USA). Anti-EEA1 antibody (1E11E4) (#68065-1-IG150UL) was from Proteintech (Rosemont, IL, USA). Anti-LAMP1 antibody (#ab24170) and secondary fluorescent antibody Alexa Fluor 594 (#ab150120) were from Abcam (Waltham, MA, USA). Anti-aggrecan antibody (chondroitin sulfate antibody) (#C8035) was purchased from Sigma-Aldrich (Saint Louis, MO, USA). Anti-heparan sulfate biotinylated antibody (clone F58–10E4) (#370255-B-100-HL) was from Amsbio (Abingdon, UK). Secondary fluorescent antibody DyLight 650 (#84546) was from Thermo Fisher Scientific. Anti-FGF1 rabbit polyclonal antisera (OMA1) were used as described previously.^59^ Anti-eGFP-tag rabbit polyclonal antibody (#50430-2-AP) was from Proteintech Group, Inc. (Rosemont, IL, USA). Anti-beta-tubulin antibody (#sc-55529) was from from Santa Cruz (Dallas, TX, USA). Anti-MMAE mouse monoclonal primary antibody (#MME-M5252) was purchased from ACROBiosystems (Newark, DE, USA). Anti phospho-FGFR-1 (Tyr653/Tyr654) antibody (#06-1433) and anti-γ-tubulin (#T6557) were purchased from Sigma-Aldrich (Saint Louis, MO, USA). Anti-Phospho-p44/42 MAPK (Erk1/2) (Thr202/Tyr204) Antibody (#9101) was purchased from Cell Signaling (Leiden, The Netherlands). HRP-conjugated secondary antibody (#115-035-003) was obtained from Jackson Immuno-Research Laboratories (Cambridge, UK). GPC4-Fc chimera protein (#9195-GP-050) was purchased from R&D Systems, Inc. (Minneapolis, MN, USA). Heparin sodium salt from porcine intestinal mucosa (#9041-08-1), chondroitin sulfate A sodium salt from bovine trachea (#C9819), chondroitin sulfate B sodium salt from porcine intestinal mucosa (#C3788), Heparinase III from *Flavobacterium heparinum* (#H8891), and Chondroitinase ABC from *Proteus vulgaris* (#C3667) were purchased from Sigma-Aldrich (Saint Louis, MO, USA). Surfen dihydrochloride (HY-122704A) and crizotinib (hydrochloride) (HY-50878A) were from MedChemExpress (Monmouth Junction, NJ, USA). PNGase F (#P0704S) was purchased from New England Biolabs (Ipswich, MA, USA). Heparin Sepharose 6 Fast Flow affinity resin (#17099825) and the chromatographic column HiTrap Heparin HP (#17040701) were from GE Healthcare (Chicago, IL, USA).

Reagents used for solid-phase peptide synthesis were as follows: amino acids Fmoc-Gly-OH, Fmoc-l-Ser­(tBu)-OH, and Fmoc-O2Oc-O2Oc–OH; DIC/Oxyma Pure, EDT (ethane-1,2-dithiol), piperidine, TIS (triisopropylsilane), DIPEA (*N,N*-diisopropylethylamine), DMF (*N,N*-dimethylformamide), DCM (dichloromethane), and TFA (trifluoroacetic acid) were purchased from Iris Biotech GmbH (Marktredwitz, Germany). HPLC-grade acetonitrile and Et_2_O (diethyl ether) were from Avantor (Gliwice, Poland). Fmoc-Cys-RAM TentaGel was from Rapp Polymere GmbH (Tübingen, Germany). The cytotoxic agents MMAE (monomethyl auristatin E) and mc-vc-PAB-MMAE (#HY-15575) were from MedChemExpress (Monmouth Junction, NJ, USA).

The NucBlue Reagent (Hoechst 33342) (#R37605), DyLight 550 NHS Ester (#62263), and HCS CellMask Stain Deep Red (#32721) were from Thermo Fisher Scientific. Dextrans (Alexa Fluor 555 conjugate, 10,000 MW; #D34679) and Albumin from Bovine Serum (Alexa Fluor 555 conjugate; #A34786) were purchased from Thermo Fisher Scientific (Waltham, MA, USA). Fluorescein (FITC)-human transferrin (#009-090-050) was obtained from Jackson ImmunoResearch Laboratories (Cambridge, UK). Kinase Inhibitor Library (#HY-L009) was from MedChemExpress (Monmouth Junction, NJ, USA). DMSO (sc-358801) was from from Santa Cruz (Dallas, TX, USA). Pierce High Capacity Endotoxin Removal Spin Columns (#88276) and Pierce BCA Protein Assay Kit (#23227) were from Thermo Fisher Scientific (Waltham, MA, USA). cOmplete, EDTA-free, Protease Inhibitor Cocktail was from Sigma-Aldrich (Saint Louis, MO, USA). All other reagents were obtained from Sigma-Aldrich (Saint Louis, MO, USA).

### Cells

The human pancreatic cell line hTERT-HPNE and human pancreatic cancer cell lines HPAF-II, Capan 2, PANC-10.05, and CFPac-I were obtained from the American Type Culture Collection (ATCC) (Manassas, VA, USA). The hTERT-HPNE cell line was cultured in 75% Dulbecco’s Modified Eagle’s Medium, high glucose (Gibco, New York, USA), supplemented with 25% Medium M3 Base (Incell Corp., San Antonio, Texas, USA), 5% fetal bovine serum (FBS) (ThermoFisher Scientific, Waltham, MA, USA), an antibiotic mix (100 U/mL penicillin and 100 μg/mL streptomycin) (Thermo Fisher Scientific, Waltham, MA, USA), and 10 ng/mL human recombinant EGF (Manassas, VA, USA). HPAF-II and Capan 2 cell lines were cultivated in DMEM (Biowest, Nuaille, France) supplemented with 10% fetal bovine serum (Thermo Fisher Scientific, Waltham, MA, USA) and an antibiotic mix (100 U/mL penicillin and 100 μg/mL streptomycin). The human pancreatic adenocarcinoma cell line PANC-10.05 was cultivated in RPMI medium (#R1145) from Sigma-Aldrich (St. Louis, MO, USA), supplemented with 15% fetal bovine serum (FBS) (ThermoFisher Scientific, Waltham, MA, USA), an antibiotic mix (100 U/mL penicillin and 100 μg/mL streptomycin) (Thermo Fisher Scientific, Waltham, MA, USA), and 10 U/ml human recombinant insulin (#91077) from Sigma-Aldrich (St. Louis, MO, USA). The human pancreatic adenocarcinoma cell line CFPac-I was cultivated in IMDM medium (#30-2008) from ATCC (Manassas, VA, USA), supplemented with 5% fetal bovine serum (FBS) (ThermoFisher Scientific, Waltham, MA, USA) and an antibiotic mix (100 U/mL penicillin and 100 μg/mL streptomycin) (Thermo Fisher Scientific, Waltham, MA, USA). All cell lines were cultured in a 5% CO_2_ atm at 37 °C and were seeded onto tissue culture plates 1 day prior to the start of the experiments.

Mouse embryo fibroblast cells (NIH3T3) were purchased from the American Type Culture Collection (ATCC, Manassas, VA, USA). NIH3T3 cells were cultured in DMEM (Thermo Fisher Scientific, Waltham, MA, USA) supplemented with 10% fetal bovine serum (Thermo Fisher Scientific, Waltham, MA, USA) and antibiotics (100 U/mL penicillin and 100 μg/mL streptomycin). The murine pro B cell line (BaF3) was a kind gift from Dr. David Ornitz of the Department of Developmental Biology, Washington University School of Medicine.^60^ The cells were cultured in RPMI-1640 medium (Thermo Fisher Scientific) supplemented with 10% newborn bovine calf serum (Thermo Fisher Scientific), antibiotics (100 U/mL penicillin and 100 μg/mL streptomycin), β-mercaptoethanol (50 nM), and mouse interleukin-3 (IL-3, PeproTech). Human hepatocytes (HepaRG; #HPRGC10) were purchased from Thermo Fisher Scientific (Waltham, MA, USA). HepaRG cells were cultured in William’s E Medium, GlutaMAX Supplement (#32551020), supplemented with HepaRG Thaw, Plate & General Purpose Medium Supplement (5x) (Thermo Fisher Scientific, Waltham, MA, USA, #HPRG770). Human endothelial cells (HUVECs) were purchased from the American Type Culture Collection (ATCC, Manassas, VA, USA). HUVECs were cultured in F-12K Medium (ATCC, Manassas, VA, USA, #30-2004) supplemented with 0.1 mg/mL heparin (Sigma-Aldrich, Saint Louis, MO, USA, #9041-08-1), 10% fetal bovine serum (ThermoFisher Scientific, Waltham, MA, USA), 30 μg/mL Endothelial Cell Growth Supplement (ECGS) (Fisher Scientific, Waltham, MA, USA, #CB-40006B), and antibiotics (100 U/mL penicillin and 100 μg/mL streptomycin). All cell lines were seeded onto tissue culture plates 1 day prior to the start of the experiments.

### Recombinant Proteins

The genetic constructs designed for the production of FGF1_HS_ and GFPp-FGF1_HS_ were prepared via gene synthesis (Gene Universal, Newark, DE, USA). Amino acid sequences of the produced FGF1_HS_ and GFPp-FGF1_HS_ are listed in Supplementary Data 1. The truncated wild-type FGF1 (Met-Ala-FGF1^21–154^ named FGF1_WT_) cloned into the pET-3c vector was used as a control.^59^ Individual colonies of transformed *Escherichia coli* (*E. coli*) BL21­(DE3)-RIL strain (Agilent Technologies, Santa Clara, CA, USA) were grown in LB medium with 100 μg/mL ampicillin and 30 μg/mL chloramphenicol for 16 h at 37 °C, 150 rpm. Overnight cultures were then diluted 1:100 in LB medium with 100 μg/mL ampicillin and 30 μg/mL chloramphenicol and cultivated at 37 °C, 150 rpm to OD_600_ = 0.8. Protein expression was induced by the addition of 1 mM IPTG, followed by incubation of the bacteria at 25 °C for 18 h. After overnight incubation, the bacterial cultures were centrifuged (4 °C, 5000 × *g*, 10 min), and the collected bacterial pellet was resuspended in 0.5 M NaCl (in 25 mM HEPES, pH 7.6) and sonicated. After sonication, lysates were centrifuged for 40 min at 14,000 × *g* and 4 °C. The collected supernatant was incubated with Heparin Sepharose 6 Fast Flow resin (GE Healthcare, Piscataway, NJ, USA) for 2 h or overnight at 4 °C. After incubation, the next steps of purification were performed at room temperature. First, the resin was washed with 0.5 M NaCl (25 mM HEPES, pH 7.6), and the protein was eluted from the column with 2 M NaCl (in 25 mM HEPES, pH 7.6). Various oligomeric forms (from monomers to tetramers) were isolated via elution from the Heparin Sepharose 6 Fast Flow column with a NaCl gradient or isocratic flow (in 25 mM HEPES, pH 7.6) in the range of 0.5 to 2 M using an NGC chromatography system (Bio-Rad, Hercules, CA, USA). The extracellular region of FGFR1 fused to the Fc fragment of human IgG1, and the Fc fragment of human IgG1, were produced as described previously.^61^ The evolved sortase A (eSortA) pentamutant with improved kinetics and activity was produced in *E. coli* as described earlier.^62,63^ The purity and identity of the obtained proteins were confirmed by SDS-PAGE, Western blotting, and mass spectrometry. The native conformations of the proteins were confirmed by circular dichroism spectroscopy.

### BLI Measurements

Binding analysis of FGF1_WT_, FGF1_HS_, BiF_HS_, TriF_HS_, TetraF_HS_, TriF_HS_-MMAE to FGFR1 Fc or glypican-4 Fc chimera protein was performed using biolayer interferometry (BLI) with the ForteBio Octet K2 (Pall ForteBio, San Jose, CA, USA). FGFR1 Fc or glypican-4 Fc chimera protein and the Fc were immobilized on Protein-A sensors (loading step in a 150 nM solution for 600 s) in a pairwise manner (the studied protein and the Fc as a reference sensor), and the sensors were subsequently incubated with the studied proteins (300 nM) for 450 s, followed by a 450 s dissociation step. For binding analysis of TriF_HS_-MMAE to glypican-4 Fc chimera protein in the presence or absence of crizotinib (hydrochloride), 800 nM TriF_HS_-MMAE was used in the association step (600 s), and a second sensor was incubated under the same conditions but with a 10-fold molar excess of crizotinib (hydrochloride) (8 μM), and dissociation in both cases was monitored for 600 s. For binding analysis of TriF_HS_ to HS, CS, and DS, NTA biosensors were used for immobilization of His-tagged TriF_HS_ (20 μg/mL, 450 s), then the sensors were incubated with HS, CS, and DS (3 μg/mL) for 450 s, followed by a 450 s dissociation step. All experiments were conducted at 25 °C with shaking at 1000 rpm in phosphate buffered saline running buffer. Each measured signal was control (the Fc) and baseline-subtracted.

### FGFR1 Activation

To analyze the impact of FGF1_WT_ and FGF1_HS_ on the activation of FGFR1 and initiation of receptor-downstream signaling cascades, serum-starved NIH3T3 cells (12-well plates, 100,000 cells/well) were treated with increasing concentrations of FGF1_WT_ (1, 2, 5, 10, and 20 ng/mL) or FGF1_HS_ (2, 20, 200, and 2000 ng/mL) for 15 min at 37 °C. Cells were lysed in Laemmli buffer and subjected to SDS-PAGE and Western blotting.

### Western Blotting

Purified proteins, or proteins extracted from cells using a lysis buffer (containing 8% SDS and 2% β-ME) and subsequent sonication and heating, were separated by SDS-PAGE and transferred onto a PVDF membrane by electroblotting. The membrane was probed with a primary antibody that specifically recognizes the protein of interest, followed by washing to remove unbound primary antibody. To detect the protein–antibody complex, an HRP-conjugated secondary antibody was incubated with the membrane. Finally, the membrane was washed, incubated with an HRP-substrate, and the proteins were visualized using ChemiDoc (BioRad, Hercules, CA, USA).

### Native PAGE

Separation of proteins under nondenaturing conditions was performed using Native PAGE.^64^ Proteins (5 μg) were separated on 10% native gels using Tris-glycine running buffer (25 mM Tris, 192 mM glycine, pH 8.3). Native gels were run on ice at 100 V, and after separation, the gels were imaged under UV light.

### Size-Exclusion Chromatography

The oligomeric state of the purified proteins was assessed by size-exclusion chromatography (SEC) using an ÄKTA Explorer FPLC system with a HiLoad Superdex 200 10/300 GL column. The oligomers at a concentration of 1 mg/mL were injected onto the column and run at a flow rate of 1 mL/min in PBS buffer. The absorbance spectra were monitored at 280 nm. Molecular weight standards containing BPTI, cytochrome c, carbonic anhydrase, human serum albumin, α-lactoglobulin, chymotrypsinogen A, and ovalbumin (Sigma-Aldrich, St. Louis, MO, USA) were used to generate a standard curve from which the average molecular weights of individual oligomers were calculated.

### Fluorescence Microscopy

Binding analysis of oligomeric variants BiF_HS_, TriF_HS_, and TetraF_HS_ to HSPGs was performed using the hTERT-HPNE cell line. Cells (7000 cells per well) were incubated with the oligomers at a concentration of 585 nM for 15 min on ice. To analyze the internalization of BiF_HS_, TriF_HS_ and TetraF_HS_ oligomers by pancreatic cells, hTERT-HPNE cells were incubated with 585 nM of each oligomer in serum-free medium at 37 °C for 30 min. After this time, the internalization was stopped by cooling the cells on ice. To analyze TriF_HS_ internalization by cells lacking cell surface HS (BaF3 cell line) compared with HS-expressing cells (hTERT-HPNE cell line), BaF3 and hTERT-HPNE cells were incubated with 585 nM of TriF_HS_ and NucBlue Live in serum-free medium at 37 °C for 30 min. To analyze the mechanism of TriF_HS_ internalization into normal pancreatic cells, hTERT-HPNE cells were preincubated with heparinase III (0.1 U/ml), sodium chlorate (50 mM), or surfen (0.75 mM) at 37 °C for 2 h, overnight, and 2 h, respectively. In the experiments involving the addition of glypican-4 (20 μg/mL), heparin (20 μg/mL), chondroitin sulfate (20 μg/mL), or dermatan sulfate (20 μg/mL), the sulfates or the proteoglycan were added simultaneously with TriF_HS_. After this time, 585 nM of TriF_HS_ was added, and cells were incubated at 37 °C for 30 min. Internalization was stopped by cooling the cells on ice. hTERT-HPNE cells were preincubated with chondroitinase ABC (100 mU) at 37 °C for 4 h. Subsequently, 585 nM of TriF_HS_ was added, and cells were incubated at 37 °C for 30 min. To analyze whether TriF_HS_ internalization into normal pancreatic cells depends on N-glycans, hTERT-HPNE cells were preincubated with PNGase F at 37 °C for 4 h. After this time, 585 nM of TriF_HS_ or Gal3 (10 μg/mL) was added, and cells were incubated at 37 °C for 30 min. To analyze the colocalization of TriF_HS_ with model endocytosis cargoes, hTERT-HPNE cells were incubated with TriF_HS_ and albumin (0.2 mg/mL), dextran (0.4 mg/mL), transferrin (0.4 mg/mL), or galectin-3 (10 μg/mL), respectively, in serum-free medium at 37 °C for 30 min. To analyze the changes in HSPG endocytosis caused by nine model inhibitors, hTERT-HPNE cells or HPAF-II cells were treated with the inhibitors (4 μM) in serum-free medium at 37 °C for 30 min. GPC4 antibody or HS antibody immunofluorescence staining was performed as described above. To analyze the colocalization of TriF_HS_ with GPC4 antibody or HS antibody after treatment with model inhibitors, hTERT-HPNE cells or HPAF-II cells were treated with the inhibitors (4 μM) in serum-free medium at 37 °C for 30 min, followed by staining with GPC4 or HS antibodies. To analyze the changes in the internalization process of model cargoes (transferrin, albumin, dextrans, galectin-3 conjugated with Alexa Fluor 555) after treatment with nine model inhibitors, hTERT-HPNE cells were pretreated with the inhibitors (4 μM) in serum-free medium at 37 °C for 30 min. After this time, model cargoes were added to cells, and cells were incubated at 37 °C for 30 min. To analyze changes in tubulin morphology following treatment with crizotinib (hydrochloride), TriF_HS_, MMAE, or their combination, hTERT-HPNE cells were treated with crizotinib (hydrochloride) (4 μM), TriF_HS_-MMAE (at a concentration equal EC_50_ for hTERT-HPNE), MMAE (at a concentration equal to TriF_HS_-MMAE), or their combination at 37 °C for 24 h and subsequently fixed. β-tubulin (Pheno 594) was detected by immunolabeling. Cells were washed with PBS and fixed in 4% paraformaldehyde solution. Nuclei were stained with NucBlue Live dye, and cells were permeabilized with 0.1% Triton in PBS and stained with HCS CellMask Deep Red Stain, followed by blocking with 3% BSA and incubation with antibodies for immunofluorescence. Fixed and labeled cells were analyzed with quantitative confocal microscopy using the Opera Phenix Plus High-Content Screening System (PerkinElmer, Waltham, MA, USA). Measurements were carried out in confocal mode using a 63 × water-immersion, NA 1.15 objective with 2× binning and two peaks autofocus. 37 fields per well were imaged, with 8–14 Z-stacks per field at 0.5 μm intervals to ensure comprehensive imaging of the cells. 2160 × 2160 px camera ROI was used to capture the images. The Harmony High-Content Imaging and Analysis Software (version 5.1 and 5.2; PerkinElmer, Waltham, MA, USA) was used for image acquisition and analysis. The number of cells and the cell boundaries were determined using DAPI and CellMask Deep Red, respectively. The intensity of the fluorescent signal in the Alexa 488 channel was measured and compared between cells. Images were assembled in Adobe Illustrator with only linear adjustments to contrast and brightness.

To study the kinetics of internalization of TriF_HS_ and FGF1_HS_, a live confocal microscopy assay was performed at 37 °C, 5% CO_2_, and controlled humidity using the Opera Phenix Plus platform with a pipettor. hTERT-HPNE cells (7000 cells per well) were pretreated with NucBlue Live to visualize nuclei. The appropriate concentration of TriF_HS_ or FGF1_HS_ was then added directly to the wells using the pipettor. Images were captured every 1 s or 273 ms using confocal mode with a 63× water objective (NA 1.15), 2× binning, and two-peak autofocus. The 2160 × 2160 px camera ROI was used to capture the images. Images were assembled in Fiji and Illustrator (Adobe) with only linear contrast and brightness adjustments. Measurements were carried out using confocal mode as described above.

To analyze the colocalization of TriF_HS_ with EEA1 (early endosome antibody 1), LAMP-1, or GPC4, immunofluorescence staining was performed. hTERT-HPNE cells were incubated with 585 nM TriF_HS_ in serum-free medium at 37 °C for 30 min or at 37 °C for 6 h for experiments on the colocalization of TriF_HS_ with lysosomes. Afterward, internalization was stopped by cooling the cells on ice. Cells were subsequently washed with PBS and fixed in 4% paraformaldehyde solution. Nuclei were stained with NucBlue Live dye, and cells were permeabilized with 0.1% Triton in PBS. Then, cells were washed with PBS, blocked with 2% BSA, and incubated with antibody against EEA1, LAMP-1, or GPC4 at 4 °C overnight. Next, cells were washed with PBS and incubated with an appropriate secondary fluorescent antibody (goat antirabbit IgG secondary antibody conjugated to Alexa Fluor 594 or Alexa Fluor 650) for 1 h at RT. Fixed and labeled cells were analyzed by quantitative confocal microscopy using the Opera Phenix Plus High-Content Screening System (PerkinElmer, Waltham, MA, USA) or the STELLARIS Confocal Microscope Platform (Leica, Wetzlar, DE). Measurements were carried out using confocal mode with an 86 × water objective. The Leica LAS X software was used for image acquisition and analysis. Images were assembled in Fiji and Illustrator (Adobe), with only linear contrast and brightness adjustments.

### FPLC Elution Profiles

MonoF_HS_, BiF_HS_, TriF_HS_, and TetraF_HS_ affinities for heparin were evaluated using a HiTrap Heparin-Sepharose column (GE Healthcare) and a linear NaCl gradient (in 25 mM HEPES, pH 7.4) over a range from 0.5 to 2 M at room temperature, using an NGC chromatography system (Bio-Rad, Hercules, CA, USA).

### Analysis of Protein Stability

To analyze the stability of TriF_HS_, the protein (20 μg) was incubated in 10-fold-diluted human serum (Sigma-Aldrich, St. Louis, MO, USA) at 37 °C for 96 h. At distinct time points (0, 24, 48, 72, and 96 h), samples were taken, and the samples were analyzed by native PAGE , SDS-PAGE, and fluorescence spectroscopy.

### Fluorescence Measurements

Fluorescence spectra were measured in phosphate buffer (25 mM H_3_PO_4_, pH 7.3) at 20 °C using an FP-8500 spectrofluorometer (Jasco, Japan), with excitation at 488 nm and emission collected in the 500–650 nm range. All measurements were carried out at a protein concentration of 0.2 or 0.5 μM using a 1-mm quartz cuvette.

### Circular Dichroism (CD) Measurements

CD spectra were recorded in the wavelength range 200–260 nm in phosphate buffer (25 mM H_3_PO_4_, pH 7.3) at 20 °C on a Jasco J-1500 spectropolarimeter. Spectra were acquired at a protein concentration of 0.5 μM using a 1 mm quartz cuvette.

For thermal denaturation experiments, ellipticity changes at 228 nm were measured at a scan rate of 1 °C/min from 20 to 80 °C using 0.5 μM protein samples in phosphate buffer (25 mM H_3_PO_4_, pH 7.3). The data were collected using a 10 mm quartz cuvette on a Jasco J-1500 spectropolarimeter and analyzed using OriginPro 2026 software (Northampton, MA) for *T*_m_ estimation using sigmoidal growth model fitting.

### Turbidity Measurements

The turbidity of TriF_HS_ samples was measured using a Prometheus Panta instrument (NanoTemper Technologies GmbH, Munich, Germany). An isothermal turbidity scan of 585 nM TriF_HS_ in PBS was recorded for 24 h at 37 °C, in the presence or absence of 4 μM selected inhibitors (4SC-203, prexasertib dimesylate).

### Dynamic Light Scattering

The oligomeric state of the purified proteins (FGF1_HS_, BiF_HS_, TriF_HS_, TetraF_HS_) was analyzed by dynamic light scattering (DLS) using a Prometheus Panta instrument (NanoTemper Technologies GmbH, Munich, Germany). Measurements were performed at a protein concentration of 10 μM in PBS. Samples were equilibrated to room temperature prior to acquisition. To assess HSPG clustering induced by TriF_HS_ *in vitro*, DLS was performed using recombinant GPC4. GPC4, TriF_HS_, and their mixture were prepared in PBS at a final concentration of 10 μM for each protein. For the complex formation assay, proteins were mixed at a 1:1 molar ratio and incubated for 15 min at room temperature before measurement. DLS analyses were performed for GPC4 alone, TriF_HS_ alone, and the preincubated mixture under identical instrument settings. Hydrodynamic size distributions were recorded as intensity-weighted profiles using the Prometheus Panta software.

### siRNA Transfection

Cells were transfected with siRNA against endocytic proteins using DharmaFECT (Horizon, Cambridge, UK) according to the manufacturer’s instructions. hTERT-HPNE cells were treated with siRNA against clathrin heavy chain (CLTC (#4390824, Thermo Fisher Scientific, Waltham, MA, USA) and dynamin-2 (DNM2 (#4390824, Thermo Fisher Scientific, Waltham, MA, USA) at a concentration of 50 or 100 nM for 24 h. Control cells were transfected with 100 nM nontargeting siRNA (#D-001810-01-50, Horizon, Cambridge, UK). After 24 h, the transfection medium was replaced with complete medium, and the incubation was continued for another 24 h. Then, cells were seeded on microscopy plates (7000 cells per well) in complete medium and left to attach overnight. The next day, TriF_HS_ was added to the cells in serum-free medium at 37 °C for 30 min. After this time, internalization was stopped by cooling the cells on ice. Cells were subsequently washed with PBS and fixed in 4% paraformaldehyde solution. Nuclei were stained with NucBlue Live dye, and cells were permeabilized with 0.1% Triton in PBS and stained with HCS CellMask Deep Red Stain. Cells were analyzed using confocal microscopy. The effectiveness of CLTC and DNM2 downregulation was confirmed by Western blotting.

### Synthesis of GGGS-(O2Oc)_2_-C­(vcMMAE)-NH_2_ Peptide

The GGGS-(O2Oc)_2_-C-NH_2_ peptide was synthesized utilizing the Fmoc SPPS strategy on Fmoc-Cys-RAM TentaGel resin (Rapp Polymere, Germany). Amino acid coupling using DIC with Oxyma Pure was performed on a microwave peptide synthesizer (CEM Liberty Blue 2.0, USA) using standard microwave power and coupling times. The finished peptide was cleaved from the resin using a mixture of TFA/TIPS/1,2-EDT/thioanisole/thiophenol/H_2_O (90/2/2/2/2/2). Next, the majority of TFA was evaporated using a stream of nitrogen. Finally, the peptide was precipitated with cold diethyl ether (Et_2_O) and centrifuged. Several Et_2_O washing and centrifugation cycles were performed to ensure that the pellet contained a minimal amount of the scavenging reagents. The peptide purification was performed on a Waters 1525 HPLC system using a Phenomenex Gemini-NX C18 column, utilizing a gradient of acetonitrile in 0.1% TFA. The identity of the peptide was confirmed by mass spectrometry. The pooled peptide fractions were frozen and lyophilized. GGGS-(O2Oc)_2_-C-NH_2_ (36.9 mg, 55.2 μmol) and maleimide-vcMMAE (MC-vc-PAB-MMAE, 21.8 mg, 16.6 μmol, 0.3 equiv), both dissolved in DMAc, were mixed (418 μL), followed by the addition of DIPEA (28.8 μL, 165.6 μmol, 3 equiv). The reaction was conducted at RT for 1 h. The solvent was then removed under vacuum, and the GGGS-(O2Oc)_2_-C­(vcMMAE)-NH_2_ was purified by RP-HPLC and lyophilized. The identity of the product was confirmed by mass spectrometry.

### Conjugation of the TriF_HS_ with MMAE via Sortase A-Mediated Ligation

Purified engineered trimeric TriF_HS_ containing the C-terminal LPETGG sequence was transferred to the sortase A reaction buffer (25 mM HEPES, pH 7.6, 154 mM NaCl, 5 mM CaCl2, 2 mM TCEP) using Zeba Spin Desalting Columns (Thermo Fisher Scientific, Waltham, MA, USA). The final concentration of protein used in the conjugation reaction was 250 μg/mL. The GGGS-(O2Oc)_2_-C­(vcMMAE)-NH_2_ peptide was added to the protein solution to achieve a final concentration of 891 μM. Then sortase A was added to a final concentration of 5 μM and the mixture was incubated overnight at 16 °C with 550 rpm end-over-end rotation. After incubation, the protein was purified by affinity chromatography using a HiTrap heparin column (GE Healthcare, Piscataway, NJ, USA). The resin was washed with washing buffer containing 25 mM HEPES at pH 8.0, 0.2 M NaCl, 1 mM EDTA, and 1 mM DTT to remove unconjugated peptide and sortase A, and then TriF_HS_-MMAE was eluted with elution buffer containing 25 mM HEPES at pH 8.0, 2 M NaCl, 1 mM EDTA, and 1 mM DTT. The purity of the obtained TriF_HS_-MMAE conjugate was assessed by size-exclusion chromatography using a ProteoSEC 6-600 HR column. Chromatographic peak areas were integrated, and the purity was calculated as the percentage of the main TriF_HS_-MMAE peak area relative to the total integrated peak area, yielding a purity of 92.6%. The identity of the conjugate was confirmed by mass spectrometry (calculated mass: 45,085.70 Da; observed mass: 45,085.20 Da).

For *in vivo* mouse studies, the TriF_HS_-MMAE conjugate was purified of residual endotoxins using Pierce High Capacity Endotoxin Removal Spin Columns (Thermo Fisher Scientific, catalog no. 88276) according to the manufacturer’s instructions. Following endotoxin removal, the conjugate was concentrated to 2.39 mg/mL, sterile-filtered through a 0.22 μm syringe filter, and aliquoted for animal experiments. Filtration and aliquoting were performed in a laminar flow cabinet under aseptic conditions. Aliquots were stored at −20 °C and thawed at 4 °C for 16 h prior to administration. The concentration and purity of TriF_HS_-MMAE after thawing were unchanged compared with pre-freezing values.

### Mass Spectrometry

Peptide LC-MS was carried out on an ACQUITY M-Class UPLC system coupled to a SYNAPT XS HRMS equipped with an ESI ion source. Mobile phase A consisted of H2O + 0.1% FA, while mobile phase B consisted of ACN + 0.1% FA. Approximately 10 pmol of peptide were injected, desalted on-system, and a 5-min 5–95% B linear gradient was applied for sample separation on a nanoEase M/Z BEH C18, 1.7 μm, 1 mm × 100 mm column, which was kept at 50 °C. MS data were collected at 1 scan/s over a 50–2000 m/z range in positive polarity and TOF resolution mode. Glufibrinopeptide B solution was acquired in the reference function, and the correction was applied in acquisition. Raw data were processed using MassLynx v4.2 software.

Intact protein LC-MS was carried out on an ACQUITY UPLC M-Class system coupled to a SYNAPT XS HRMS equipped with an ESI ion source interface. Mobile phase A consisted of H2O with 0.1% FA and 0.05% TFA, while mobile phase B consisted of ACN with 0.1% FA and 0.05% TFA. Approximately 2–5 pmol of protein were injected, desalted on-system, and a 5-min 20–80% B linear gradient was applied for sample separation on a nanoEase M/Z BEH C4, 300 Å, 5 μm, 300 μm × 50 mm column, which was kept at 80 °C. MS data were collected at 1 scan/s over an m /z 300–3000 range in positive polarity and TOF resolution mode. Glufibrinopeptide B solution was acquired using the reference function, and correction was applied during acquisition. Raw data were processed using MassLynx v4.2 software.

### Cytotoxicity Assay

Cells in the appropriate full medium were plated at 5000 cells per well in 96-well plates and incubated overnight at 37 °C in the presence of 5% CO_2_. Cells were treated with increasing concentrations (0.1 to 200 nM) of TriF_HS_ (negative control), TriF_HS_-MMAE or free drug (MMAE; positive control) for 96 h at 37 °C. Next, cell viability was measured using PrestoBlue Cell Viability Reagent (#A13262, Thermo Fisher Scientific, Waltham, MA, USA), according to the manufacturer’s protocol. Fluorescence emission at 590 nm (excitation at 560 nm), reflecting the viability of the cells, was measured using an Infinite M1000 PRO plate reader (Tecan, Männedorf, Switzerland). Statistical analyses were performed for three independent experiments, and IC_50_ values were calculated based on the Hill equation using OriginPro 2026 software (Northampton, MA).

### High-Throughput Screening

A high-content and high-throughput screen was performed to select chemical compounds that affect the efficiency of TriF_HS_ oligomer internalization. A working stock of the inhibitor library (Kinase Inhibitor Library HY-L009, MedChemExpress, Monmouth Junction, NJ, USA) was prepared (2653 chemical compounds at a final concentration of 50 μM). The HCS was performed using the hTERT-HPNE cell line. Cells (7000 cells per well) were preincubated with the inhibitors at a concentration of 4 μM in serum-free medium at 37 °C for 30 min. After this time, cells were incubated with 585 nM TriF_HS_ in serum-free medium at 37 °C for 30 min. As a reference control, cells were treated with only TriF_HS_. Without any preincubation. After this time, internalization was stopped by cooling the cells on ice. Cells were subsequently washed with PBS and fixed in 4% paraformaldehyde solution. Nuclei were stained with NucBlue Live dye, and cells were permeabilized with 0.1% Triton in PBS and stained with HCS CellMask Deep Red Stain. Fixed and labeled cells were analyzed with quantitative confocal microscopy using the Opera Phenix Plus High-Content Screening System and a robotic arm (PerkinElmer, Waltham, MA, USA). Measurements were carried out in confocal mode with a 63 × water, NA 1.15 objective, with 2× binning, using two-peak autofocus. 37 fields per well were imaged, with 8–10 z stacks per field at 0.5 μm intervals to ensure comprehensive imaging of the cells. 2160 × 2160 px camera ROI was used to capture the images. The Harmony High-Content Imaging and Analysis Software (versions 5.1 and 5.2; PerkinElmer, Waltham, MA, USA) was used for image acquisition and analysis. The number of cells and cell boundaries were determined using DAPI and CellMask Deep Red, respectively. The intensity of the fluorescent signal in the Alexa 488 channel was measured and compared between cells. Images were assembled in Adobe Illustrator, with only linear adjustments to contrast and brightness.

A high-content screen with selected inhibitors was performed using pancreatic adenocarcinoma cell lines: HPAF-II, Capan-2, PANC-10.05, and CFPAC 1 . Further steps were as described above.

### Cytotoxicity Assay with Selected Inhibitors

Verification of the effect of the inhibitors selected in the HCS screen on the performance of the TriF_HS_-MMAE was analyzed using confocal microscopy. hTERT-HPNE and HPAF-II cells (5000 cells per well) were pretreated with the inhibitors at a concentration of 4 μM in medium at 37 °C for 30 min. After this time, cells were incubated with TriF_HS_-MMAE conjugate. Control cells were incubated with TriF_HS_-MMAE conjugate, TriF_HS_ unconjugated protein, and the free drug MMAE (all at the concentration of EC_50_ calculated for each cell line) at 37 °C for 60 h and imaged in real time using confocal microscopy. As a reference, control cells were untreated and received no preincubation. Propidium iodide solution (1 μL of a 1 mg/mL stock solution) was added to visualize dead cells, and NucBlue Live (1 μL) was added to live cells. Cells were analyzed with quantitative confocal microscopy using the Opera Phenix Plus High-Content Screening System. Measurements were carried out in confocal mode with a 10× AIR, NA 1.15 objective, binning 2, using two-peak autofocus. The 2160 × 2160 px camera ROI was used to capture the images. The Harmony High-Content Imaging and Analysis Software (versions 5.1 and 5.2; PerkinElmer, Waltham, MA, USA) was used for image acquisition and analysis. The number of cells and the cells undergoing apoptosis were determined using DAPI and propidium iodide, respectively.

### *In Vivo* Tests

All animal experiments were performed in accordance with the European Directive 2010/63/EU and Polish regulations governing the use of animals in research. The study was approved by the Local Ethics Committee for Animal Experimentation in Wrocław, Poland (approval no. 061/2025). Female BALB/c mice (6–8 weeks old) were obtained from AnLab, s.r.o., Prague, Czech Republic, and housed under specific pathogen-free conditions with controlled temperature, humidity, and a 12-h light/dark cycle, with ad libitum access to food and water unless stated otherwise.

The *in vivo* study consisted of two consecutive stages: a pilot biodistribution study designed to define optimal tissue collection time points, and a main experiment assessing tissue exposure and toxicity-related endpoints of the TriF_HS_-MMAE conjugate administered alone or in combination with the kinase inhibitor crizotinib (hydrochloride). The pilot study informed the experimental design of the subsequent experiment and was performed using identical routes of administration and tissue processing procedures.

Ten BALB/c mice received a single intravenous injection of TriF_HS_-MMAE via the lateral tail vein at a dose corresponding to 0.38 mg/kg MMAE (23.86 mg/kg total conjugate), administered in PBS in a volume of 10 μL/g body weight. Animals were sacrificed at 30 min, 1, 6, 12, or 24 h postinjection (*n* = 2 mice per time point).

Untreated healthy mice were included as a negative control to determine tissue and plasma autofluorescence. Fluorescence-based biodistribution analysis revealed high systemic exposure at early time points, followed by a rapid decline in plasma signal, whereas selected organs exhibited distinct tissue-specific retention profiles. The most pronounced divergence between plasma clearance and tissue-associated fluorescence occurred between 1 and 6 h postinjection. On the basis of these observations, 1 h was selected to represent early distribution, and 3 h was chosen as an intermediate time point to capture ongoing tissue retention and early clearance kinetics in the main *in vivo* experiment.

For the main study, mice were randomized into experimental groups receiving: (i) PBS control, (ii) TriF_HS_-MMAE alone, (iii) crizotinib (hydrochloride) alone, or (iv) crizotinib (hydrochloride) followed by TriF_HS_-MMAE. Crizotinib (hydrochloride) was administered orally at 100 mg/kg, and TriF_HS_-MMAE was administered intravenously at the same dose and volume as in the pilot study. In combination groups, crizotinib (hydrochloride) was administered 4 h prior to TriF_HS_-MMAE injection, based on published pharmacokinetic data and pilot study timing. Mice were fasted for 12 h overnight prior to oral dosing of crizotinib or placebo, with free access to water. Fasting was applied uniformly across all experimental groups to minimize variability in drug absorption and systemic exposure. Animals were sacrificed at the two time points selected from the pilot study (1 and 3 h post-TriF_HS_-MMAE administration). At the designated end points, animals were anesthetized, and blood was collected from the retro-orbital sinus into EDTA-coated tubes immediately prior to euthanasia. Plasma was isolated by centrifugation at 2000 × *g* for 15 min at 4 °C, followed by a clarification step at 10,000 × *g* for 5 min at 4 °C. Plasma samples were protected from light and stored at −80 °C until analysis. Plasma biochemical analyses were performed on a Cobas C 111 analyzer (Roche Diagnostics, Switzerland), and blood morphological parameters were analyzed on a Mythic 18 hematology analyzer (PZ Cormay S.A., Lomianki, Poland) using reagents and procedures provided by the manufacturer.

Immediately after euthanasia, the liver, pancreas, kidneys, spleen, lungs, and brain were harvested, briefly blotted to remove surface blood, weighed, and subdivided for downstream analyses. Tissue fragments designated for fluorescence measurements were cut into 2–3-mm pieces and extensively washed in ice-cold PBS to remove residual intravascular blood. Washed samples were snap-frozen on dry ice and stored at −80 °C. Frozen tissues were homogenized using a FastPrep-24 homogenizer (MP Biomedicals, Santa Ana, CA, USA) with ceramic beads under cold conditions. All samples were processed under light-protected conditions to preserve GFP fluorescence.

Fluorescence derived from the GFP-labeled TriF_HS_-MMAE conjugate was quantified using a BioTek Synergy H4 plate reader with a fixed detector sensitivity (gain of 100). Excitation was set to 485 nm (bandwidth of 9 nm). Emission was collected at 512 nm using a bandwidth of 17 nm for plasma, spleen, brain, lungs, and pancreas, and 9 nm for kidney and liver. To ensure comparability, fluorescence readings were acquired in a cross-measurement scheme across all tissue types. Raw fluorescence values (RFU) were normalized to total protein content measured in the corresponding plasma or tissue homogenates using the DC Protein Assay (Bio-Rad, Cat. No. 5000112) and are reported as RFU per mg of total protein (RFU/mg TP). Background autofluorescence was determined from plasma and tissues of an untreated control mouse and used as the blank reference.

The pilot biodistribution study was analyzed descriptively to identify temporal trends and inform the experimental design. The main experiment was designed to test predefined hypotheses regarding tissue exposure and toxicity modulation. Details of the statistical analysis are provided in the corresponding figure legends.

### Total RNA Isolation and Quantitative Real-Time PCR

Total RNA was extracted from 50 mg of mouse liver and pancreatic tissues using QIAzol Lysis Reagent (Qiagen, catalog no. 79306) in combination with glass bead-assisted mechanical disruption.^65^ The homogenized samples were then subjected to organic phase separation in accordance with the manufacturer’s instructions, which enabled the selective enrichment of RNA in the aqueous phase. Purification was completed using standard alcohol-based extraction and washing procedures that are compatible with QIAzol-processed lysates. The integrity and concentration of the RNA were assessed using a Multiskan SkyHigh Microplate Spectrophotometer (Life Technologies, Warsaw, Poland).

The TaqMan RNA-to-CT 1-Step Kit (Applied Biosystems, catalog no. 4392938) was used as previously described, following the manufacturer’s protocol, including a 20 min reverse transcription step at 50 °C. Expression levels of the proteoglycan-encoding gene set (Supplemental Table 5) were quantified using TaqMan probes in three technical replicates derived from two mice. Relative gene expression was calculated using the 2^(−ΔΔCt)^ method.^66^ For pancreatic tissue, *GAPDH* and succinate dehydrogenase complex flavoprotein subunit A (*SDHA*) served as reference genes; for liver tissue, *GAPDH*, *SDHA*, and β-actin (*Actb*) were used as reference genes. All probes were obtained from Life Technologies (Warsaw, Poland). The results were averaged and normalized to the mean gene expression level in samples collected 30 min after conjugate administration. The results are presented as the mean ± standard deviation (SD). Statistical significance was determined using two-way ANOVA and Fisher’s LSD test, with a P value less than 0.05 considered significant.

### Preparation of Tissue Samples for Western Blotting

Separated murine tissues were collected immediately after sacrifice and frozen for Western blotting. Frozen tissues were pulverized under liquid nitrogen using a prechilled mortar and pestle, weighed, and resuspended in lysis buffer (50 mM Tris, 0.4% SDS, pH 6.8) supplemented with cOmplete Protease Inhibitor Cocktail (1 tablet per 40 mL buffer). Samples were sonicated on ice (amplitude 60%; three cycles of 10 s ON/20 s OFF) and heated at 98 °C for 10 min to ensure complete protein denaturation in the presence of SDS. Lysates were centrifuged at 12,000 × *g* for 15 min at 4 °C, and the clarified supernatant was transferred to a fresh tube to remove insoluble debris. Protein concentrations were determined using the Pierce BCA Protein Assay Kit (#23227) from Thermo Fisher Scientific (Waltham, MA, USA), according to the manufacturer’s instructions. For each tissue sample, an equal amount of protein (20 μg per lane) was used for SDS-PAGE electrophoresis.

### Histopathological Analyses

Mouse tissues were collected immediately after sacrifice and fixed in 10% neutral-buffered formalin (NBF; pH 7.2–7.4) for 24 h at room temperature, maintaining a fixative-to-tissue volume ratio of at least 10:1. Following fixation, samples were processed using a routine automated tissue-processing protocol on a tissue processor (Excelsior AS, Thermo Fisher Scientific, USA). The processing procedure included graded dehydration through increasing concentrations of ethanol (70%, 80%, 96%, and 100%), clearing in xylene, and paraffin infiltration at 58–60 °C. Subsequently, tissues were embedded in paraffin blocks using a semi-automatic embedding station (HistoStar, Thermo Fisher Scientific, USA), ensuring careful orientation of the specimens. The paraffin blocks were allowed to solidify at room temperature and were stored until further histological and immunohistochemical analyses.

Formalin-fixed, paraffin-embedded (FFPE) tissue blocks were sectioned at a thickness of 4 μm using a rotary microtome (CUT 5062, SLEE Medical GmbH, Germany). The sections were floated on a 40 °C water bath to minimize wrinkles and were mounted on glass slides. Slides were dried for 30 min at 45 °C to ensure optimal adhesion. For deparaffinization, slides were incubated in fresh xylene (two changes, 5 min each), followed by rehydration through a graded ethanol series (100%, 96%, 80%, and 70%), then rinsed in distilled water. Sections were stained with Harris hematoxylin (Harris Hematoxylin, S Series, Epredia, USA) for 10 min, rinsed in running tap water for 5 min, and differentiated in 1% acid alcohol (HCl in 70% ethanol, Nu-Clear, Epredia, USA) for several seconds until optimal nuclear contrast was achieved. After differentiation, sections were counterstained with alcoholic eosin (Eosin-Y, R Series, Epredia, USA) for 40 s, rinsed briefly in distilled water, and dehydrated through increasing concentrations of ethanol (70%, 80%, 96%, and 100%), followed by clearing in xylene (two changes, 5 min each). To ensure reproducibility and consistent staining quality across samples, all H&E staining procedures were performed using an automated protocol under standardized conditions. Finally, slides were mounted with DPX mounting medium and coverslipped. Histopathological evaluation of the overall tissue architecture and morphological alterations was performed using a light microscope (Olympus BX46, Evident, Japan).

### Statistics

For comparisons involving more than two groups, statistical significance was evaluated using one-way ANOVA when assumptions of normality and homogeneity of variance were satisfied. In cases where the number of observations exceeded 100 per group, one-way ANOVA was applied based on the Central Limit Theorem. Post hoc comparisons were performed using Tukey’s HSD test, the Spjøtvoll–Stoline modified Tukey HSD test for unequal N per group, or the Games–Howell test when equality of variances was not assumed. When parametric assumptions were not met, group differences were assessed using the Kruskal–Wallis test with multiple comparisons. For comparisons involving two independent variables, statistical analyses were performed using two-way ANOVA followed by Fisher’s LSD post hoc test. For comparisons between two groups, statistical significance was assessed using Student’s *t*-test. Significance levels were defined as *p < 0.05, **p < 0.01, ***p < 0.001, ****p < 0.0001. The color gradient in the microarrays represents group means calculated from the raw data and normalized to the control mean. In statistical analyses performed for inhibitor selection, HCS data sets were subjected to outlier removal using the Normal two-sided test.

## Supplementary Material





## Data Availability

Source data are provided with this paper. Original data used for the preparation of this manuscript have been deposited in the Zenodo repository and are available under the following doi: 10.5281/zenodo.15591869.

## Notes

Internalization kinetics of TriF_HS_ (Alexa 488) to hTERT-HPNE cells. Nuclei were stained using NucBlue Live. Images were captured every 1 s (WMV)
Supplementary Figures 1–48 with details of: developed protein characterization; the mechanism of endocytosis and intracellular trafficking of TriF_HS_; mass spectra of TriF_HS_-MMAE synthesis intermediates; BLI binding of developed proteins to GPC4; TriF_HS_-MMAE toxicity in the presence of heparin; HSPG expression levels in tested cells; HCS experiments optimization and example microscopy results; assignment of kinases modulating TriF_HS_ endocytosis; the impact of selected kinase inhibitors on cell viability, TriF_HS_-MMAE aggregation, HSPG clustering and endocytosis; the impact of crizotinib and TriF_HS_-MMAE on microtubule architecture and cell viability; pilot *in vivo* tests; and selected tests from the main *in vivo* experiments in a mouse model; Supplementary Tables 1–5 with HCS-identified modulators of TriF_HS_-MMAE endocytosis and probes used for measurements of HSPG expression level; Amino acid sequences of developed proteins (PDF)
