# Supplementary material for: Priming Cells with Modulators of Cellular Signaling Ensures Enhanced Selectivity of HSPG-Specific Protein–Drug Conjugate Delivery
Source: J Am Chem Soc. 2026 Aug 28;148(35):37884–908. doi: 10.1021/jacs.6c08409 (PMC13564429; doi:10.1021/jacs.6c08409)
Supplement: Supplementary file 1 [file ja6c08409_si_002.pdf]

Supplementary data for:

Priming cells with modulators of cellular signaling ensures enhanced selectivity of HSPG-specific protein-drug conjugate delivery

Aleksandra Chorążewska[1#], Krzysztof Ciura[1#], Magdalena Milczarek[2], Dagmara Kłopotowska[2], Adam Pomorski[3], Anna Więch-Walów[4], Błażej Łukianowski[5,6], Paweł Gajdzis[5,6], Artur Krężel[3], Rafał Bartoszewski[4], Joanna Wietrzyk[2], Liliana Schaefer[7], Natalia Porębska[1*] and Łukasz Opaliński[1*]

[1]Department of Medical Biotechnology, Faculty of Biotechnology, University of Wroclaw, Joliot-Curie 14a, 50-383 Wroclaw, Poland

[2]Department of Experimental Oncology, Hirszfeld Institute of Immunology and Experimental Therapy, Wroclaw, Weigla 12, 53-114 Poland

[3]Department of Chemical Biology, Faculty of Biotechnology, University of Wroclaw, Joliot-Curie 14a, 50-383 Wroclaw, Poland

[4]Department of Biophysics, Faculty of Biotechnology, University of Wroclaw, Joliot-Curie 14a, 50-383 Wroclaw, Poland

[5]Department of Clinical and Experimental Pathology, Wroclaw Medical University, T. Marcinkowskiego 1, 50-368, Wroclaw, Poland

[6]Department of Pathomorphology, 4th Military Hospital, Weigla 5, 53-114 Wroclaw, Poland

[7]Institute of Pharmacology and Toxicology, Goethe University, Theodor-Stern Kai 7, 605090 Frankfurt, Germany

#These authors contributed equally

*Correspondence should be addressed to L.O (lukasz.opalinski@uwr.edu.pl) and N.P (natalia.porebska@uwr.edu.pl)

Supplementary Fig. 1

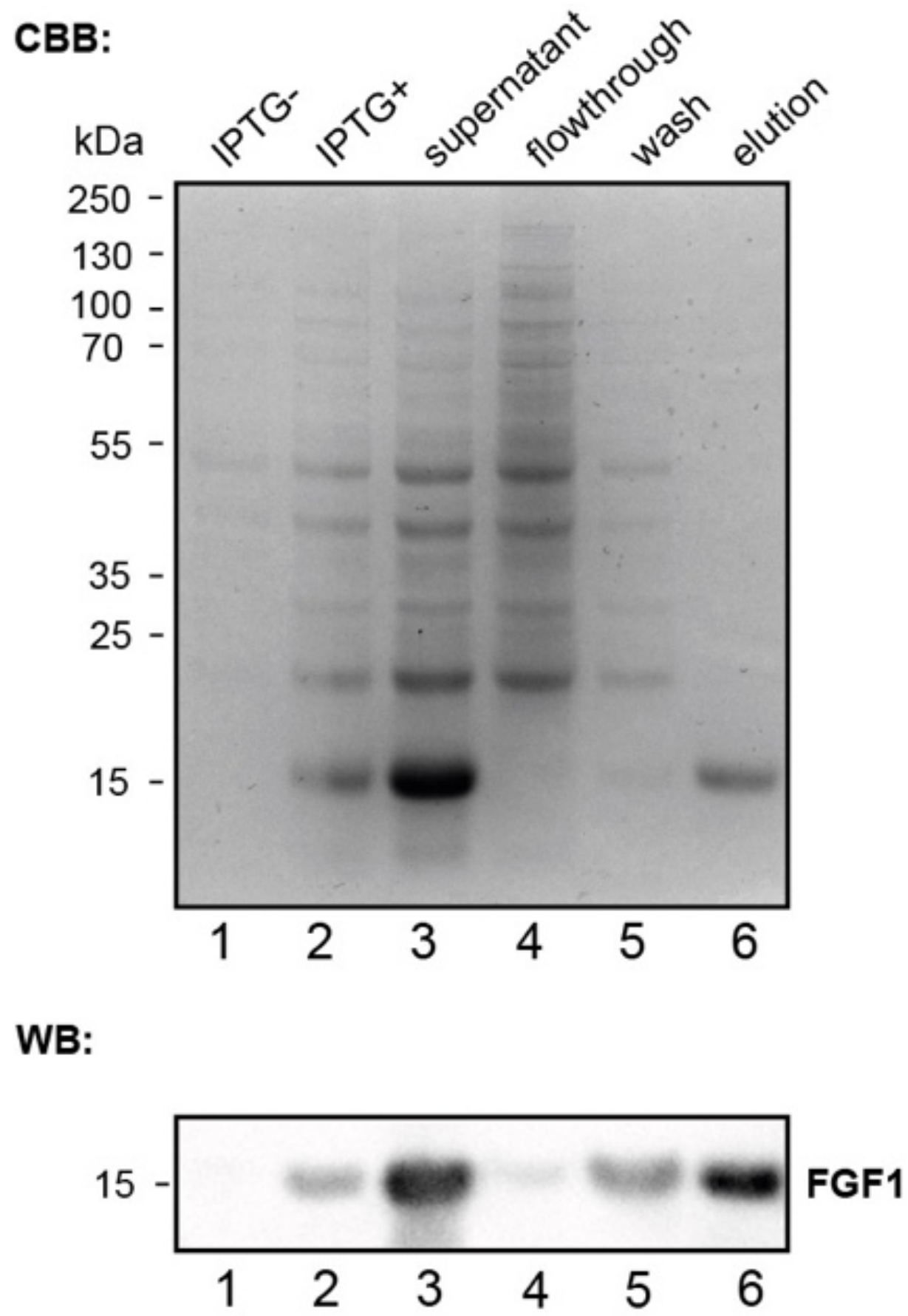

Supplementary Figure 1. Overproduction, purification and identification of FGF1$_{HS}$. FGF1$_{HS}$ was purified using Heparin Sepharose 6 Fast Flow resin. CBB refers to Coomassie Brilliant Blue stained SDS-PAGE gel. WB refers to Wester-Blotting analysis with primary anti-FGF1 antibodies. 1 – IPTG-, 2 – IPTG+, 3 – supernatant, 4 – flowthrough, 5 – wash 0.5 M NaCl, 6 – elution 2 M NaCl.

Supplementary Fig. 2

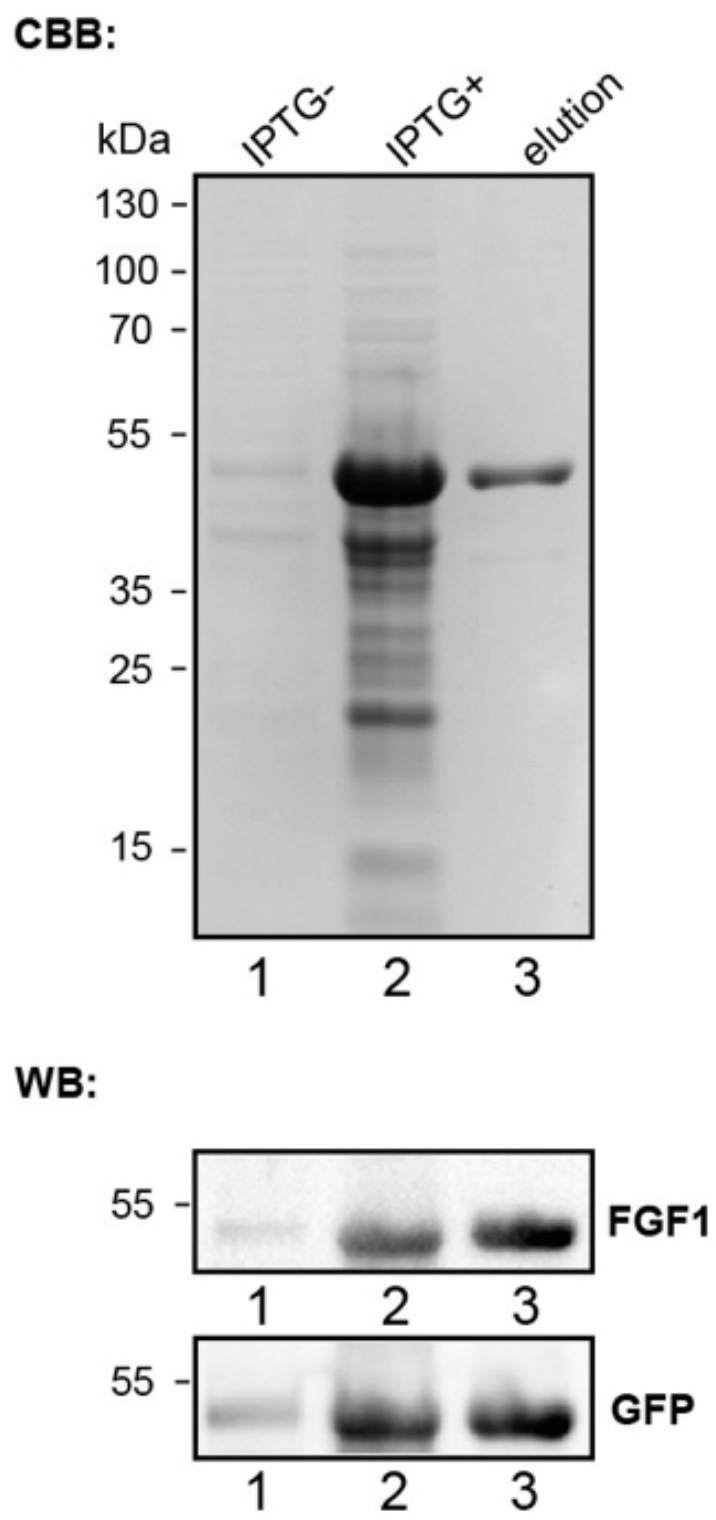

Supplementary Figure 2. Overproduction, purification and identification of GFPp-FGF1$_{HS}$. GFPp-FGF1$_{HS}$ was purified using Heparin Sepharose 6 Fast Flow resin. CBB refers to Coomassie Brilliant Blue stained SDS-PAGE gel. WB refers to Wester-Blotting analysis with primary anti-FGF1 or anti-GFP antibodies. 1 – IPTG-, 2 – IPTG+, 3 – elution 2 M NaCl.

Supplementary Fig. 3

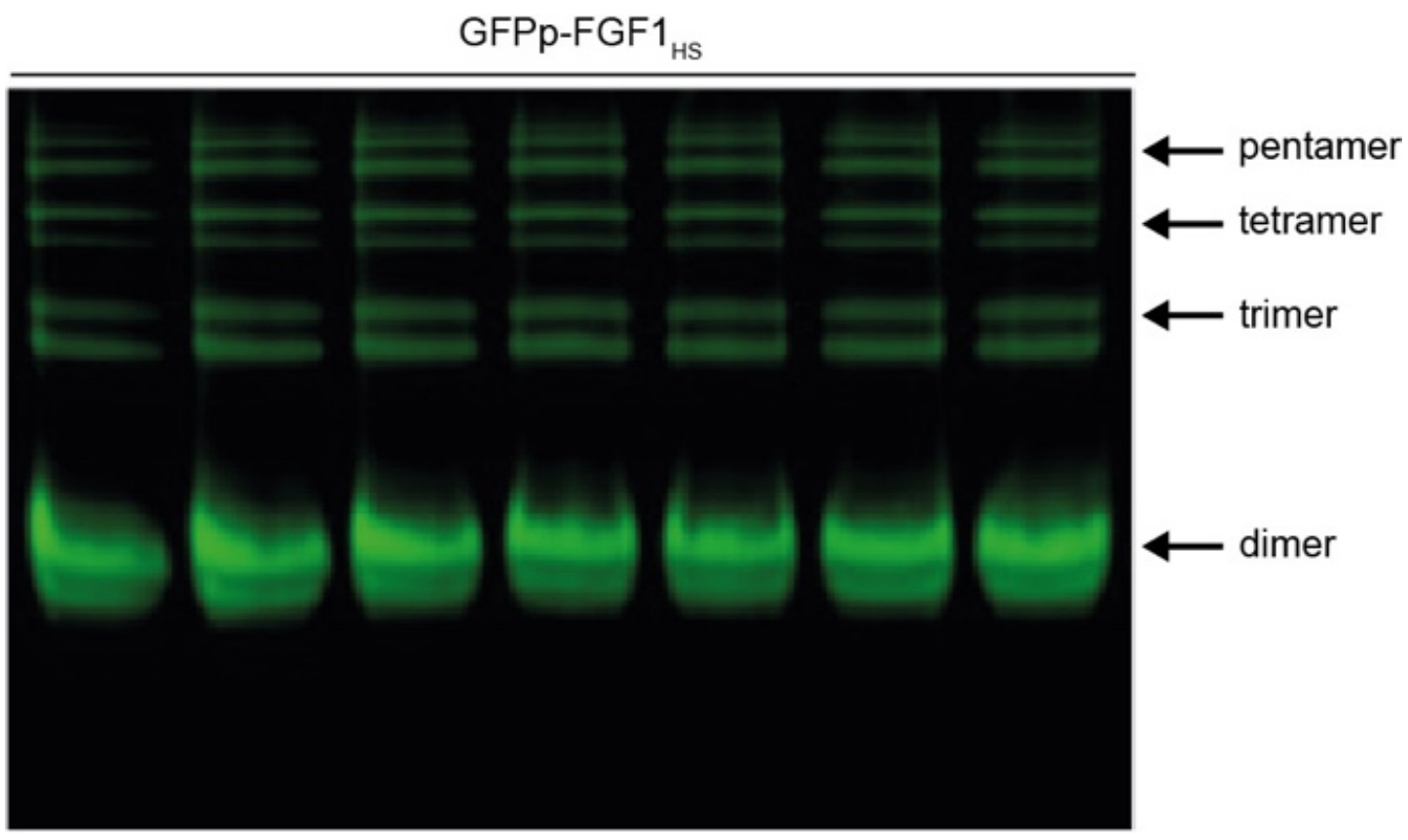

Supplementary Figure 3. Fluorescence signal obtained from Native-PAGE analysis of GFPp-FGF1$_{HS}$. Each oligomeric state is represented by 2 bands – linear and circular form.

Supplementary Fig. 4

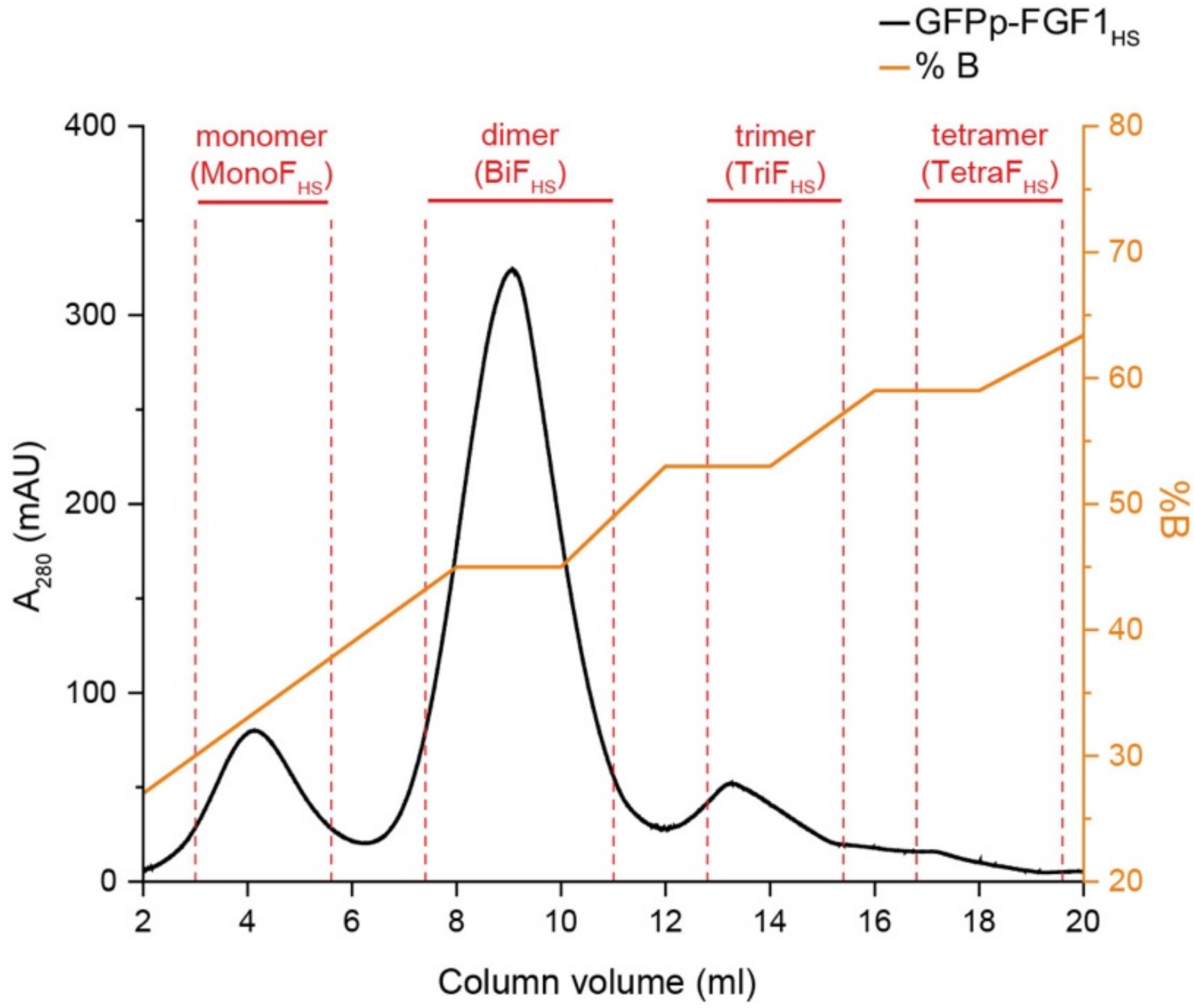

Supplementary Figure 4. FPLC-based separation of GFPp-FGF1$_{HS}$ oligomeric forms. Distinct oligomeric states of GFPp-FGF1$_{HS}$ (indicated in red) were resolved by fast protein liquid chromatography (FPLC) using a HiTrap Heparin HP column. Elution was performed with a combined gradient/isocratic NaCl profile (orange line), and monitored by UV absorbance at 280 nm (black line).

Supplementary Fig. 5

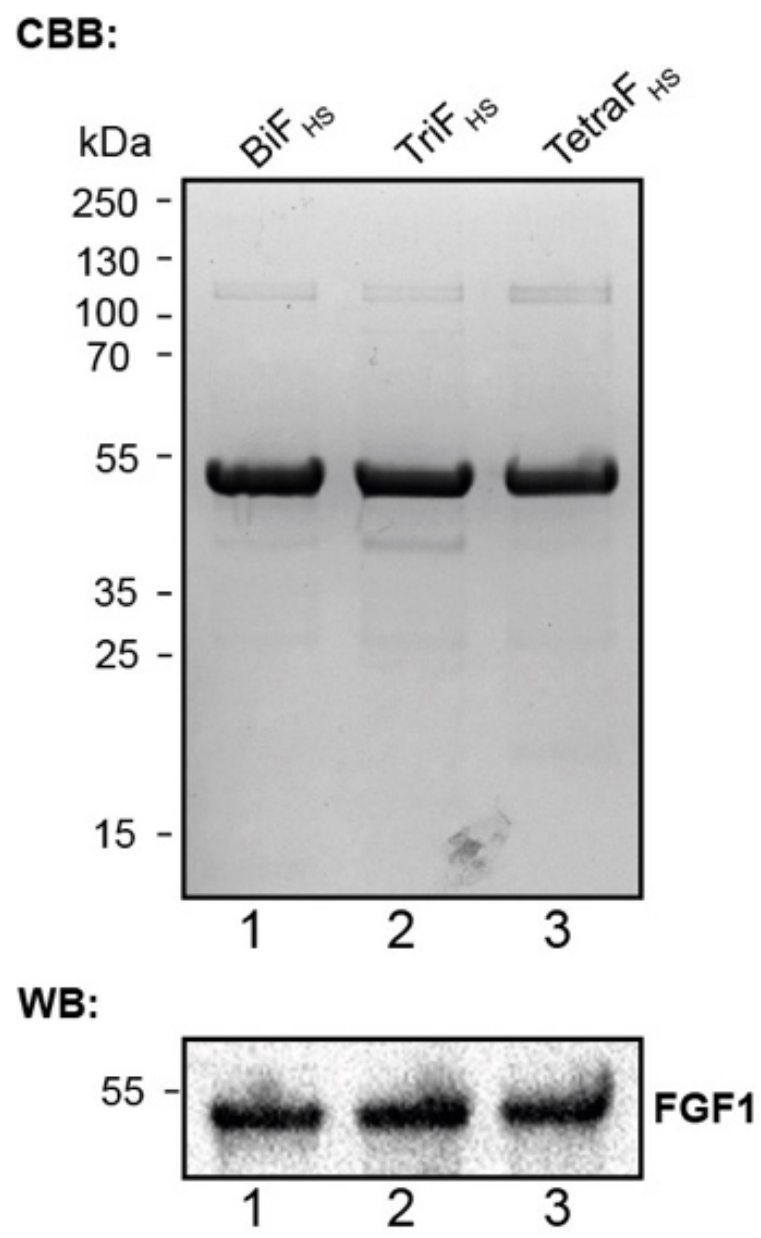

Supplementary Figure 5. Analysis of identity and purity of BiF$_{HS}$, TriF$_{HS}$ and TetraF$_{HS}$ under denaturing conditions. CBB refers to Coomassie Brilliant Blue stained SDS-PAGE gel. WB refers to Wester-Blotting analysis with primary anti-FGF1 antibodies. 1 – BiF$_{HS}$, 2 – TriF$_{HS}$, 3 – TetraF$_{HS}$. 2 µg of each oligomeric states were used for SDS-PAGE gel electrophoresis.

Supplementary Fig. 6

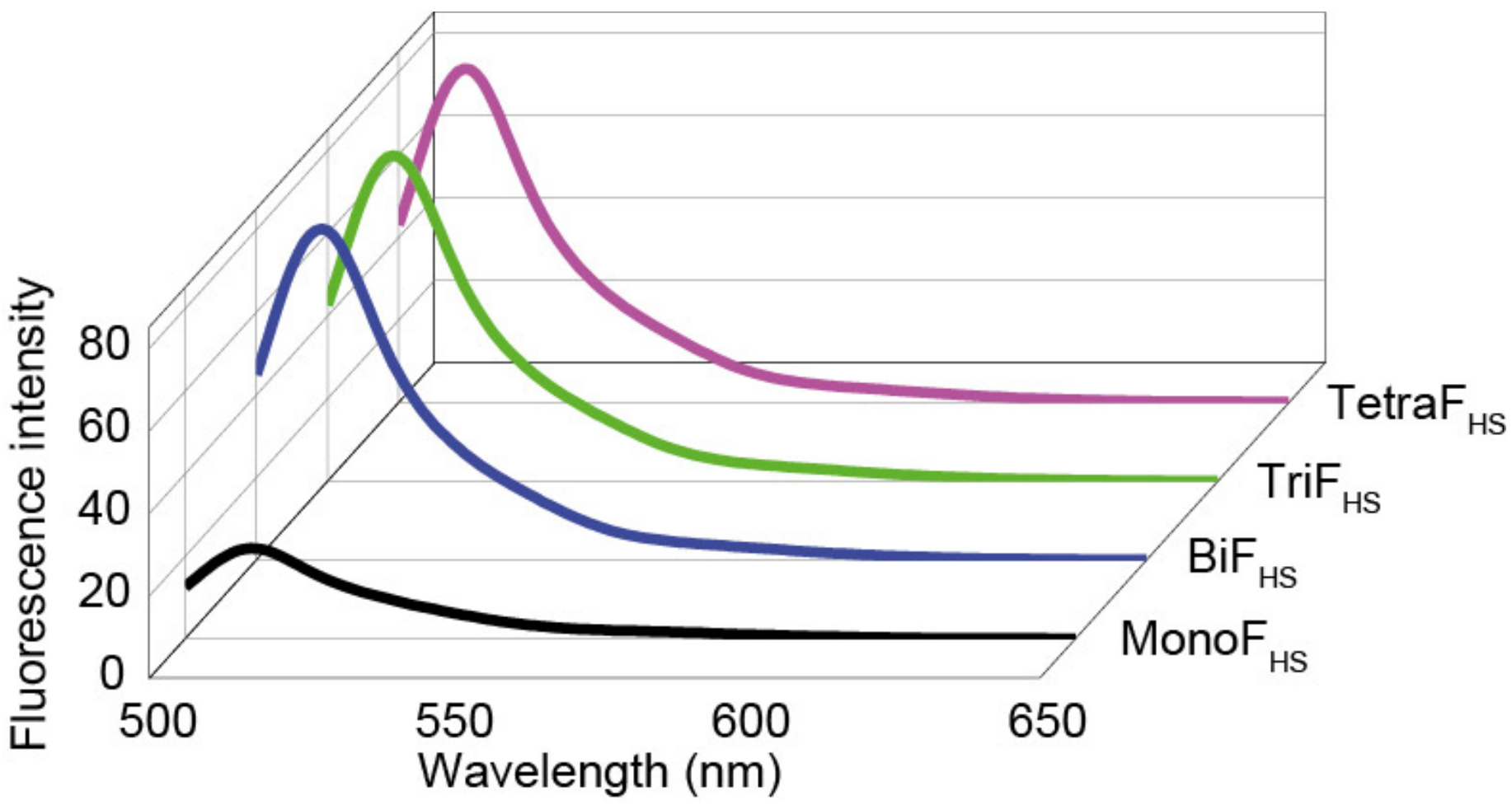

Supplementary Figure 6. Fluorescence emission spectra of MonoF$_{HS}$, BiF$_{HS}$, TriF$_{HS}$, and TetraF$_{HS}$. Fluorescence emission profiles were recorded in the range of 500–670 nm under identical experimental conditions for all samples. Each oligomeric form of GFPp-FGF1$_{HS}$ was measured at a final concentration of 200 nM.

Supplementary Fig. 7

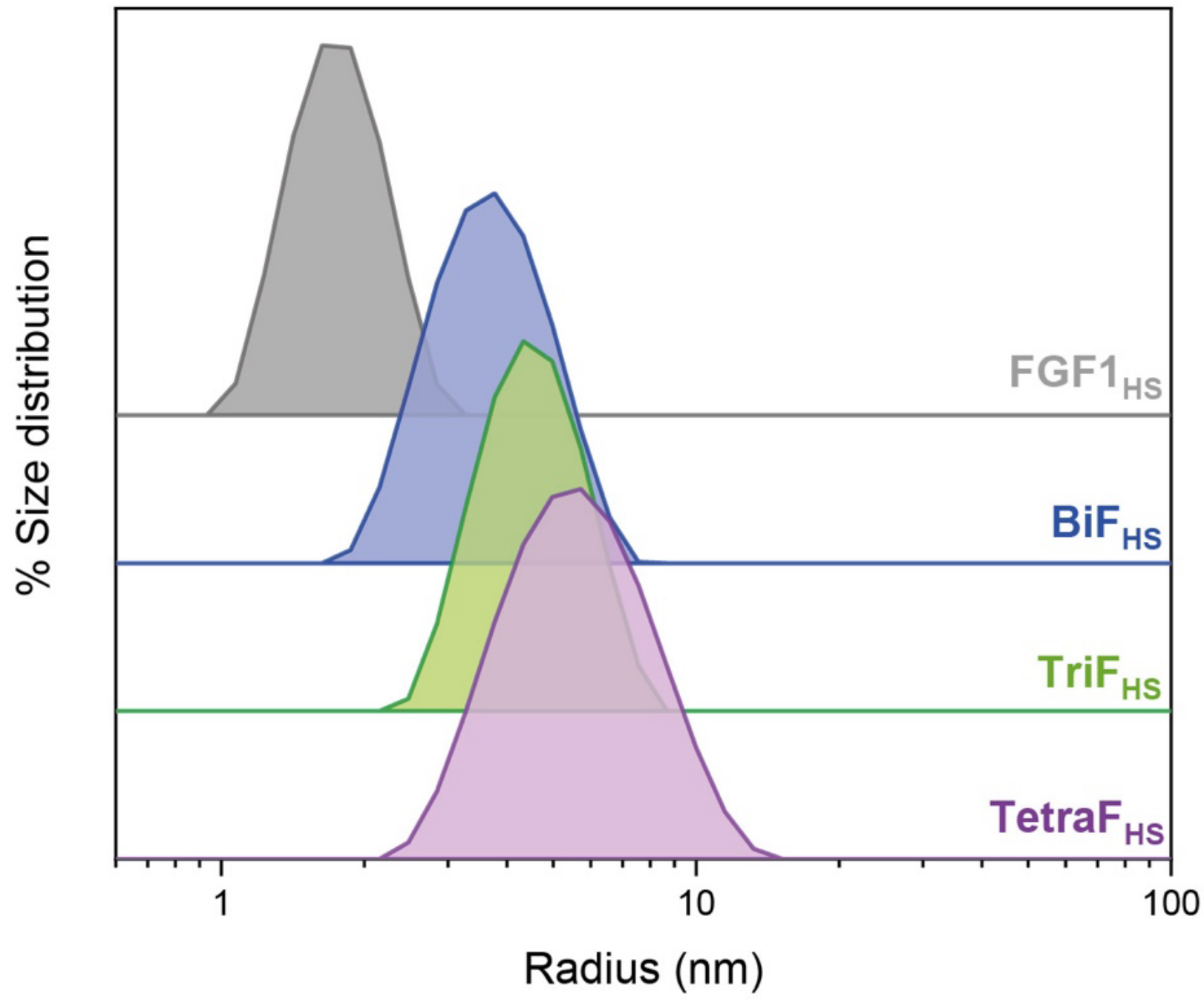

Supplementary Figure 7. DLS analyses of developed proteins. The oligomeric state of the purified proteins ($FGF1_{HS}$, BiF_{HS}, $TriF_{HS}$, $TetraF_{HS}$) was analyzed at 10 μM concentration of proteins in PBS bufefer using a Prometheus Panta instrument (NanoTemper Technologies GmbH, Munich, Germany). Samples were equilibrated to room temperature prior to acquisition.

Supplementary Figure 8

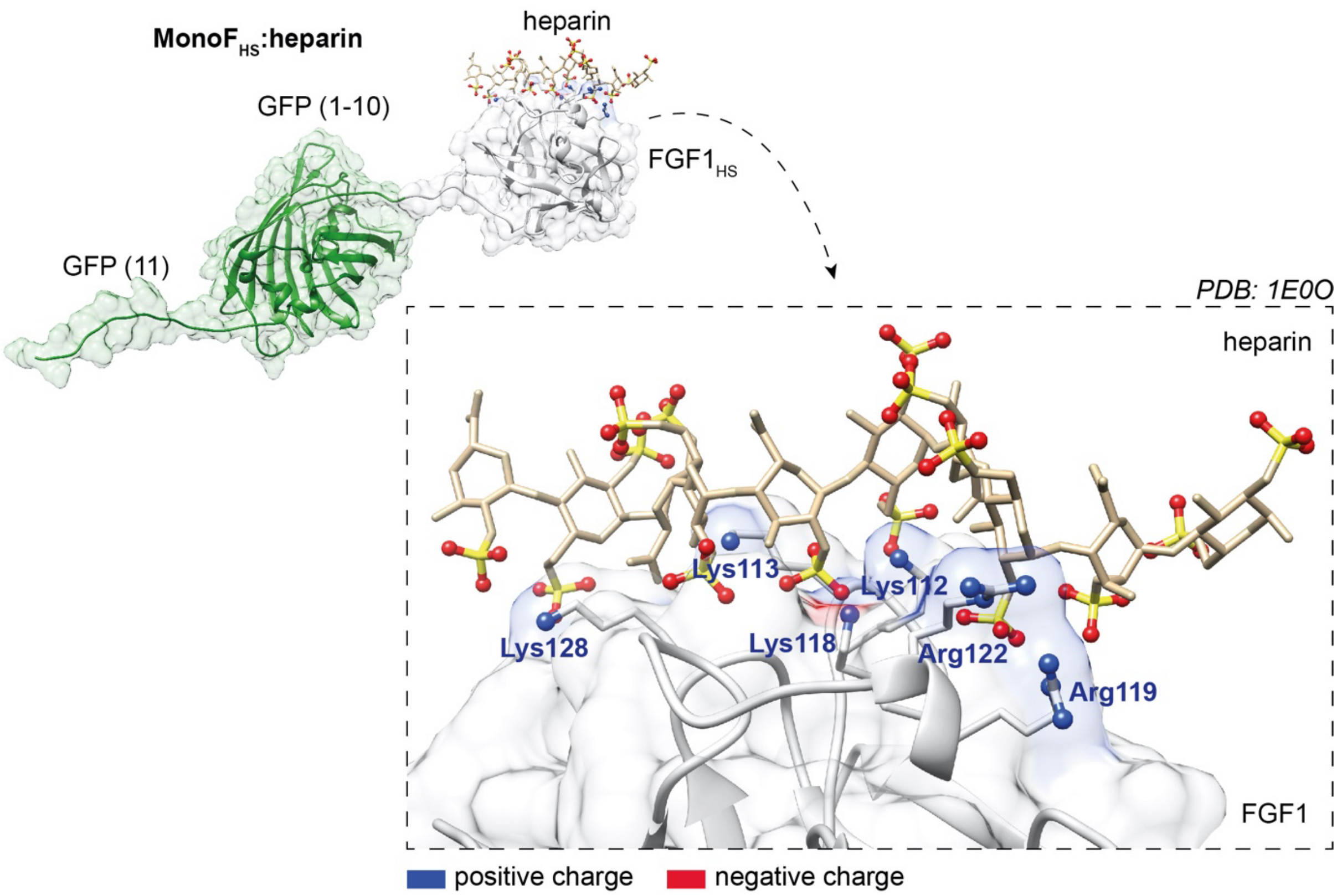

Supplementary Figure 8. Structural model of the interaction between the MonoF$_{HS}$ and heparin. Heparin is shown in a ball-and-stick representation, and the protein in ribbon/surface representation. Amino acid residues involved in electrostatic interactions with the ligand are indicated; positive and negative charges are shown in blue and red, respectively. Since FGF1 is responsible for HS binding this model should be valid all developed oligomers.

Supplementary Fig. 9

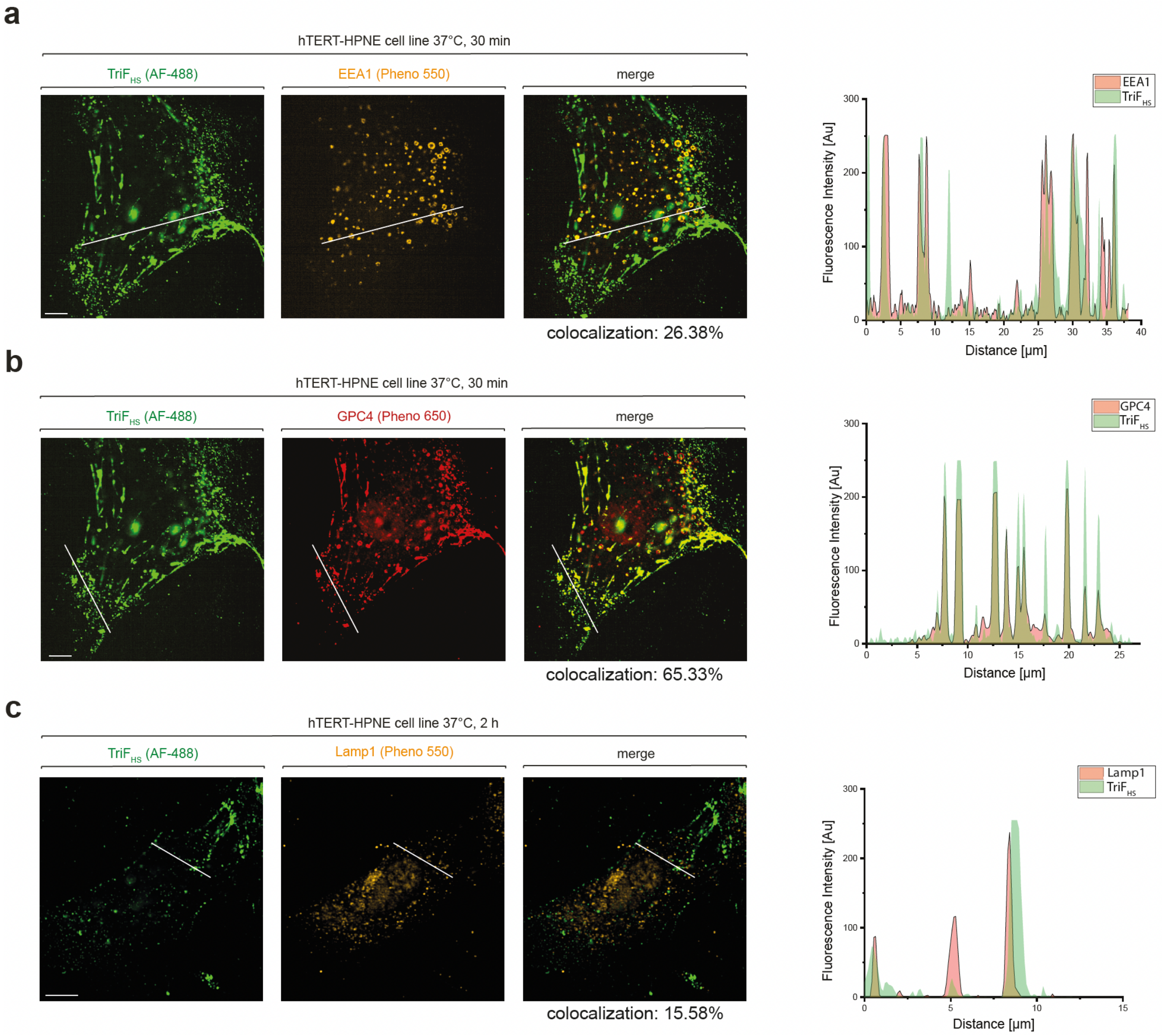

Supplementary Figure 9. Quantification of colocalization of $TriF_{HS}$ with endosomes, GPC4 and lysosomes. Microscopy images of hTERT-HPNE cells treated with $TriF_{HS}$. For each cell, the percentage of $TriF_{HS}$ colocalization with a given marker was calculated in the whole cell. The number of $TriF_{HS}$ spots from the Alexa488 channel was calculated for the entire cell and then for the region of interest (ROI) defined for a given organelle/protein. In addition, fluorescent intensity profiles were generated for each condition. The white line in each image indicates the position along which the intensity profiles were measured for both channels.

Supplementary Fig. 10

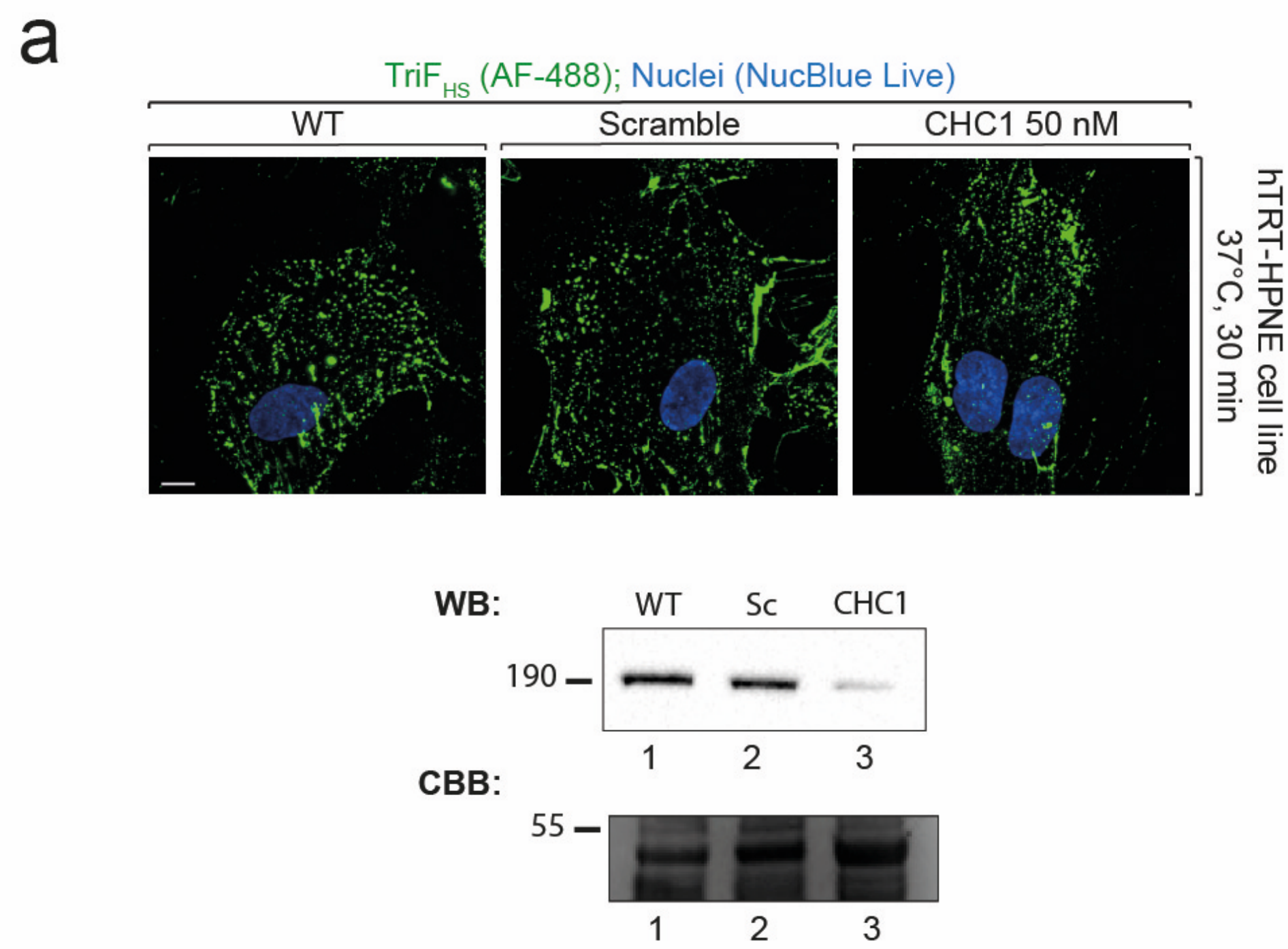

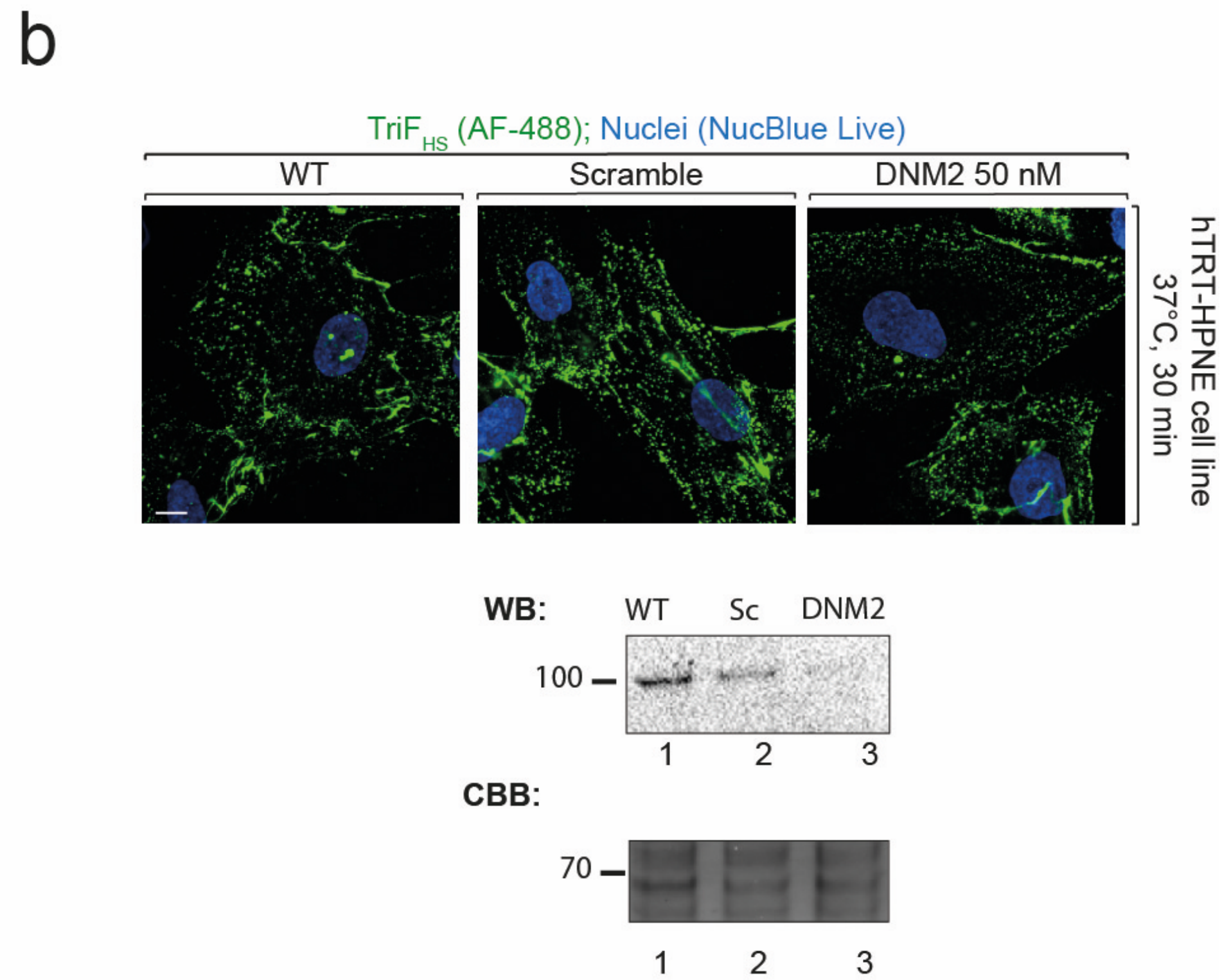

Supplementary Figure 10. Analysis of endocytosis mechanism used by $TriF_{HS}$. **A.** The effect of knock-down of clathrin heavy chain (CHC1) on $TriF_{HS}$ internalization. Knock down was confirmed with Western-blotting technique; 1 - wild type (WT) cells, 2 – scramble (Sc), cells treated with non-targeted siRNA; 3 - CHC1 – cells treated with siRNA against CHC1. Microscopy images of hTERT-HPNE cells treated with $TriF_{HS}$ (Alexa 488) for 30 min in 37°C. Nuclei were stained using NucBlue Live and cytoplasm was stained using CellMask Deep Red. **B.** The analysis of the role of DNM2 in $TriF_{HS}$ endocytosis. DNM2 was knocked-down with siRNA; effectiveness of silencing was confirmed using Western-blotting technique; 1 - wild type (WT) cells, 2 – scramble (Sc), cells treated with non-targeted siRNA; 3 – DNM2 – cells treated with siRNA against DNM2. Microscopy images of hTERT-HPNE cells treated with $TriF_{HS}$ (Alexa 488) for 30 min in 37°C. Nuclei were stained using NucBlue Live and cytoplasm was stained using CellMask Deep Red. Scale bar represents 10 μm.

Supplementary Fig. 11.

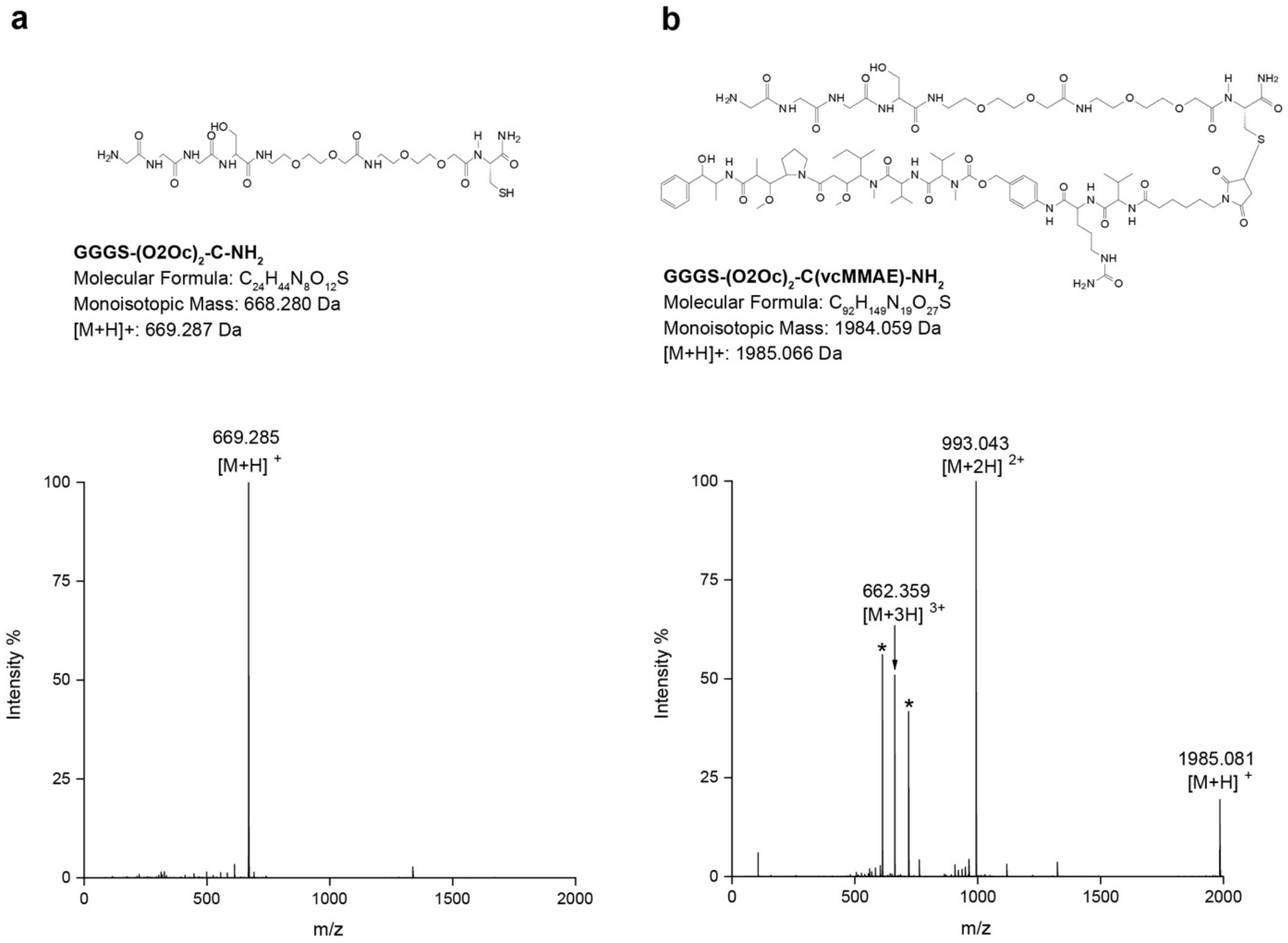

Supplementary Figure 11. Mass spectra of H$_2$N-GGGS-(O2Oc)$_2$-C-amide peptide and GGGS-(O2Oc)2-C-vcMMAE using ESI-MS. On the top, molecular formula, monoisotopic mass and ion mass was presented for both H$_2$N-GGGS-(O2Oc)$_2$-C-amide (**A**) and GGGS-(O2Oc)2-C-vcMMAE (**B**). Raw data was processed using the MassLynx V4.2 software. Peaks marked with an asterisk (*) corresponds to in-source fragments.

Supplementary Fig. 12

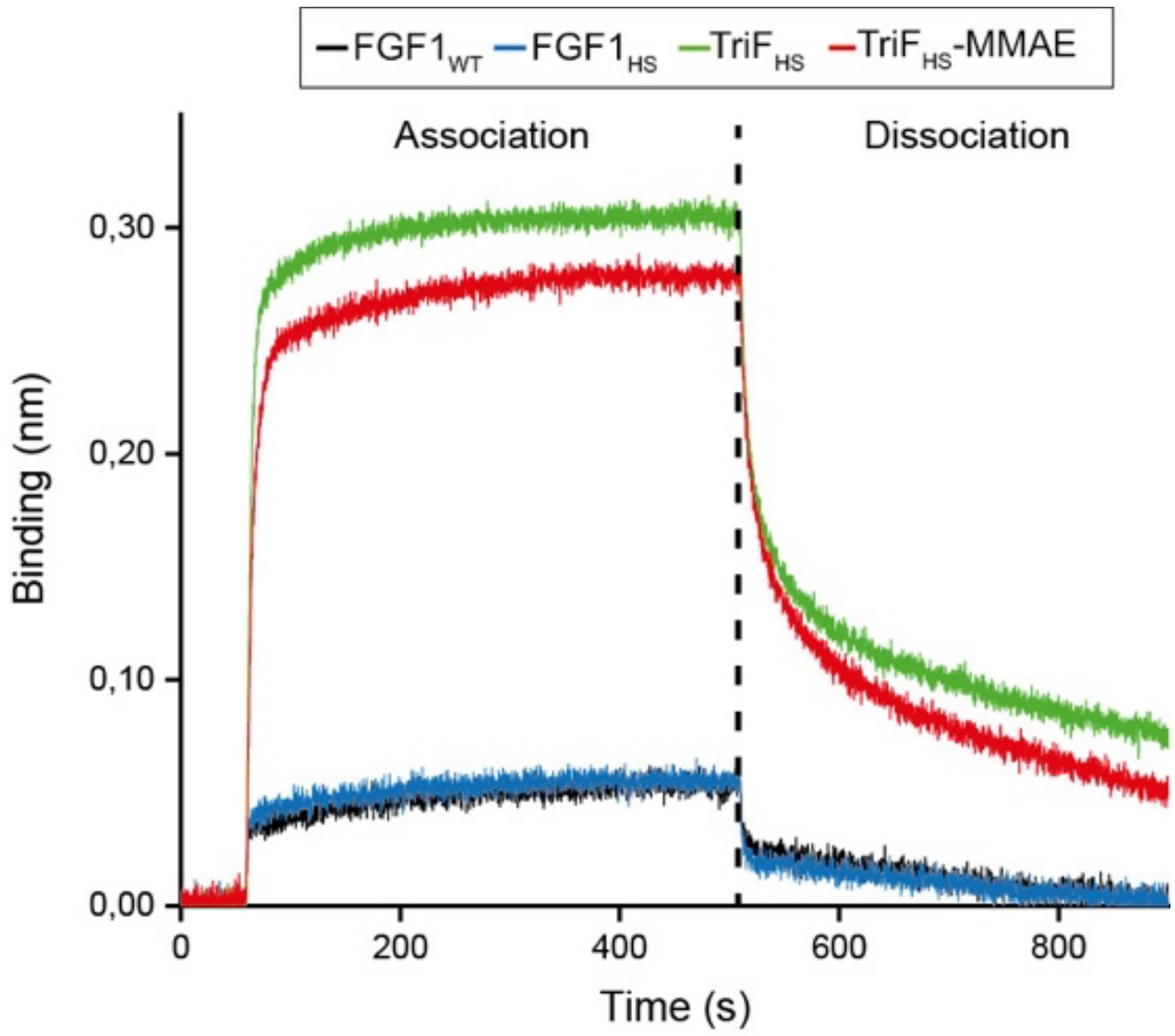

Supplementary Figure 12. Binding analysis of FGF1$_{WT}$, FGF1$_{HS}$, TriF$_{HS}$ and TriF$_{HS}$-MMAE to sensor-immobilized GPC4-Fc Chimera Protein. Sensors were subsequently incubated with 300 nM samples of FGF1$_{WT}$ (black), FGF1$_{HS}$ (blue), TriF$_{HS}$ (green) and TriF$_{HS}$-MMAE (red) for 450 s, followed by 450 s dissociation step. Each measured signal was control- (the Fc), and baseline-subtracted.

Supplementary Fig. 13

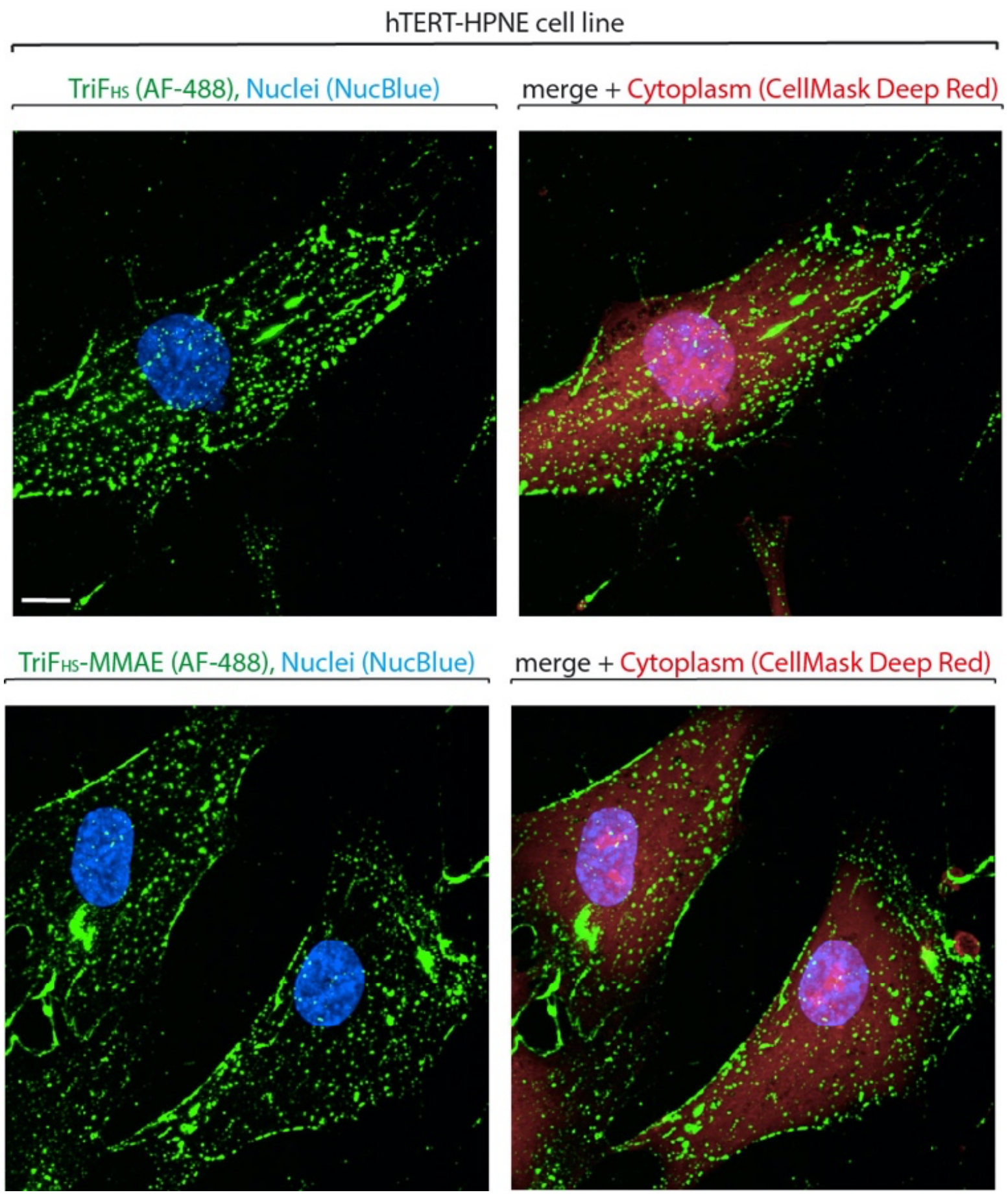

Supplementary Figure 13. Endocytosis of TriF$_{HS}$ and TriF$_{HS}$-MMAE to normal pancreatic cells. Microscopy images of hTERT-HPNE cells treated with TriF$_{HS}$ or TriF$_{HS}$-MMAE for 30 min in 37°C. Nuclei were stained using NucBlue Live and cytoplasm was stained using CellMask Deep Red. The endocytosis was monitored using Opera Phenix Plus confocal microscopy. Scale bar represents 10 µm.

Supplementary Fig. 14

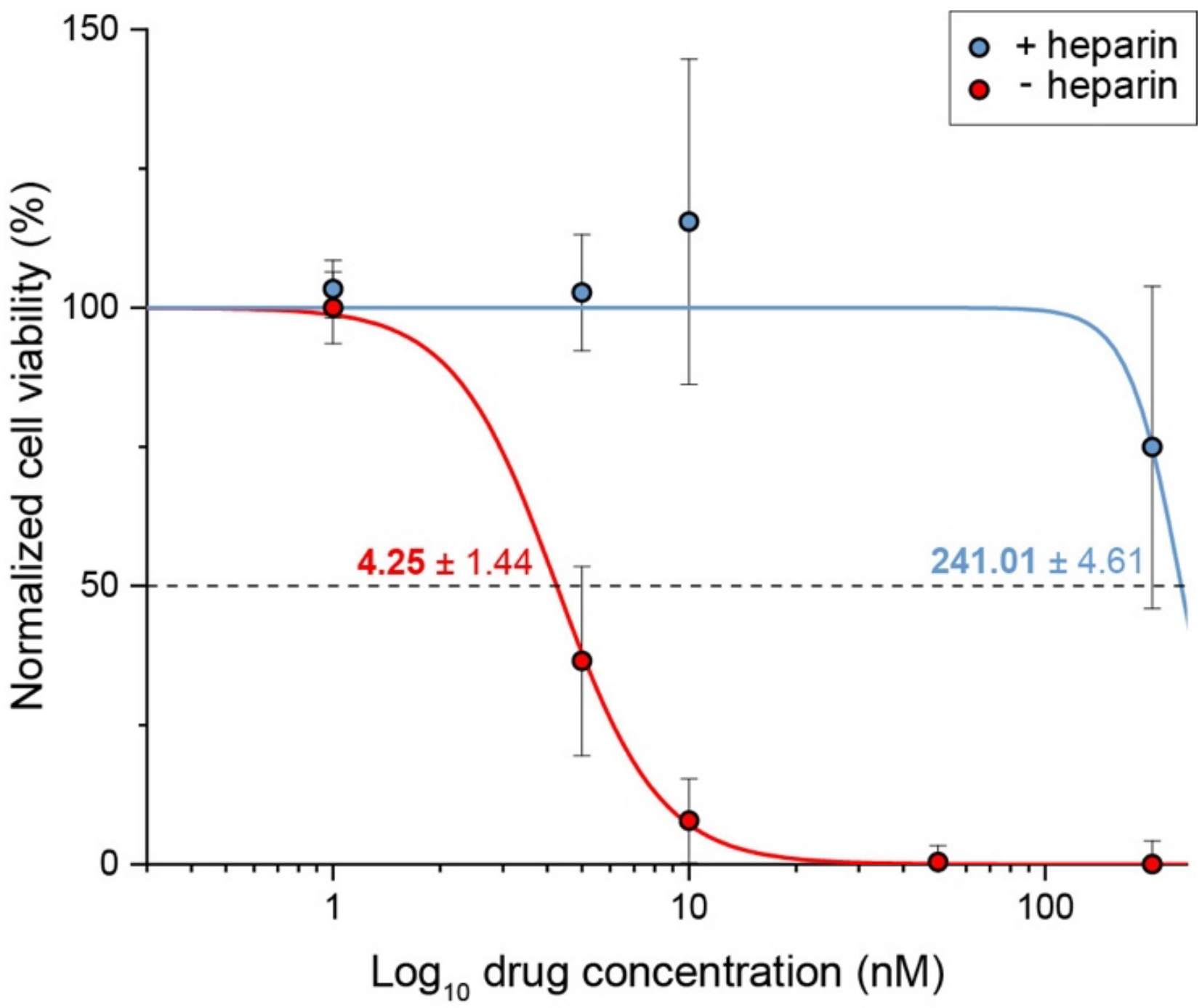

Supplementary Figure 14. Dose–response curves showing normalized cell viability of HPAF-II cells treated with increasing concentrations of TriF$_{HS}$-MMAE in the presence or absence of heparin. EC_{50} values were indicated.

Supplementary Fig. 15

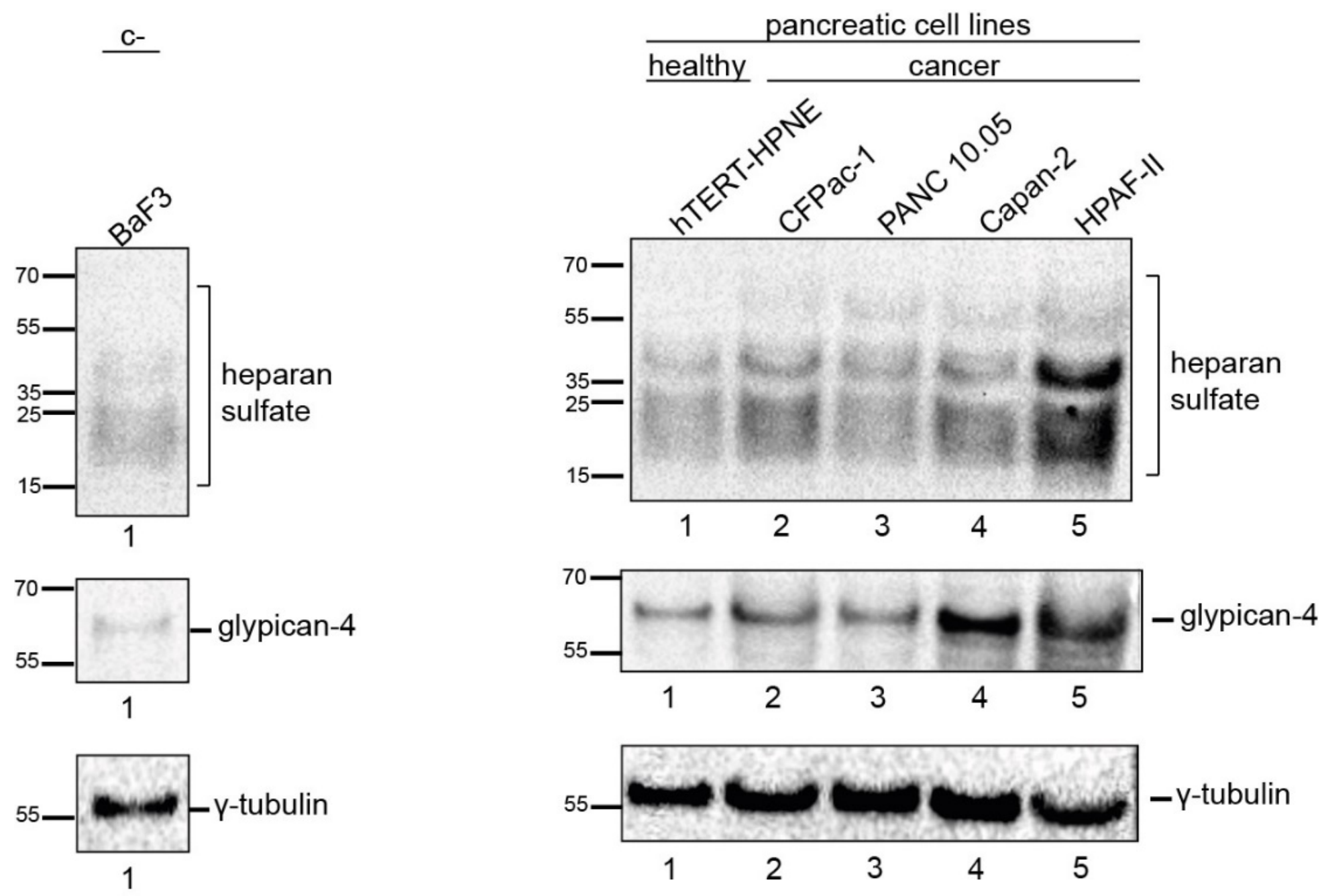

Supplementary Figure 15. HSPGs expression profile of selected cell lines using Western-Blot analysis. BaF3 cells were used as negative control (c-) due to lack of HS synthesis.

Supplementary Fig. 16

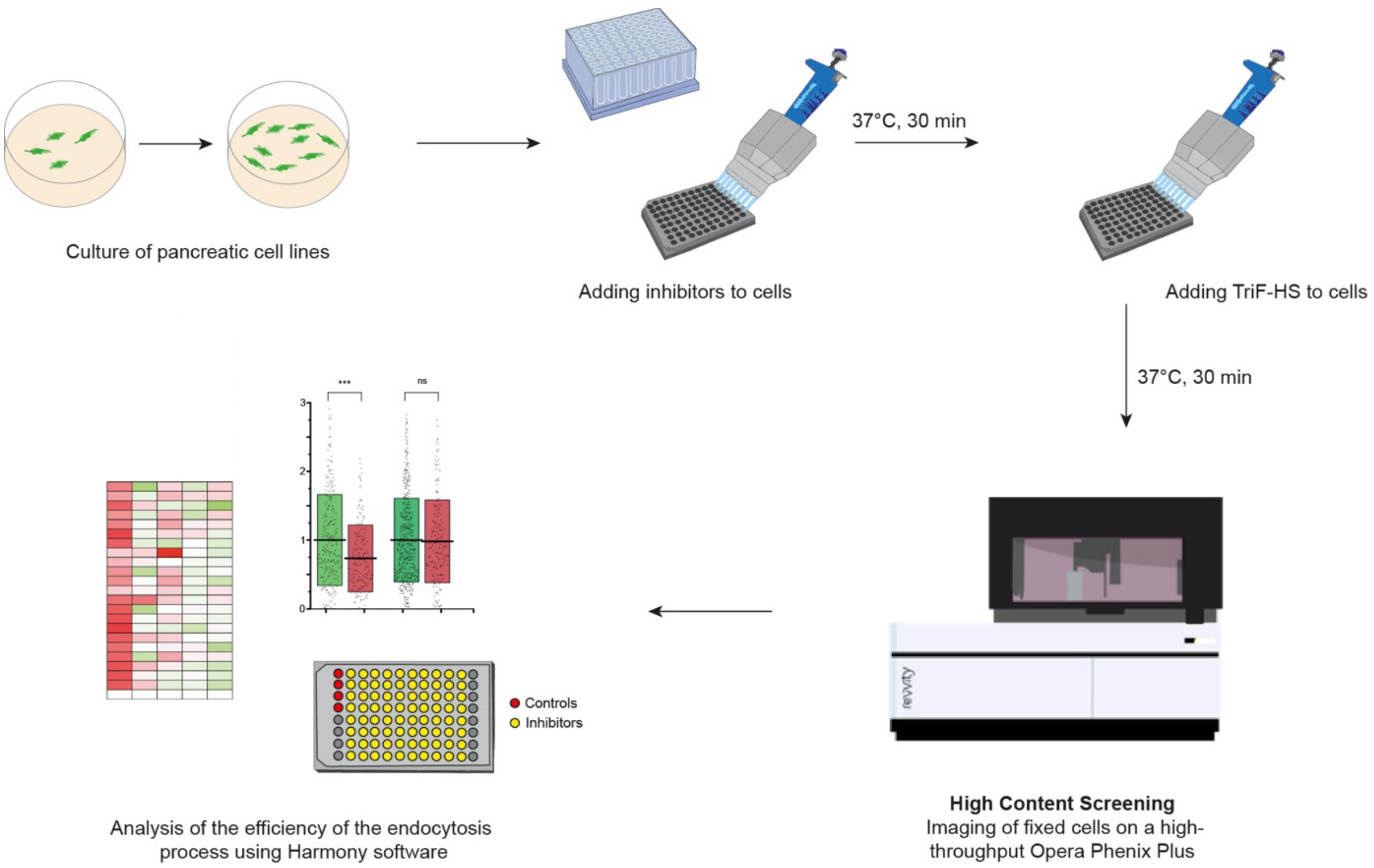

Supplementary Figure 16. Schematic representation of a high-content screening experiment aiming on identification of TriF$_{HS}$/HSPG ADE modulators. The efficiency of the endocytosis process was monitored qualitatively and quantitively using Opera Phenix Plus confocal microscopy. We designed and optimized an HCS experiment that used a library of 2,653 chemical compounds as inhibitors. Cells were seeded onto a microscope plate. Then cells were pre-treated with an inhibitor solution (one inhibitor per well) for 30 min at 37 °C; after such pretreatment, TriF$_{HS}$ was added to the cells. The cells were incubated for 30 min at 37 °C. After this time, cells were fixed and subjected to HSC confocal microscopy imaging and further qualitative and quantitative analysis with Harmony software.

Supplementary Fig. 17

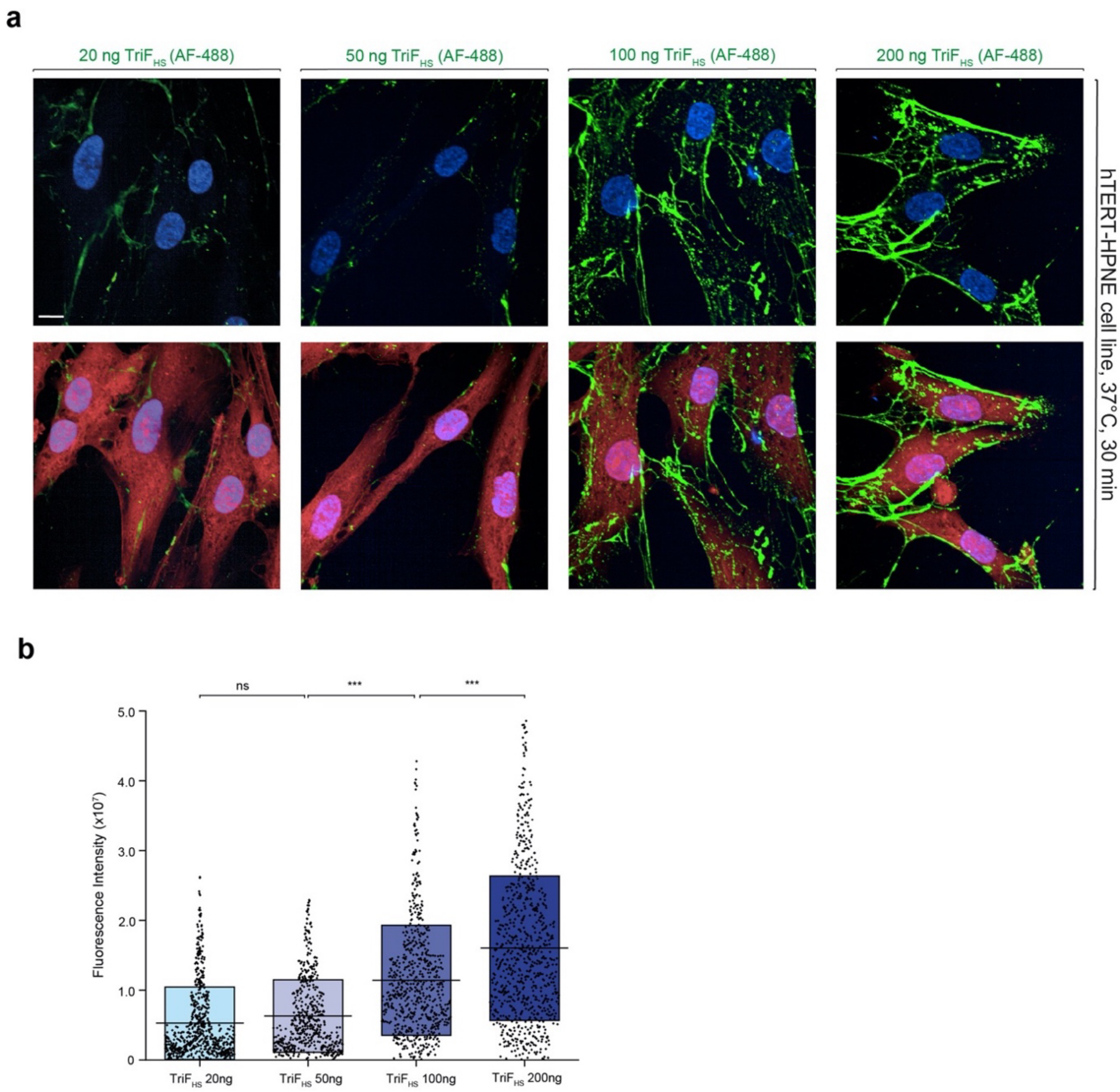

Supplementary Figure 17. Assessment of the concentration-dependence of TriF$_{HS}$ internalization into hTERT-HPNE cells. The efficiency of the endocytosis process was monitored using Opera Phenix Plus confocal microscopy. **A.** Microscopy images of hTERT-HPNE cells treated with different concentrations of TriF$_{HS}$ (Alexa 488) for 30 min at 37°C. Nuclei were stained using NucBlue Live and cytoplasm was stained using CellMask Deep Red. Scale bar represents 10 µm. **B.** Fluorescence intensity in Alexa 488 channel was measured in each cell (region defined by CellMask) and compared between different concentrations of TriF$_{HS}$. Each dot on the graph corresponds to the fluorescence intensity in one cell. For each group minimum 547 cells were analyzed, n=3. Statistical analyses were performed using analysis of variance with Tukey HSD test (*$p < 0.05$; **$p < 0.005$ and ***$p < 0.001$).

Supplementary Fig. 18

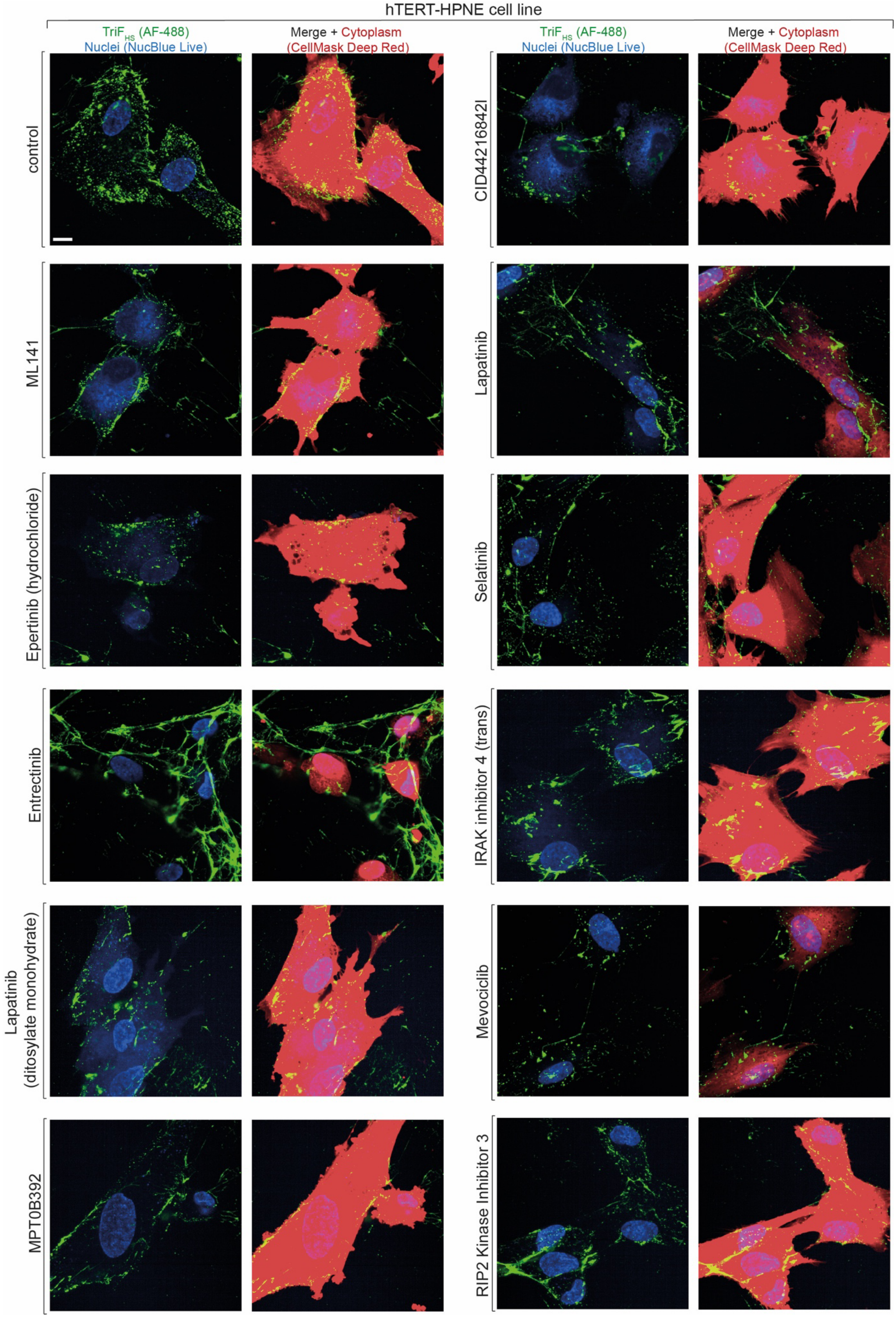

Supplementary Figure 18. Microscopy images of cells treated with compounds that significantly decrease TriF$_{HS}$ endocytosis to hTERT-HPNE cells (1/4). Microscopy images of hTERT-HPNE cells treated with TriF$_{HS}$ (Alexa 488) for 30 min at 37°C. Nuclei were stained using NucBlue Live and cytoplasm was stained using CellMask Deep Red. Scale bar represents 10 μm.

Supplementary Fig. 19

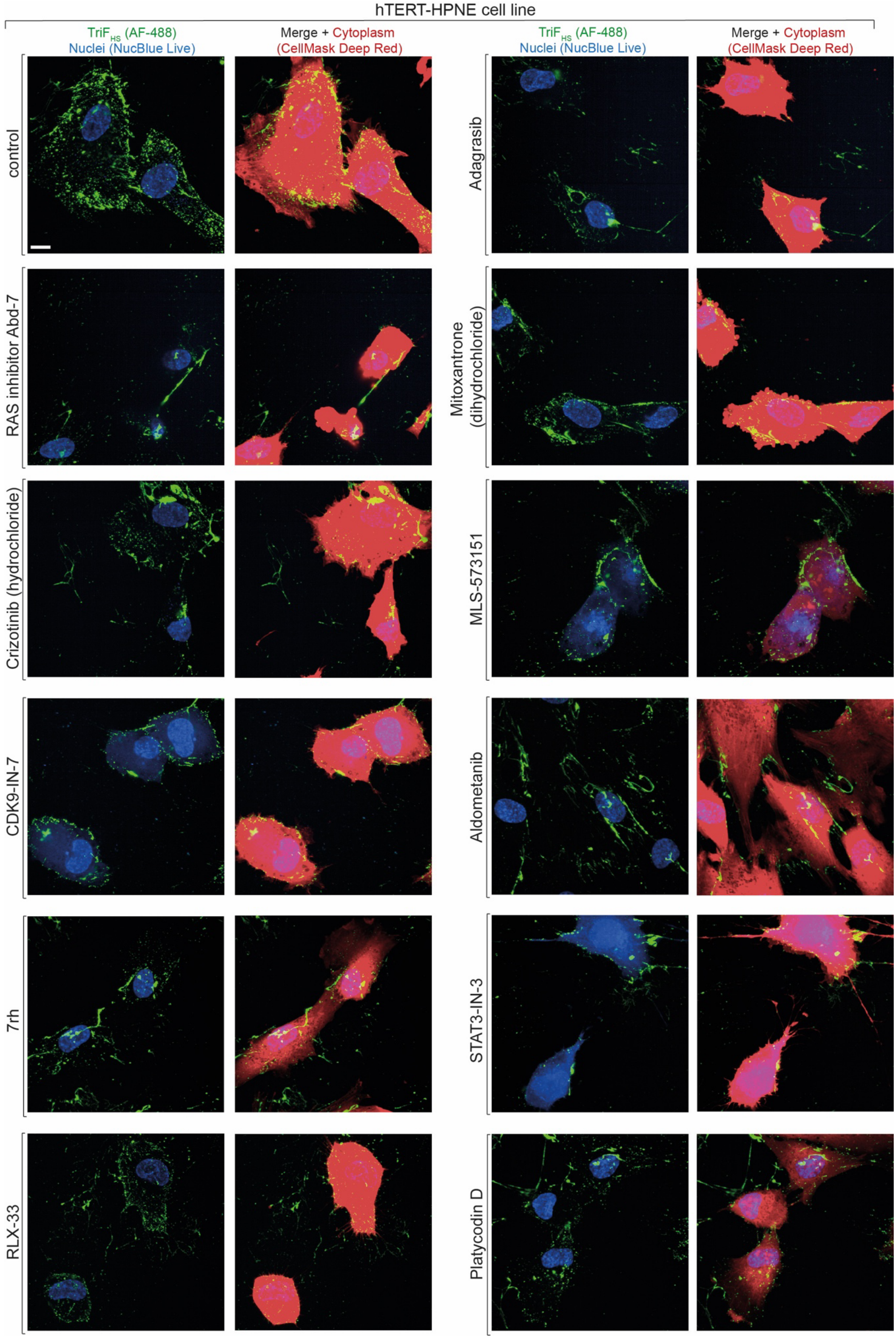

Supplementary Figure 19. Microscopy images of cells treated with compounds that significantly decrease TriF$_{HS}$ endocytosis to hTERT-HPNE cells (2/4). Microscopy images of hTERT-HPNE cells treated with TriF$_{HS}$ (Alexa 488) for 30 min at 37°C. Nuclei were stained using NucBlue Live and cytoplasm was stained using CellMask Deep Red. Scale bar represents 10 μm.

Supplementary Fig. 20

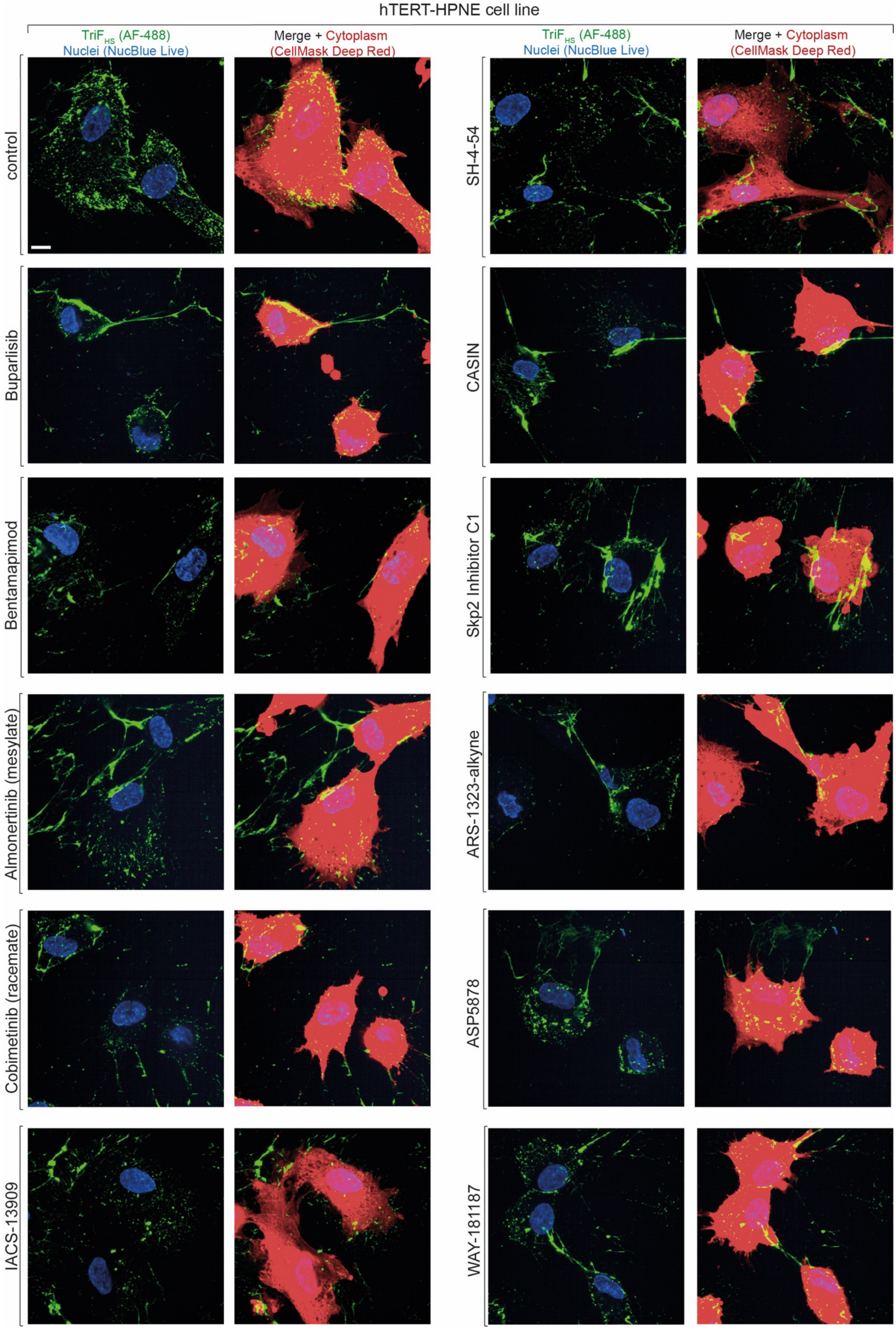

Supplementary Figure 20. Microscopy images of cells treated with compounds that significantly decrease $TriF_{HS}$ endocytosis to hTERT-HPNE cells (3/4). Microscopy images of hTERT-HPNE cells treated with $TriF_{HS}$ (Alexa 488) for 30 min at 37°C. Nuclei were stained using NucBlue Live and cytoplasm was stained using CellMask Deep Red. Scale bar represents 10 μm.

Supplementary Fig. 21

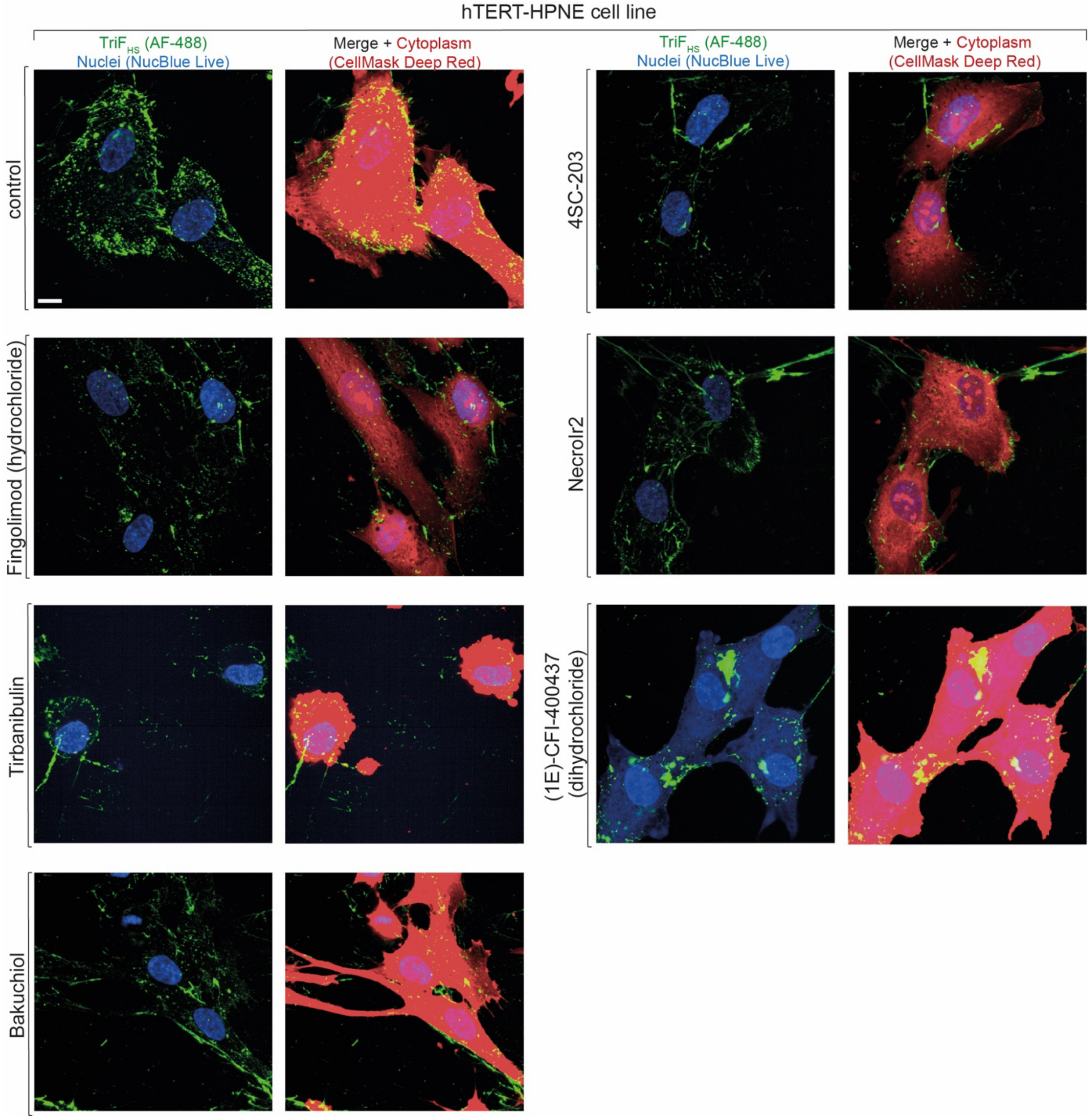

Supplementary Figure 21. Microscopy images of cells treated with compounds that significantly decrease TriF$_{HS}$ endocytosis to hTERT-HPNE cells (4/4). Microscopy images of hTERT-HPNE cells treated with TriF$_{HS}$ (Alexa 488) for 30 min at 37°C. Nuclei were stained using NucBlue Live and cytoplasm was stained using CellMask Deep Red. Scale bar represents 10 µm.

Supplementary Fig. 22

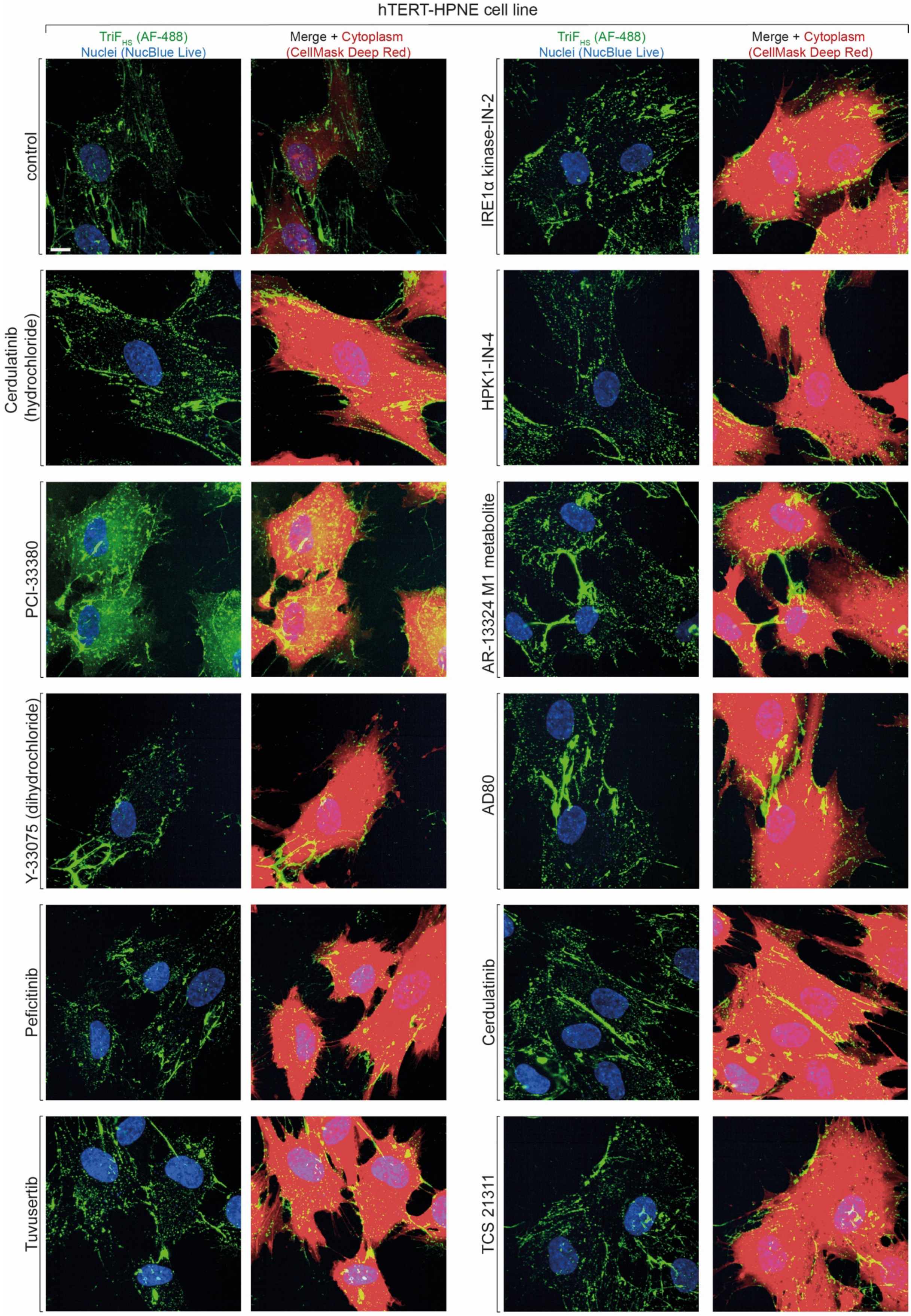

Supplementary Figure 22. Microscopy images of cells treated with compounds that significantly increase $TriF_{HS}$ endocytosis to hTERT-HPNE cells (1/4). Microscopy images of hTERT-HPNE cells treated with $TriF_{HS}$ (Alexa 488) for 30 min at 37°C. Nuclei were stained using NucBlue Live and cytoplasm was stained using CellMask Deep Red. Scale bar represents 10 μm.

Supplementary Fig. 23

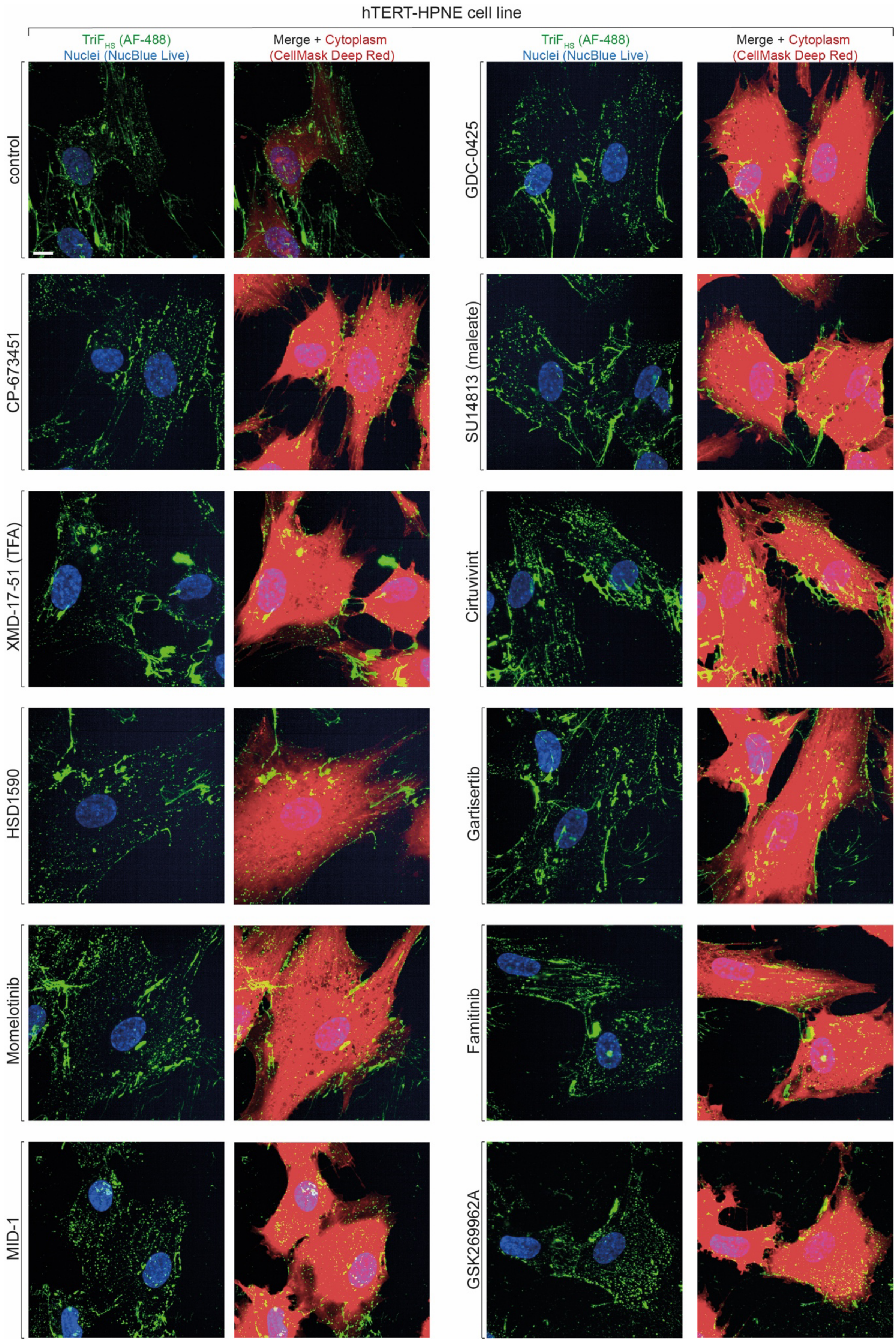

Supplementary Figure 23. Microscopy images of cells treated with compounds that significantly increase $TriF_{HS}$ endocytosis to hTERT-HPNE cells (2/4). Microscopy images of hTERT-HPNE cells treated with $TriF_{HS}$ (Alexa 488) for 30 min at 37°C. Nuclei were stained using NucBlue Live and cytoplasm was stained using CellMask Deep Red. Scale bar represents 10 µm.

Supplementary Fig. 24

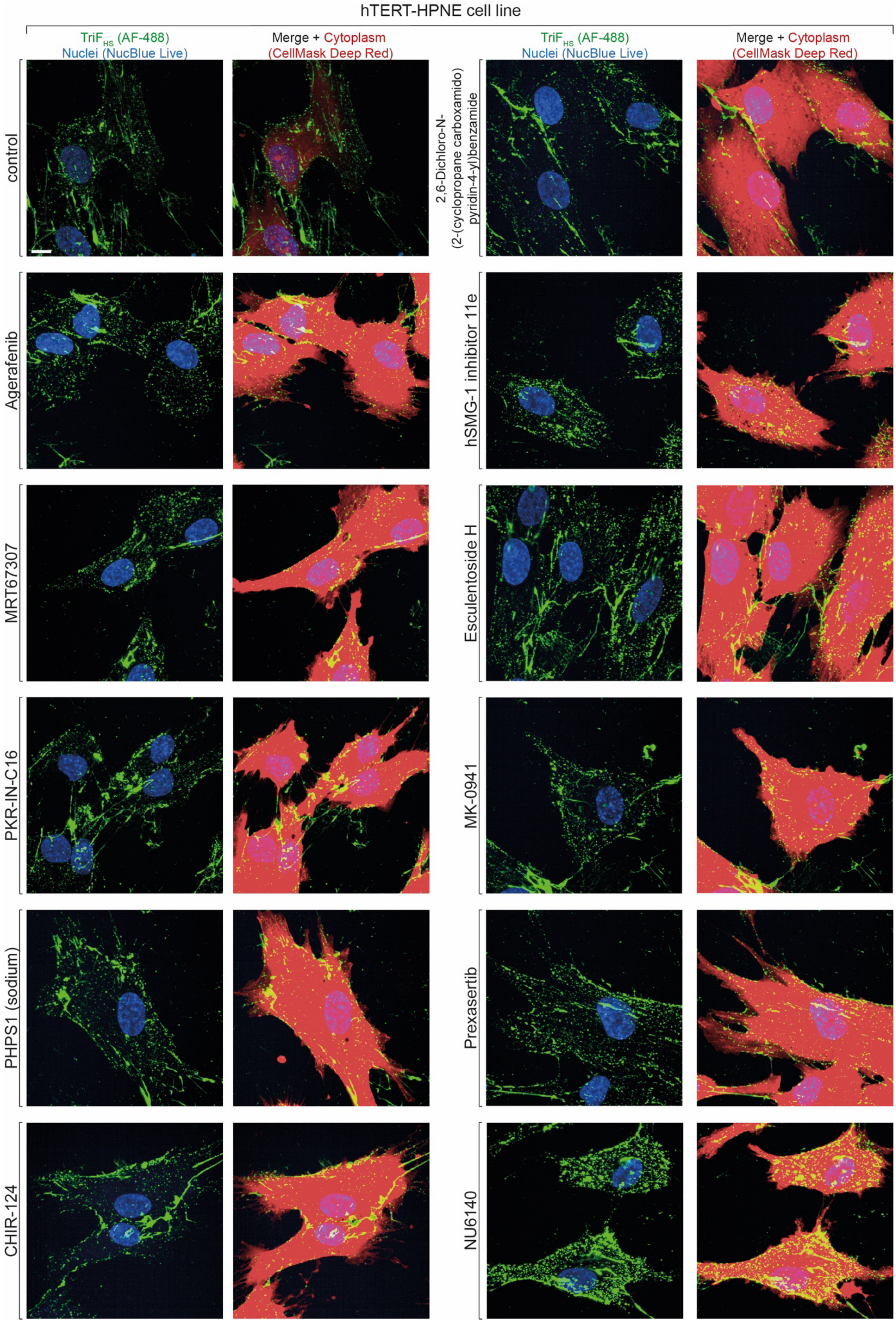

Supplementary Figure 24. Microscopy images of cells treated with compounds that significantly increase $TriF_{HS}$ endocytosis to hTERT-HPNE cells (3/4). Microscopy images of hTERT-HPNE cells treated with $TriF_{HS}$ (Alexa 488) for 30 min at 37°C. Nuclei were stained using NucBlue Live and cytoplasm was stained using CellMask Deep Red. Scale bar represents 10 μm.

Supplementary Fig. 25

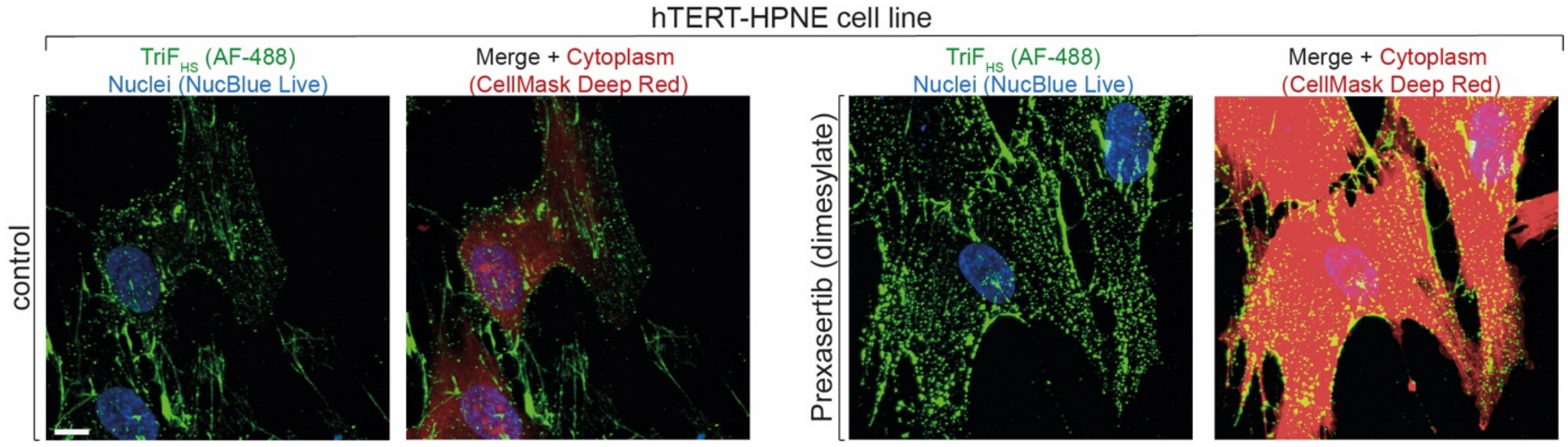

Supplementary Figure 25. Microscopy images of cells treated with compounds that significantly increase TriF$_{HS}$ endocytosis to hTERT-HPNE cells (4/4). Microscopy images of hTERT-HPNE cells treated with TriF$_{HS}$ (Alexa 488) for 30 min at 37°C. Nuclei were stained using NucBlue Live and cytoplasm was stained using CellMask Deep Red. Scale bar represents 10 µm.

Supplementary Fig. 26

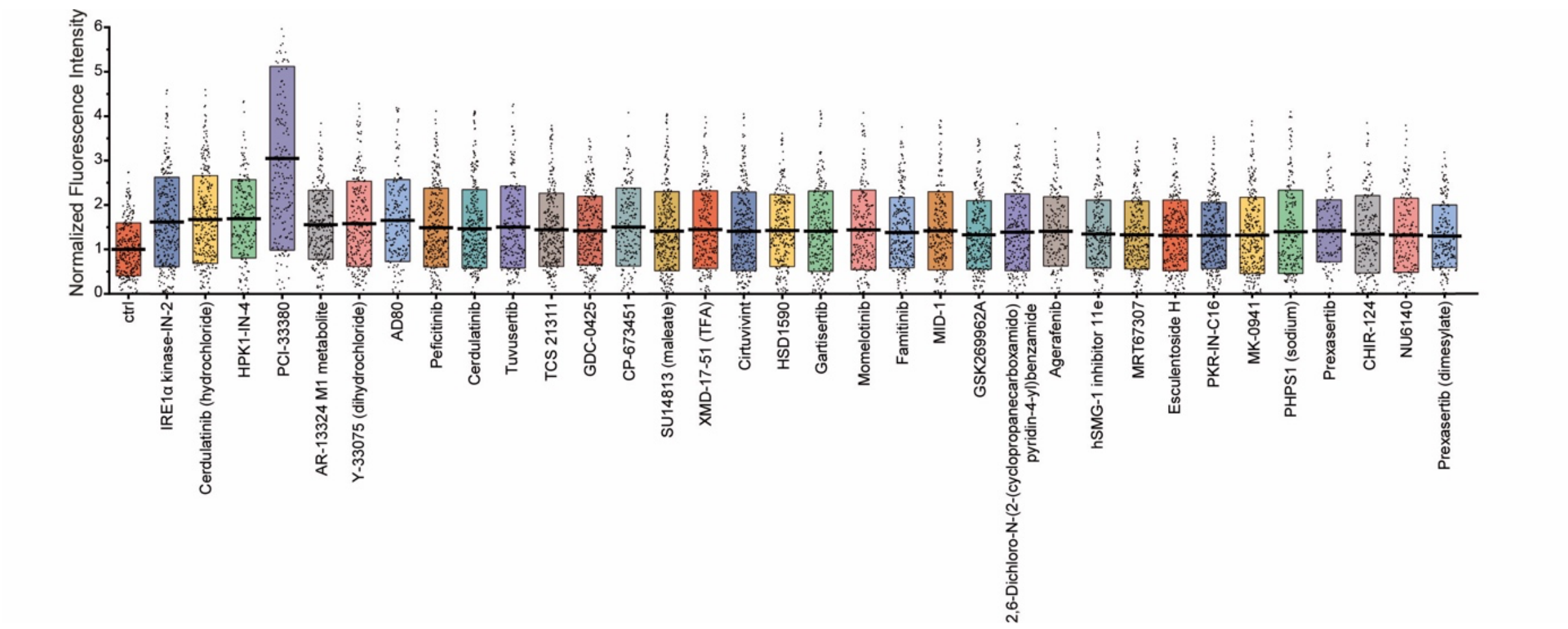

Supplementary Figure 26. Compounds that statistically significantly enhance TriF$_{HS}$ endocytosis to hTERT-HPNE cells. HCS screening of the library identified 34 compounds that statistically significantly upregulate TriF$_{HS}$ internalization. Intracellular fluorescence intensity in Alexa 488 channel (representing TriF$_{HS}$ inherent fluorescence) was measured in individual cells (region defined by CellMask). Each dot on the graph represents the fluorescence intensity in one cell. For each group minimum 184 cells were analyzed, n=3. Statistical analyses were performed using analysis of variance with Tukey HSD test (*$p < 0.05$; **$p < 0.005$ and ***$p < 0.001$). Statistical analyses were performed for at least three independent experiments.

Supplementary Fig. 27

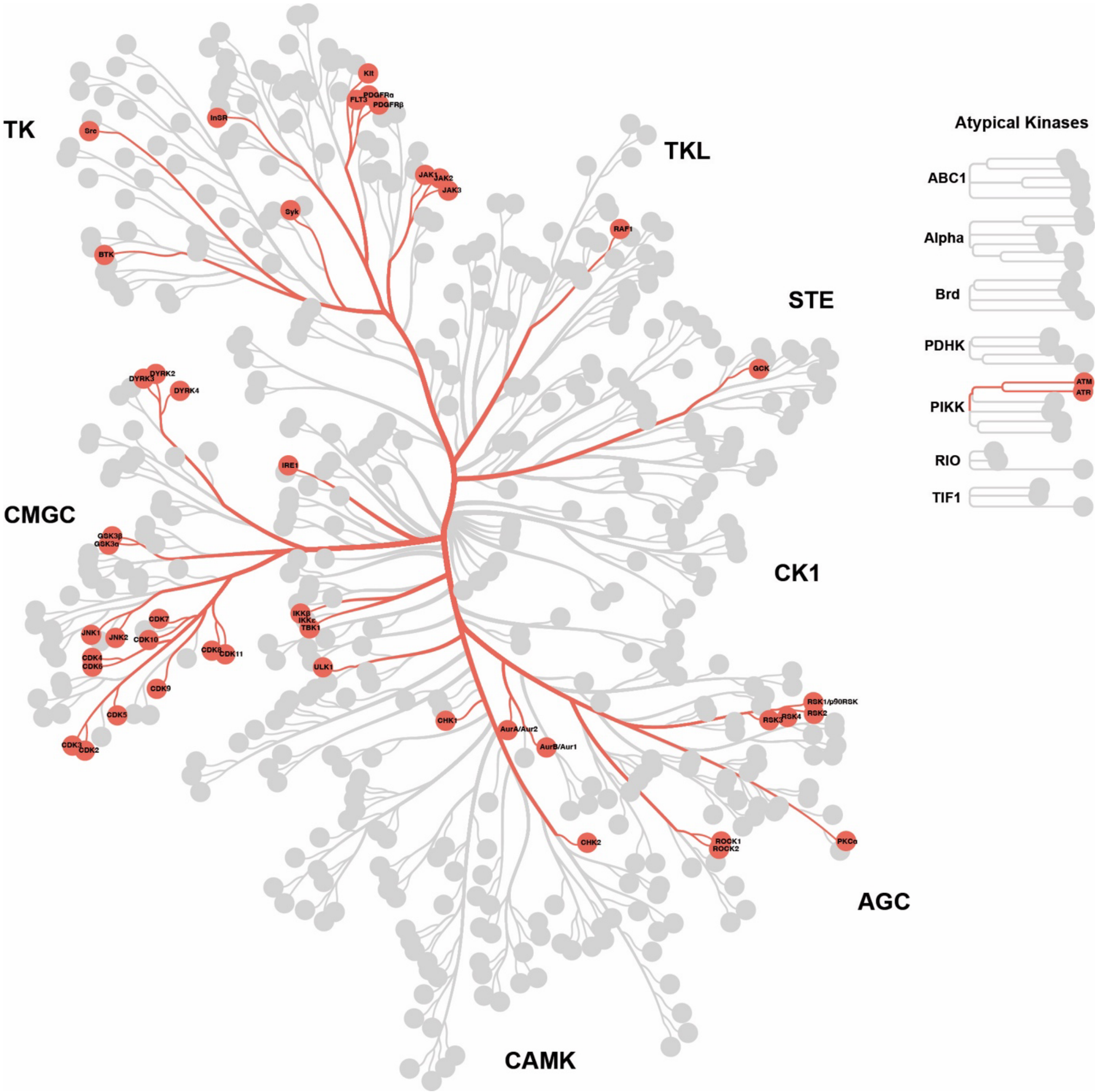

Supplementary Figure 27. Signaling pathways whose inhibition leads to increased TriF$_{HS}$ endocytosis to hTERT-HPNE cells. Kinase tree analysis of the targets (red dots) of selected activators. Graph was made in CORAL application (http://phanstiel-lab.med.unc.edu/CORAL/). The CMGC family of kinases appears to play a predominant role in negatively regulating the cellular uptake of TriF$_{HS}$.

Supplementary Fig. 28

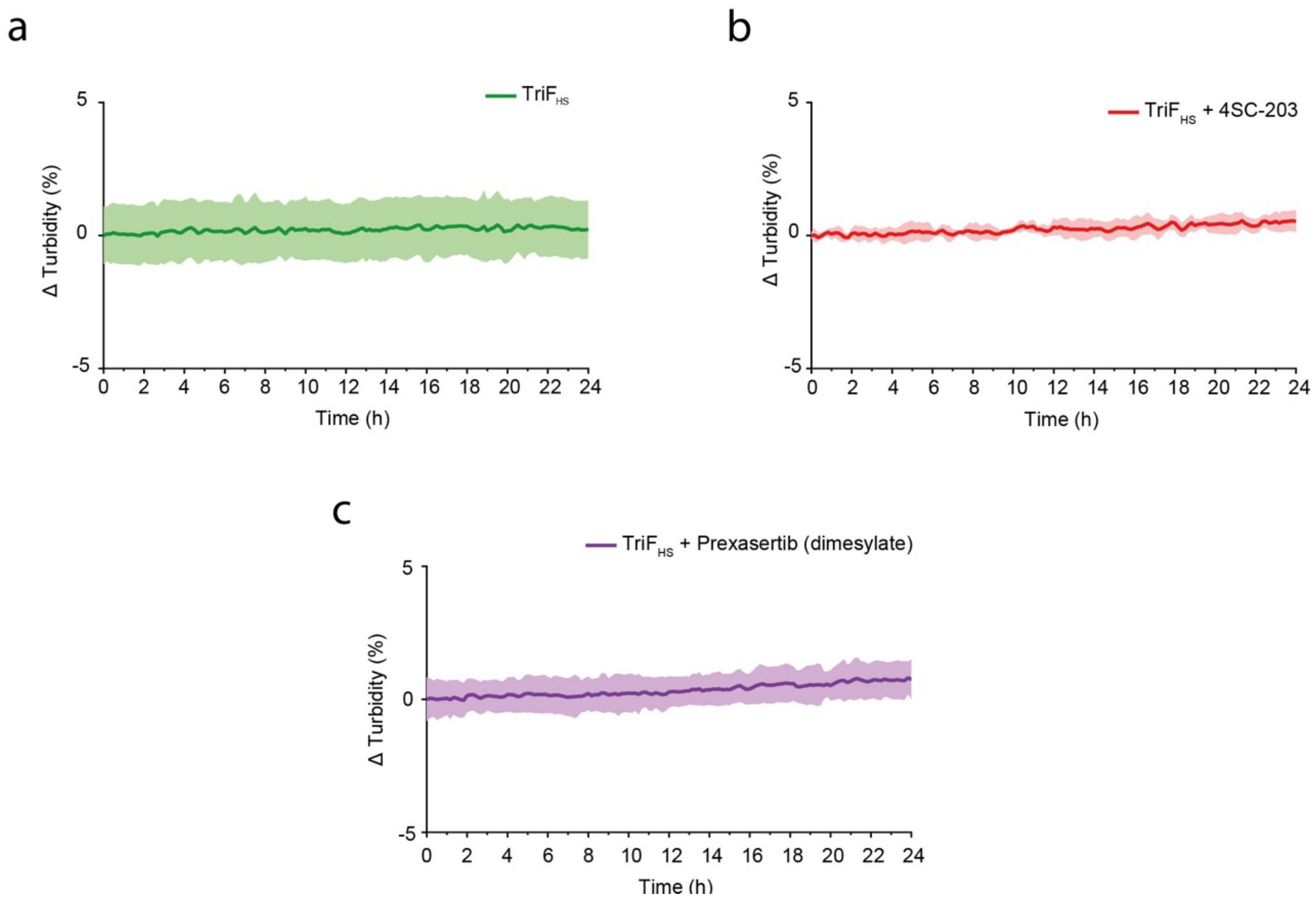

Supplementary Figure 28. Turbidity of $TriF_{HS}$ over 24h in presence or absence of selected ADE modulators. a. $TriF_{HS}$. b. $TriF_{HS}$ + 4SC-203. c. $TriF_{HS}$ + Prexasertib (dimesylate).

Supplementary Fig. 29

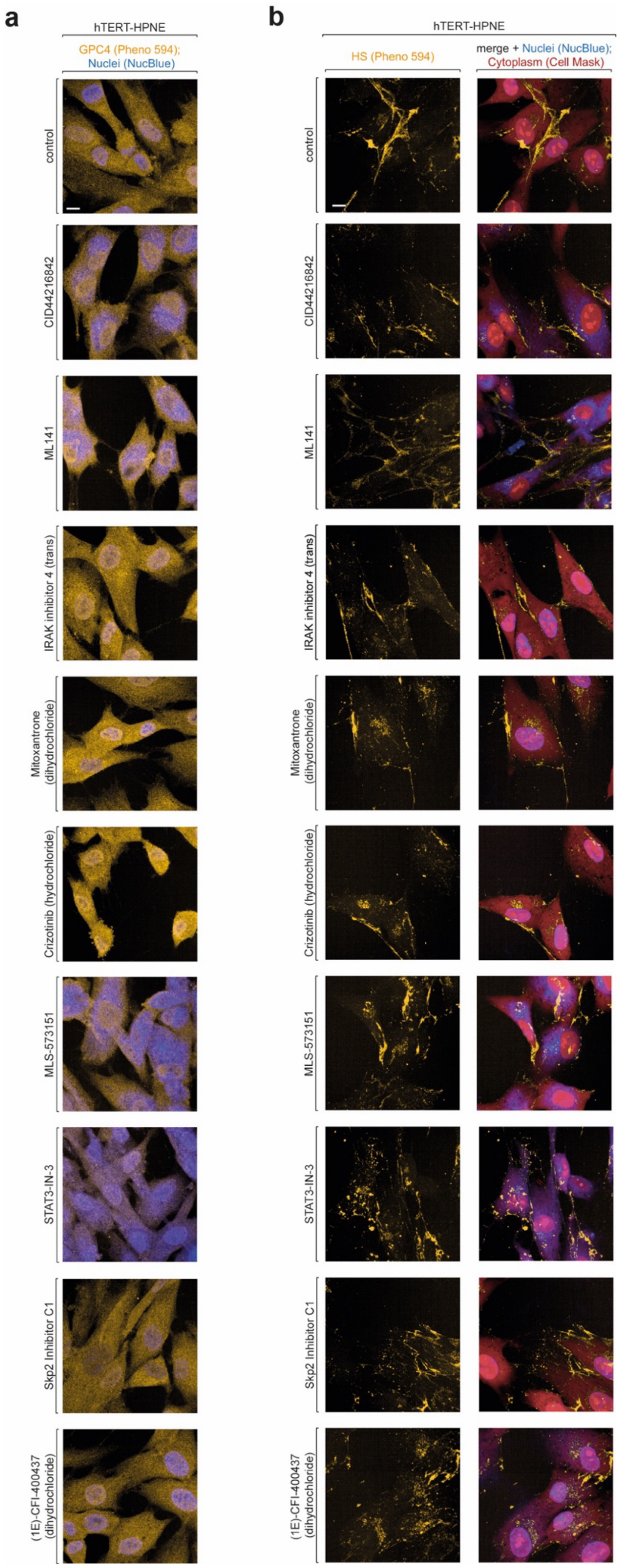

Supplementary Figure 29. Microscopy images of hTERT-HPNE cells treated with model inhibitors that significantly decrease TriF$_{HS}$ endocytosis. hTERT-HPNE cells were treated with one of the nine identified compounds for 30 min at 37°C and subsequently fixed. a) HS (Pheno 594) and b) GPC4 (Pheno 594) were detected by immunolabeling. Nuclei were stained using NucBlue Live and cytoplasm was stained using CellMask Deep Red. Scale bar represents 10 µm.

Supplementary Fig. 30

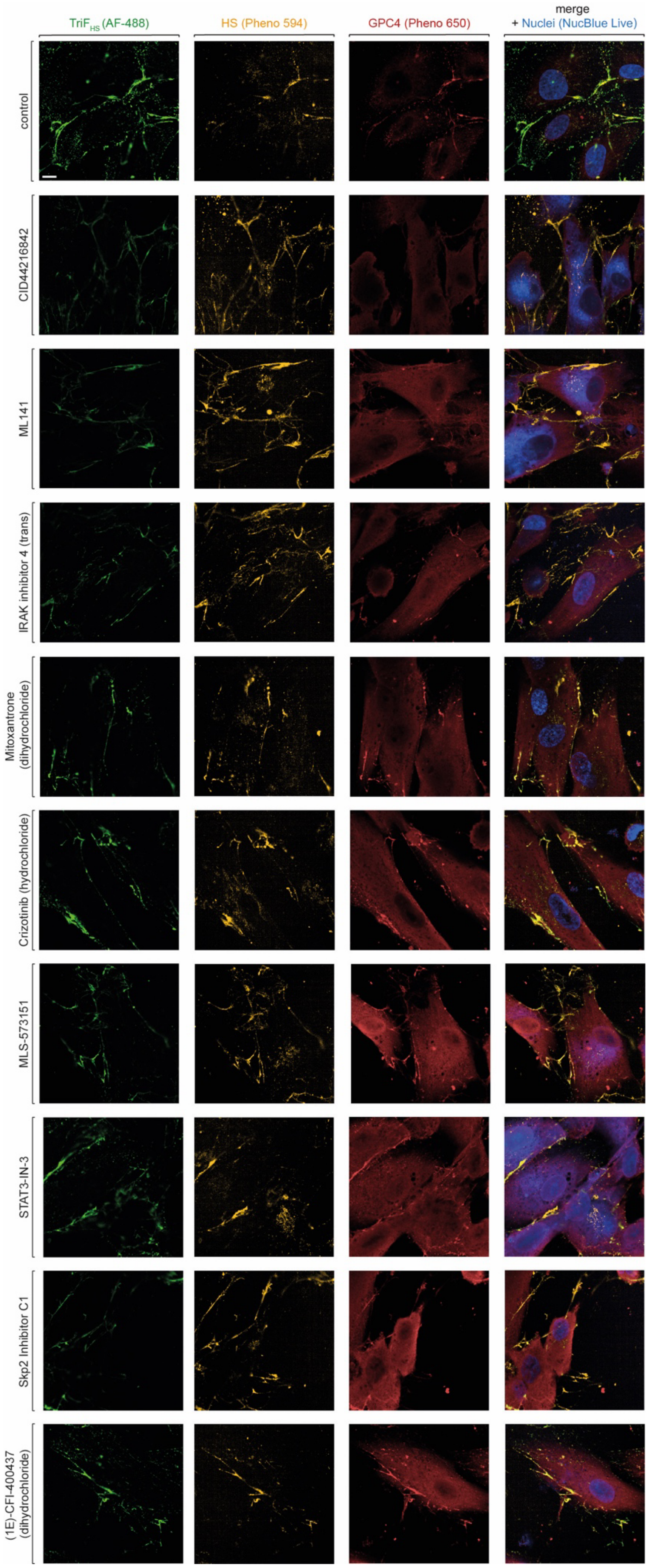

Supplementary Figure 30. Co-localization of TriF$_{HS}$ with HS and GPC4 in hTERT-HPNE cells upon treatment with model identified inhibitors. hTERT-HPNE cells were pretreated with the nine model inhibitors for 30 min at 37°C, followed by the addition of TriF$_{HS}$ and further incubation for 30 min at 37°C. The cells were then fixed. Heparan Sulfate (Pheno 594) and GPC4 (Pheno 594) were detected by immunolabeling. Nuclei were stained using NucBlue Live. Scale bar represents 10 μm.

Supplementary Fig. 31

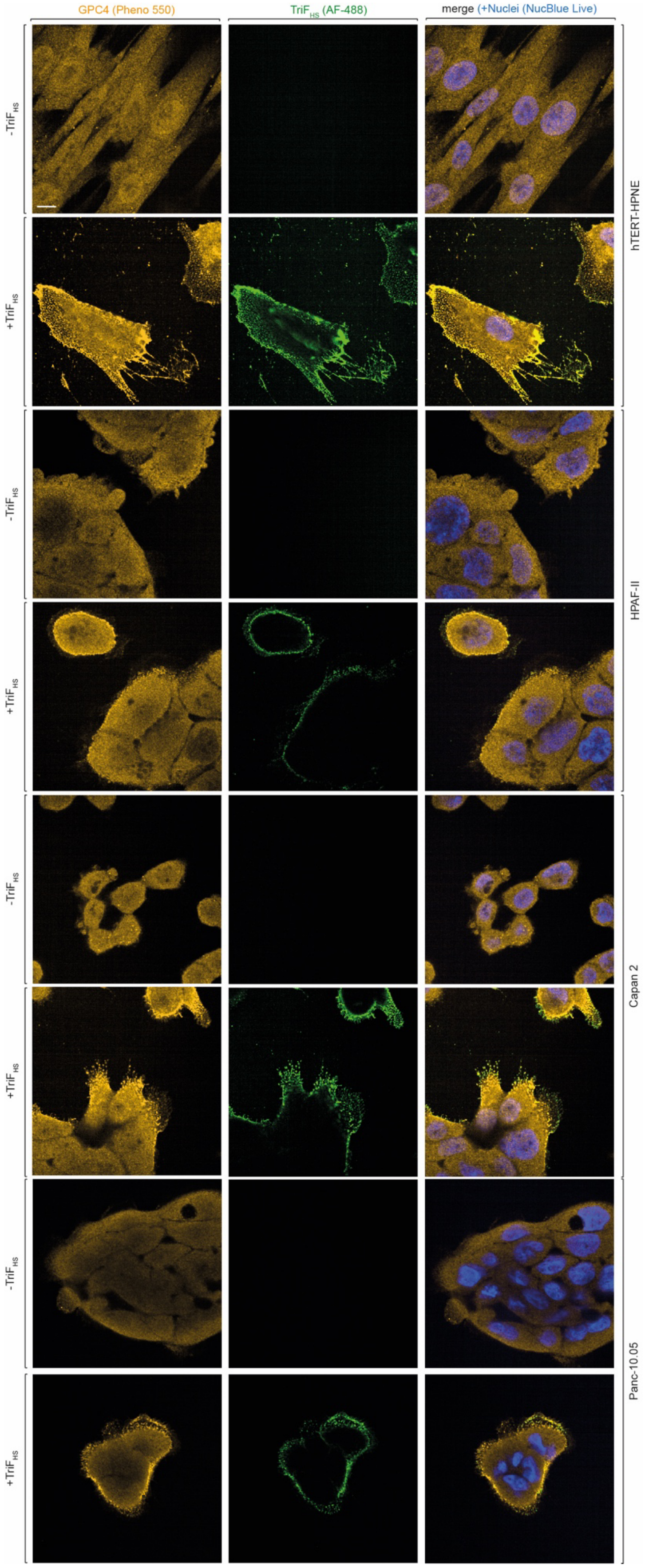

Supplementary Figure 31. Microscopy images of TriF$_{HS}$-mediated clustering of HSPGs on the surface of pancreatic cells. hTERT-HPNE, HPAF-II, Capan-2 and Panc-10.05 cells were treated with TriF$_{HS}$ or left untreated (control) for 30 min at 4°C, following with fixation. GPC4 (Pheno 594) was detected by immunolabeling. Nuclei were stained using NucBlue Live. Scale bar represents 10 μm.

Supplementary Fig. 32

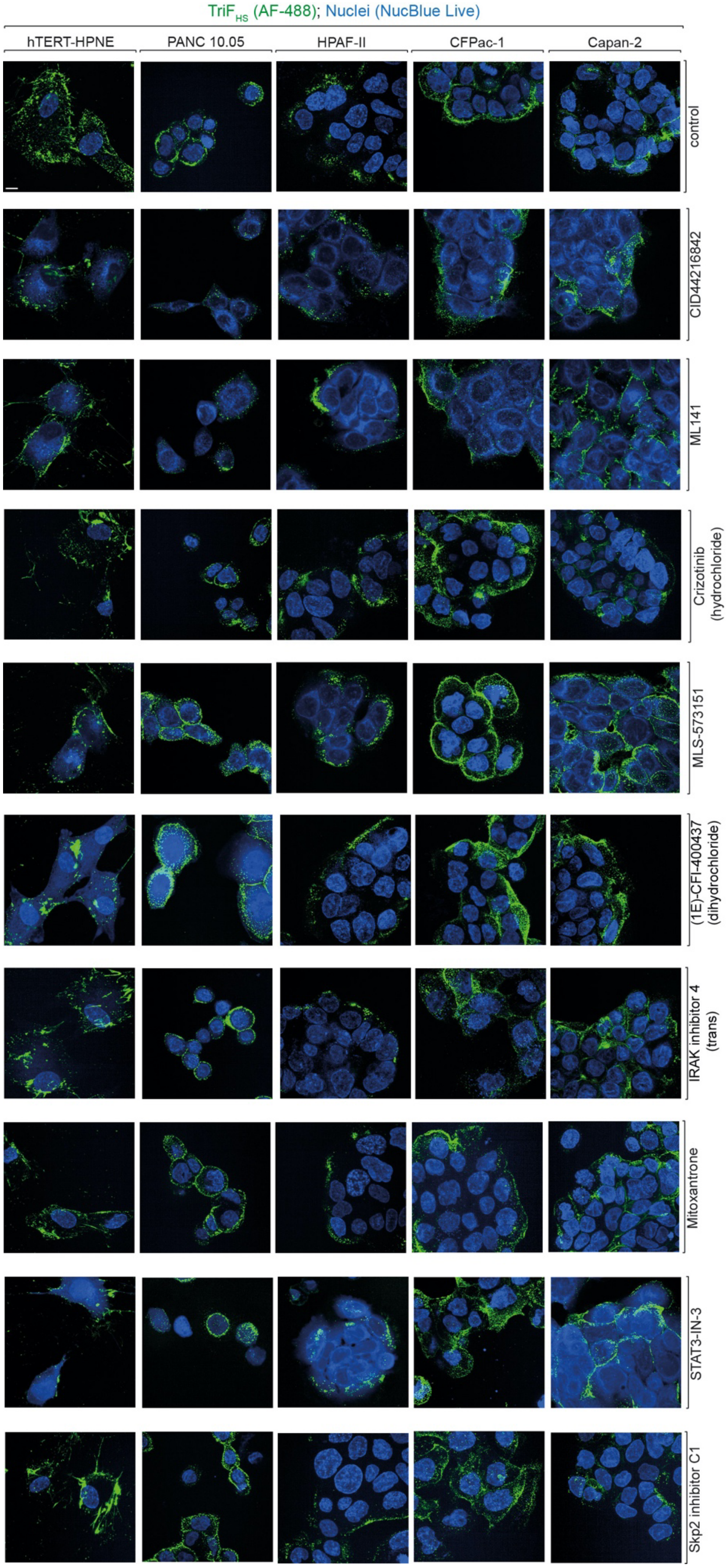

Supplementary Figure 32. The impact of nine compounds selected in the second HCS screen on endocytosis of TriF$_{HS}$. Microscopy images of cells treated with nine selected compounds that significantly inhibit TriF$_{HS}$ internalization to normal cells (hTERT-HPNE cell line), as well as have no impact or significantly promote internalization into pancreatic cancer cells (PANC 10.05, HPAF-II; CFPac-I; Capan-2 cell lines). Cells were pretreated with the indicated inhibitor for 30 minutes at 37°C, followed by the addition of TriF$_{HS}$ and further incubation for 30 minutes at 37°C. Nuclei were stained using NucBlue Live. Cells were fixed and analyzed with confocal microscopy. Scale bar represents 10 μm.

Supplementary Fig. 33

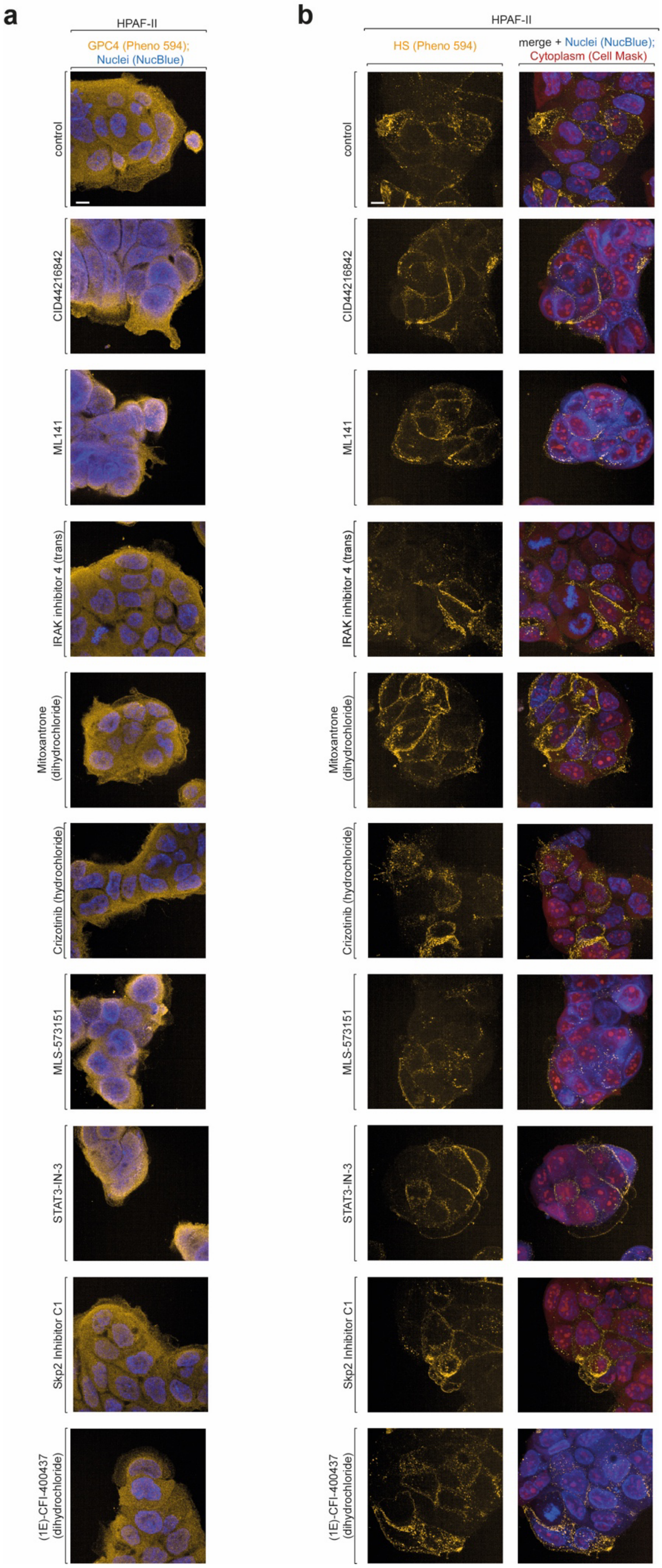

Supplementary Figure 33. Microscopy images of HPAF-II cells treated with nine model inhibitors. HPAF-II cells were treated with nine HCS-identified compounds (2nd screen) for 30 min in 37°C and subsequently fixed. a) HS (Pheno 594) and b) GPC4 (Pheno 594) were detected by immunolabeling. Nuclei were stained using NucBlue Live and cytoplasm was stained using CellMask Deep Red. Scale bar represents 10 μm.

Supplementary Fig. 34

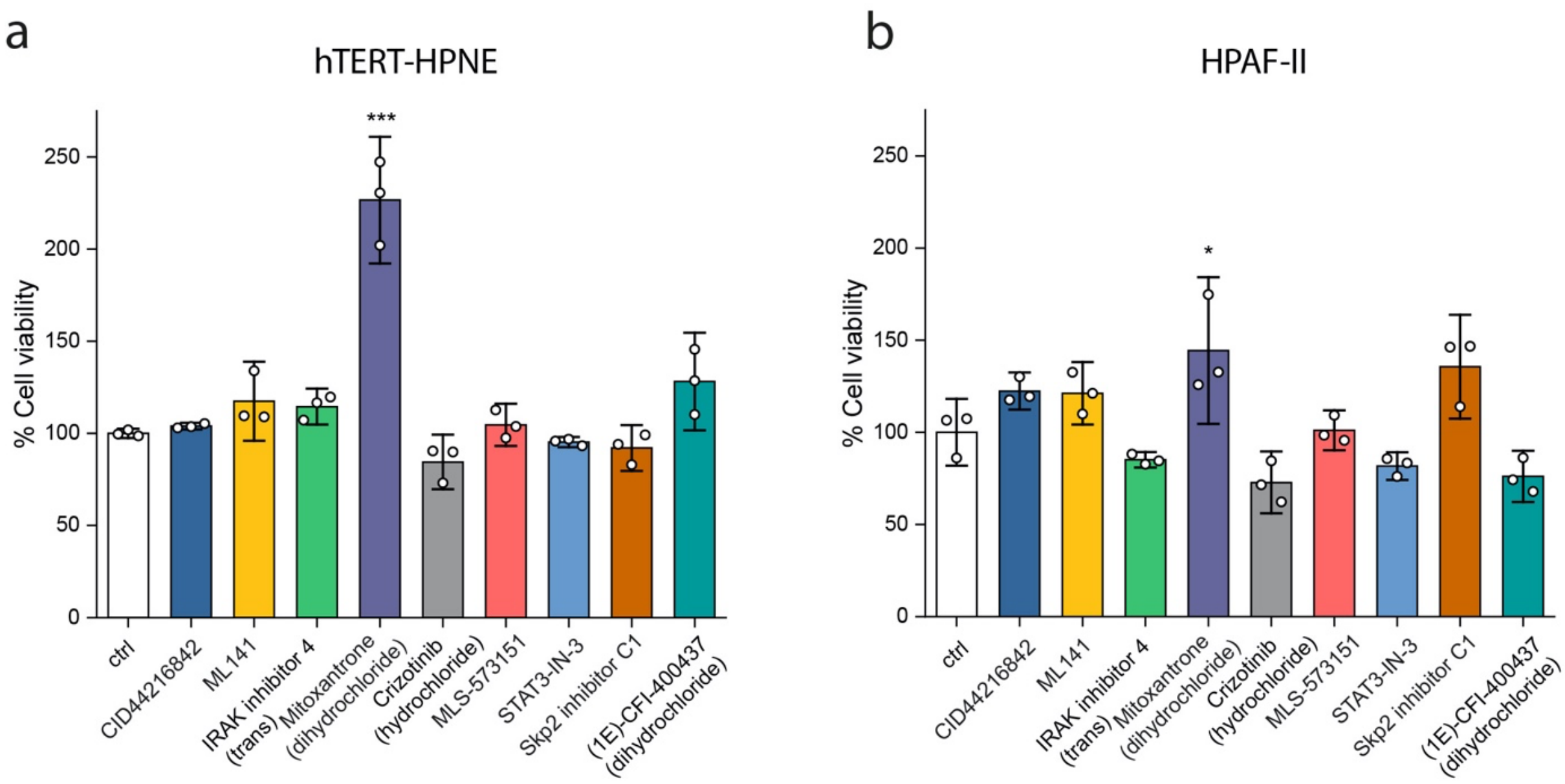

Supplementary Figure 34. Cell viability of healthy (hTERT-HPNE) and cancer (HPAF-II) pancreatic cells treated with nine HCS-selected (2nd HCS screen) inhibitors. a. hTERT-HPNE % cell viability. b. HPAF-II % cell viability. Each dot on the graph corresponds to the mean of three independent experiments. Results were normalized as 100% cell viability of control cells. Statistical analyses were conducted using one-way ANOVA, followed by post-hoc tests using the Spjøtvoll-Stoline modified Tukey HSD test. Significance levels were indicated as follows: *$p < 0.05$, **$p < 0.005$, ***$p < 0.001$. n=3. Statistical analyses were performed for at least three independent experiments.

Supplementary Fig. 35

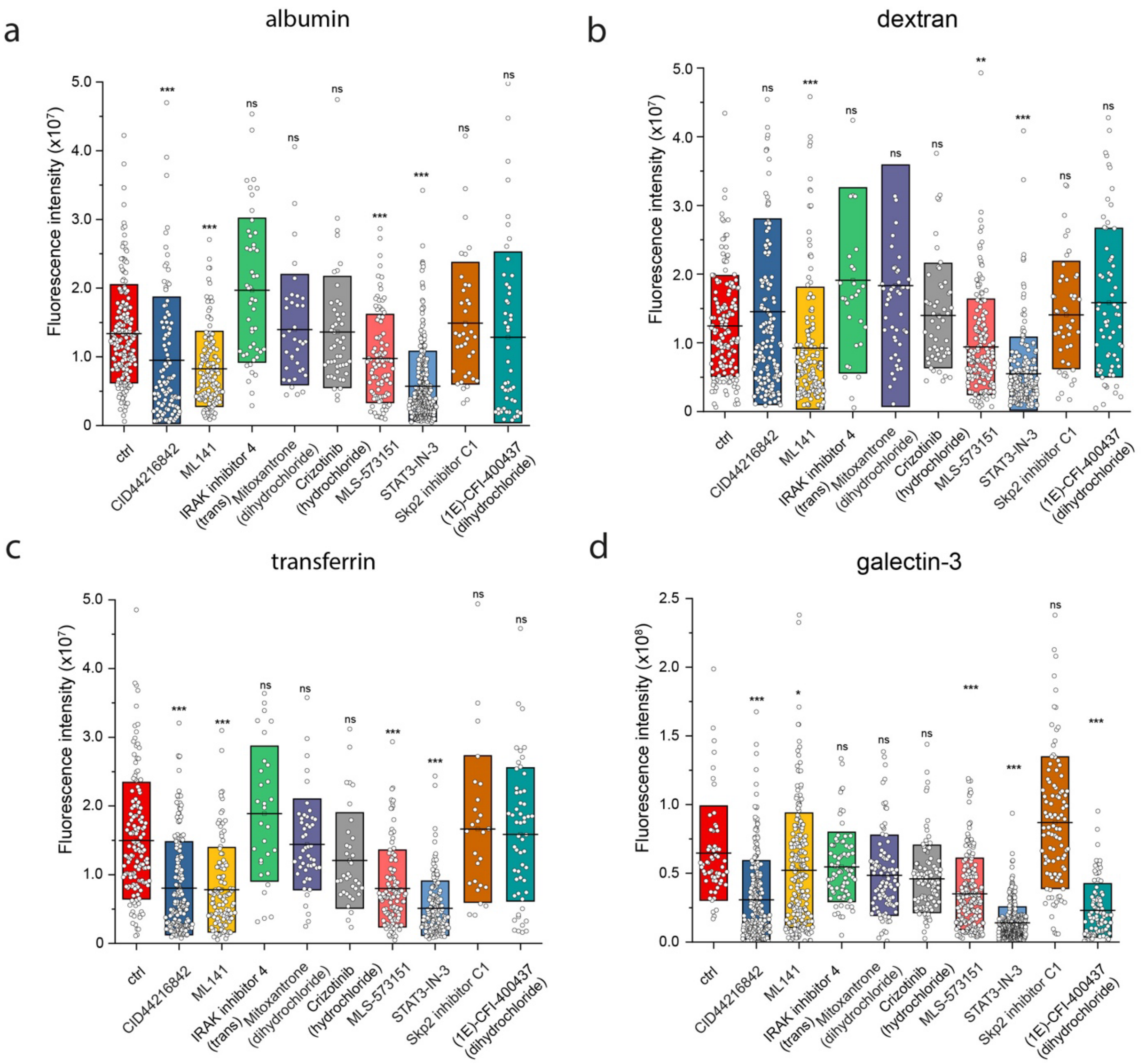

Supplementary Figure 35. Endocytosis of fluorescently labelled model cargo proteins to hTERT-HPNE pancreatic cells treated with nine HCS-selected (2nd HCS screen) inhibitors. a. endocytosis of albumin. b. endocytosis of dextran. c. endocytosis of transferrin. d. endocytosis of galectin-3. Statistical analyses were conducted using one-way ANOVA, followed by post-hoc tests using the Kruskal-Wallis test, n=3. Significance levels were indicated as follows: $*p < 0.05$, $**p < 0.005$, $***p < 0.001$.

Supplementary Fig. 36

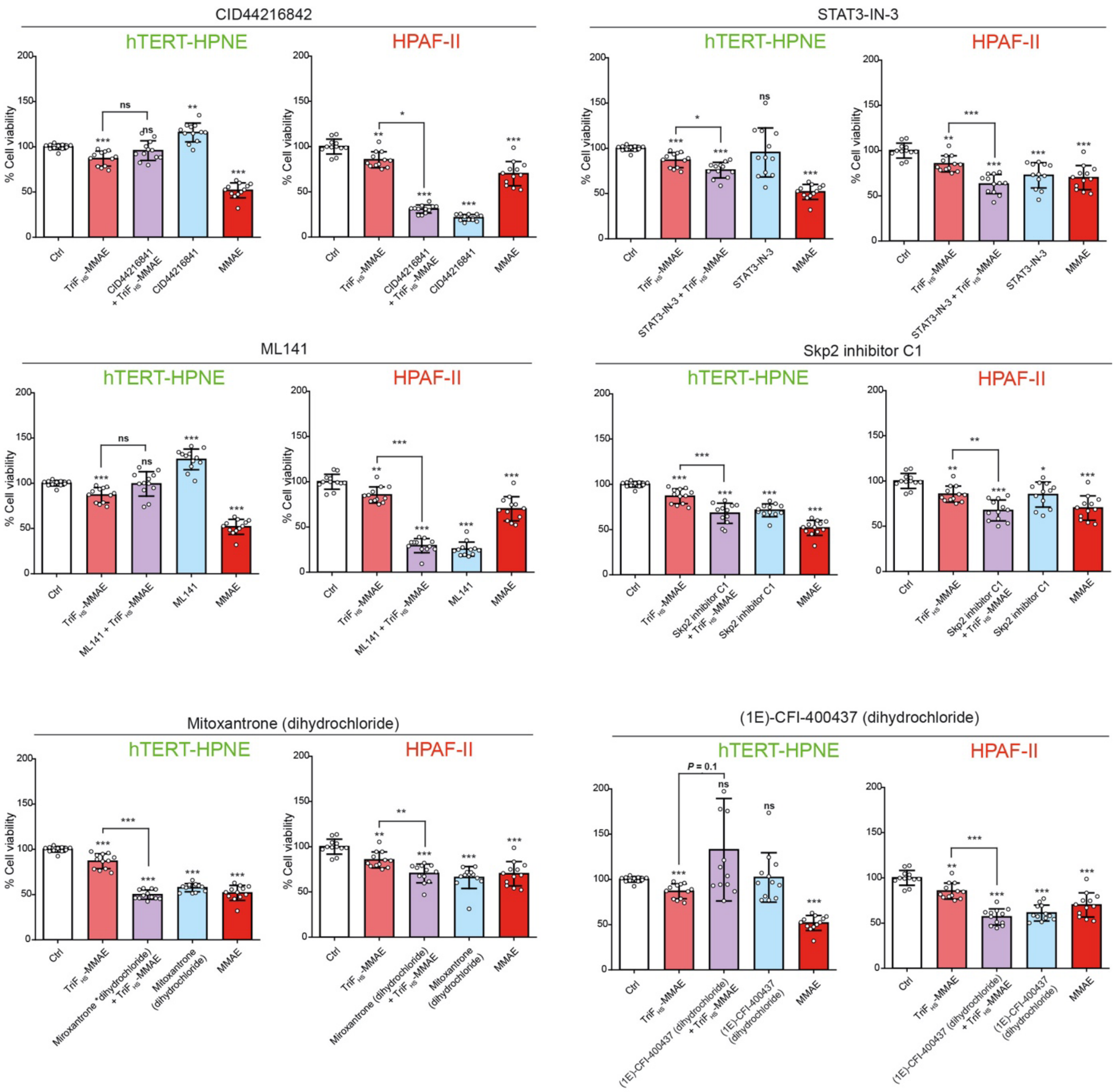

Supplementary Figure 36. ADE modulators that turned out to be toxic for hTERT-HPNE cells. Comparison of impact of different kinase inhibitors on viability of hTERT-HPNE and HPAF-II cell lines. Each dot on the graph corresponds to the one cell. Ctrl – cells untreated; $TriF_{HS}$-MMAE – cells treated with $TriF_{HS}$-MMAE; Name of compound + $TriF_{HS}$-MMAE – cells pre-treated with compound and then treated with $TriF_{HS}$-MMAE; MMAE – cells treated with MMAE. Results were normalized as 100% cell viability of control cells. Statistical analyses were performed using analysis of variance with Tukey HSD test or the Games-Howell test c n=3. Statistical analyses were performed for at least three independent experiments.

Supplementary Fig. 37

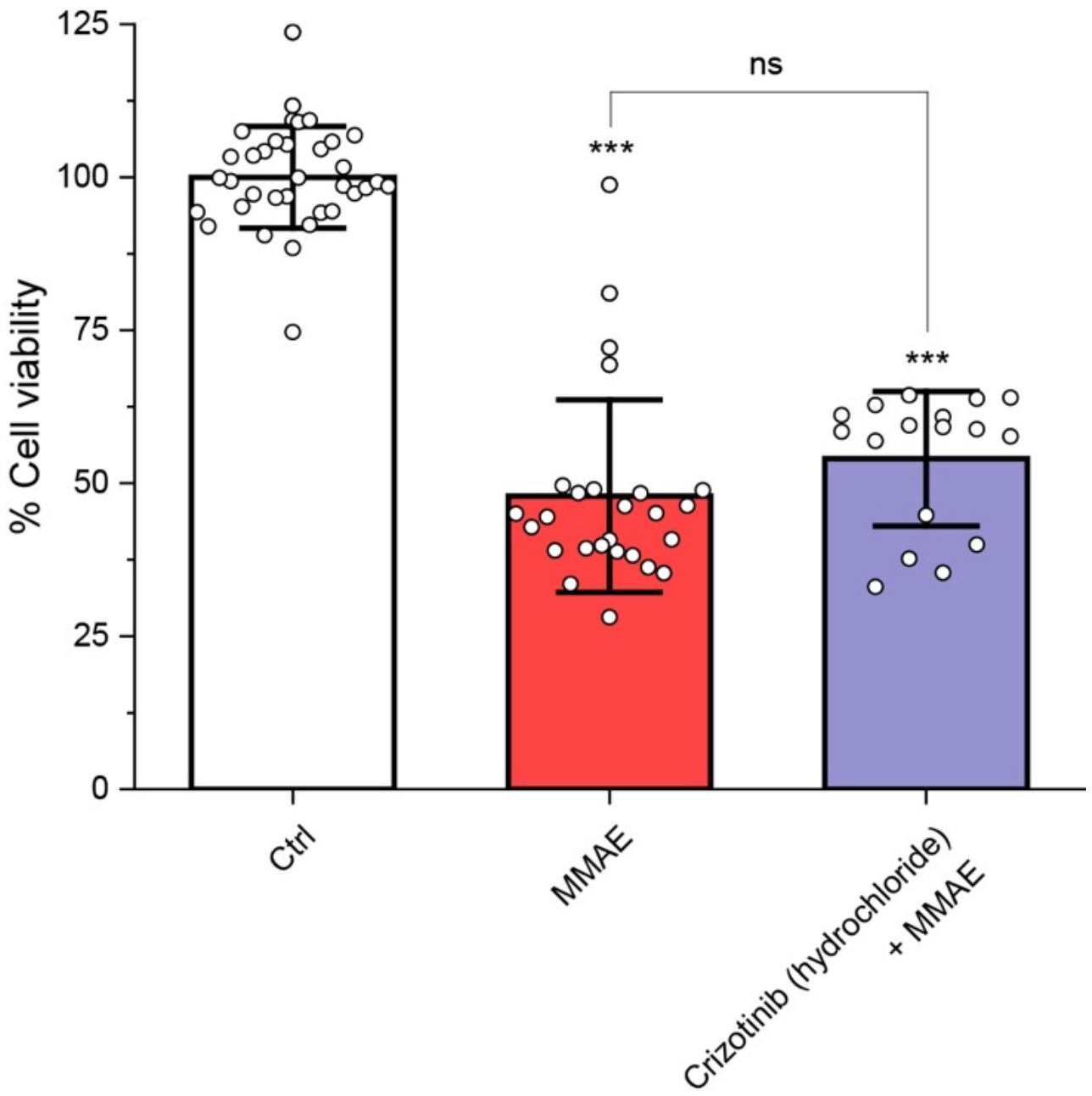

Supplementary Figure 37. hTERT-HPNE cells viability treated with MMAE, in presence or absence of Crizotinib (hydrochloride) after 24 h. Each dot on the graph corresponds to the viability of each technical repeat from 3 independent experiments. Ctrl – cells untreated; MMAE – cells treated with MMAE; Crizotinib (hydrochloride) + MMAE – cells pre-treated with compound and then treated with MMAE. Results were normalized as 100% cell viability of control cells. Statistical analyses were performed using analysis of variance with Tukey HSD test, $p < 0.05$, $^{**}p < 0.005$, $^{***}p < 0.001$.

Supplementary Fig. 38

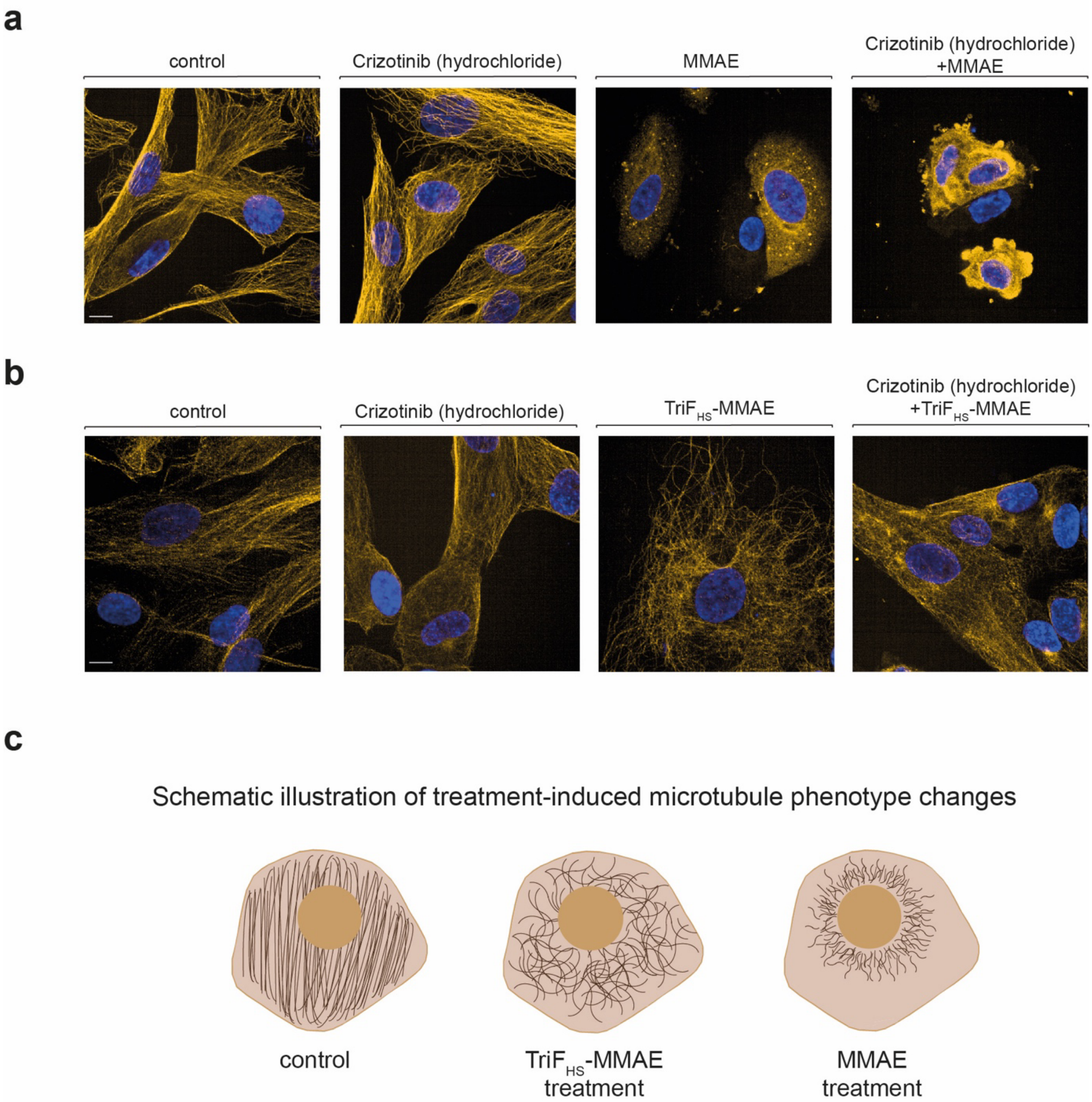

Supplementary Figure 38. Microscopy images of hTERT-HPNE cells treated with Crizotinib (hydrochloride), TriF$_{HS}$-MMAE, MMAE, or their combination. a. hTERT-HPNE cells were treated with Crizotinib (hydrochloride), MMAE or the combination for 24 h at 37°C. **b**. hTERT-HPNE cells were treated with Crizotinib (hydrochloride), TriF$_{HS}$-MMAE or the combination for 24 h at 37°C and subsequently fixed. β-tubulin (Pheno 594) was detected by immunolabeling, and nuclei were stained using NucBlue Live. Scale bar represents 10 μm. **c.** Schematic illustration of microtubule phenotype changes induced by MMAE or TriF$_{HS}$-MMAE compared to wild-type phenotype (control).

Supplementary Fig. 39

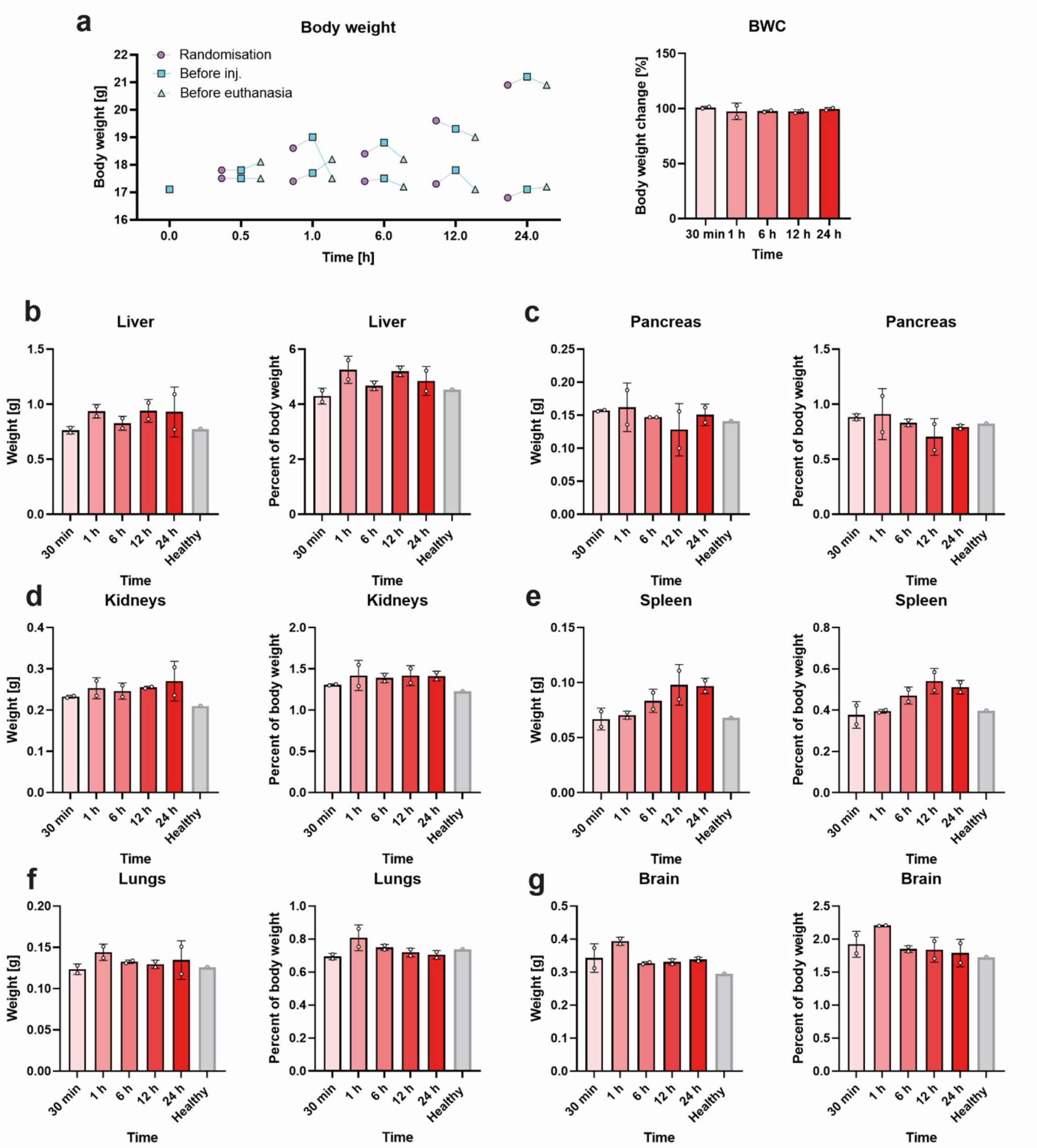

Supplementary Figure 39. Body weight and organ weights following TriF$_{HS}$–MMAE administration. a). Body weight, body weight change and b.-g). absolute and relative ((organ weight [g] x 100%)/body weight [g]) organ weights (liver, pancreas, kidneys, spleen, lungs, and brain) were recorded in mice used in the pilot biodistribution study at the indicated time points following intravenous administration of TriF$_{HS}$–MMAE. Data are shown as mean with SD with individual values.

Supplementary Fig. 40

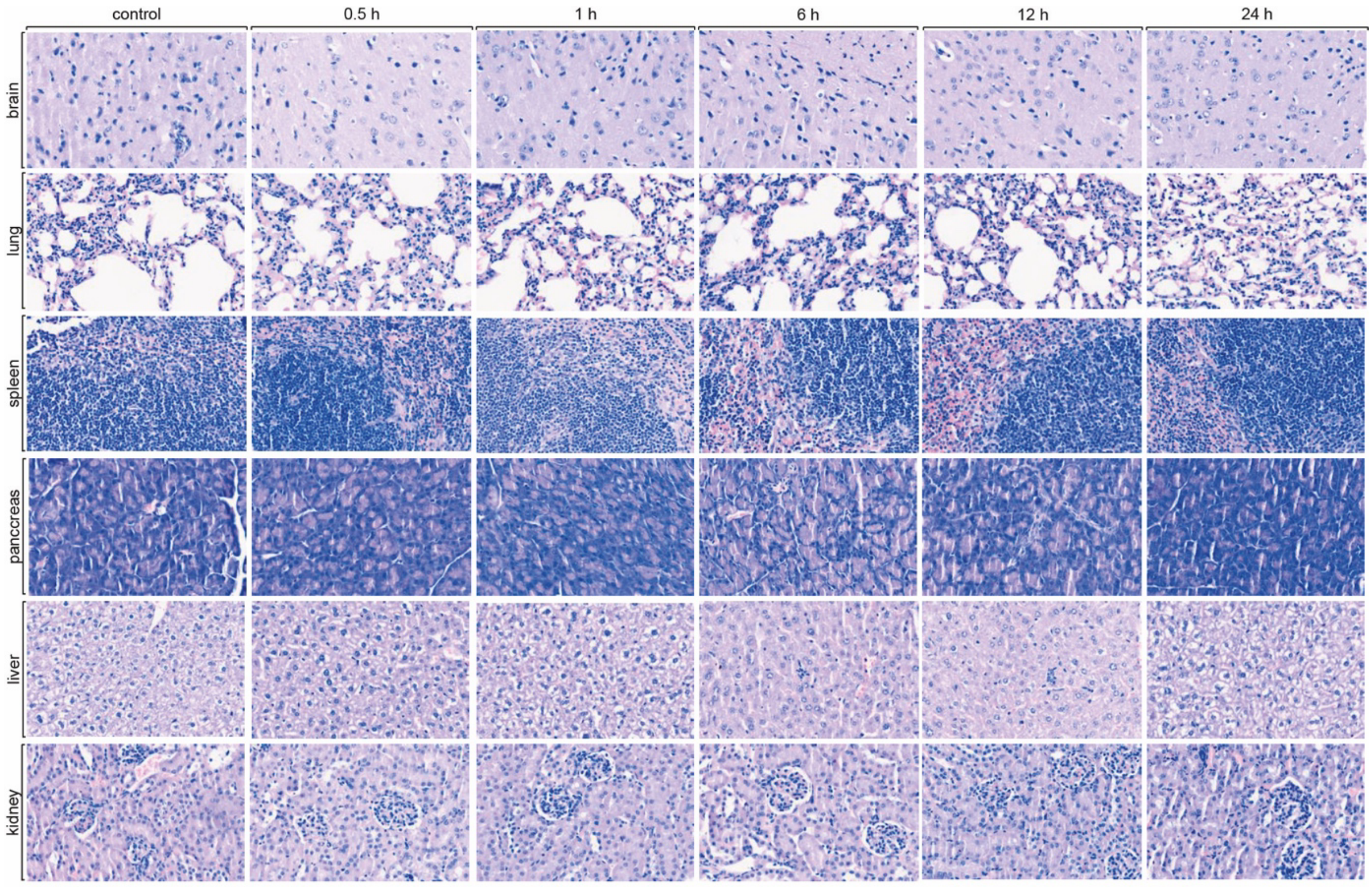

Supplementary Figure 40. Microscopy images of mouse tissues over time following TriF$_{HS}$-MMAE administration. Representative microscopic images illustrating the histology of various mouse tissues before and from 0.5 to 24 hours after TriF$_{HS}$-MMAE administration. Hematoxylin+eozin (HE), x400 magnification. Brain and Pancreas: No significant microscopic changes were observed. Lung, Spleen and Kidney: Only slight hyperemia was noted starting at 6 hours after TriFHS-MMAE administration. Liver: Cellular edema, eosinophilic changes in the cytoplasm, dilatation of sinusoids, and foci of spotty necrosis were observed, with the most intense effects occurring during the 6-12 hours following TriF$_{HS}$-MMAE administration. By 24 hours, a resolution of these changes was noted.

Supplementary Fig. 41

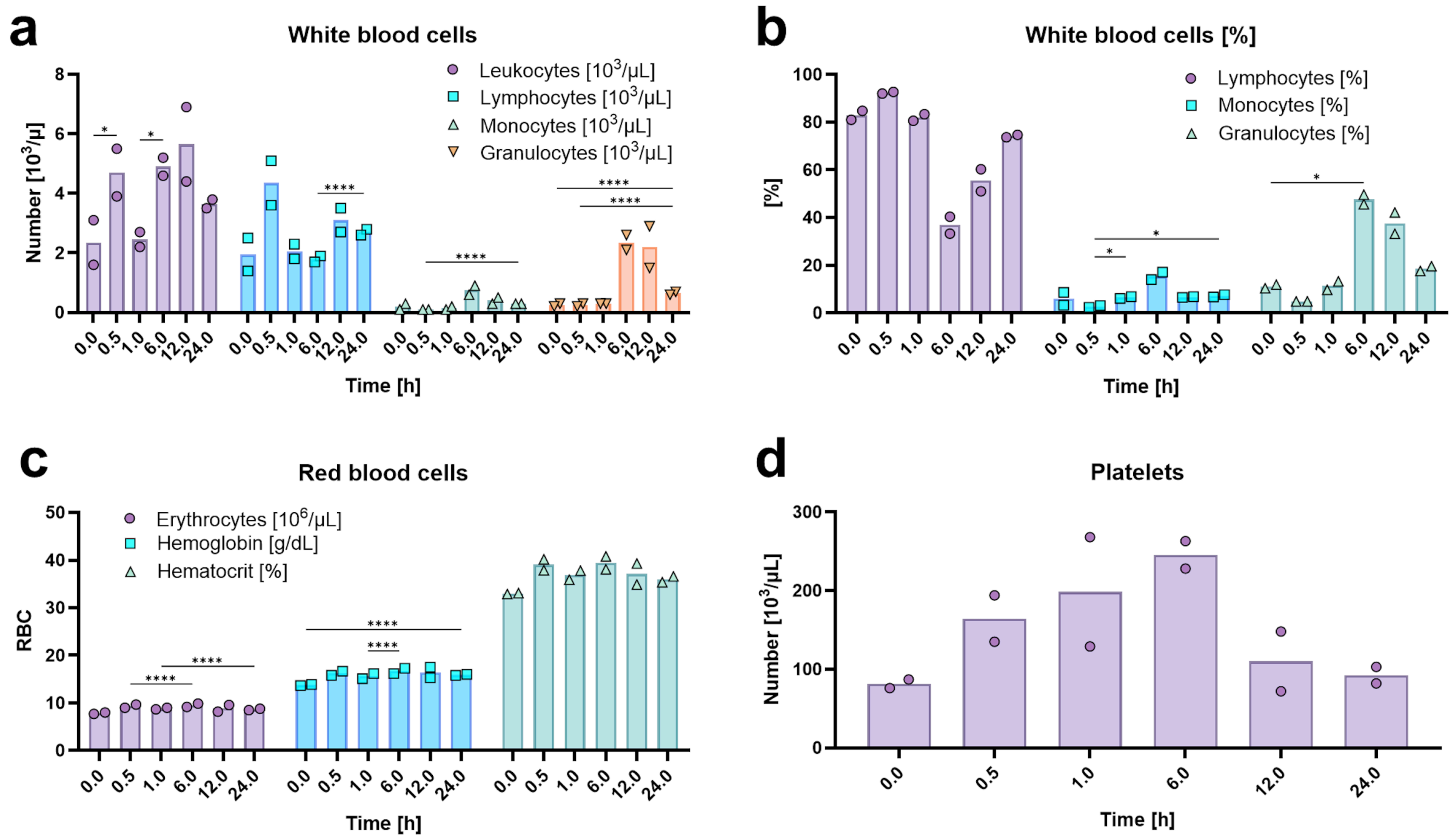

Supplementary Figure 41. Hematological analysis following TriF$_{HS}$–MMAE administration. Whole blood was collected at the indicated time points after intravenous administration of TriF$_{HS}$–MMAE and subjected to routine hematological analysis. 0.0 = healthy mice. Data are shown as mean with individual values. N=2, Statistical analysis: Tukey's multiple comparisons test; *p<0.05.

Supplementary Fig. 42

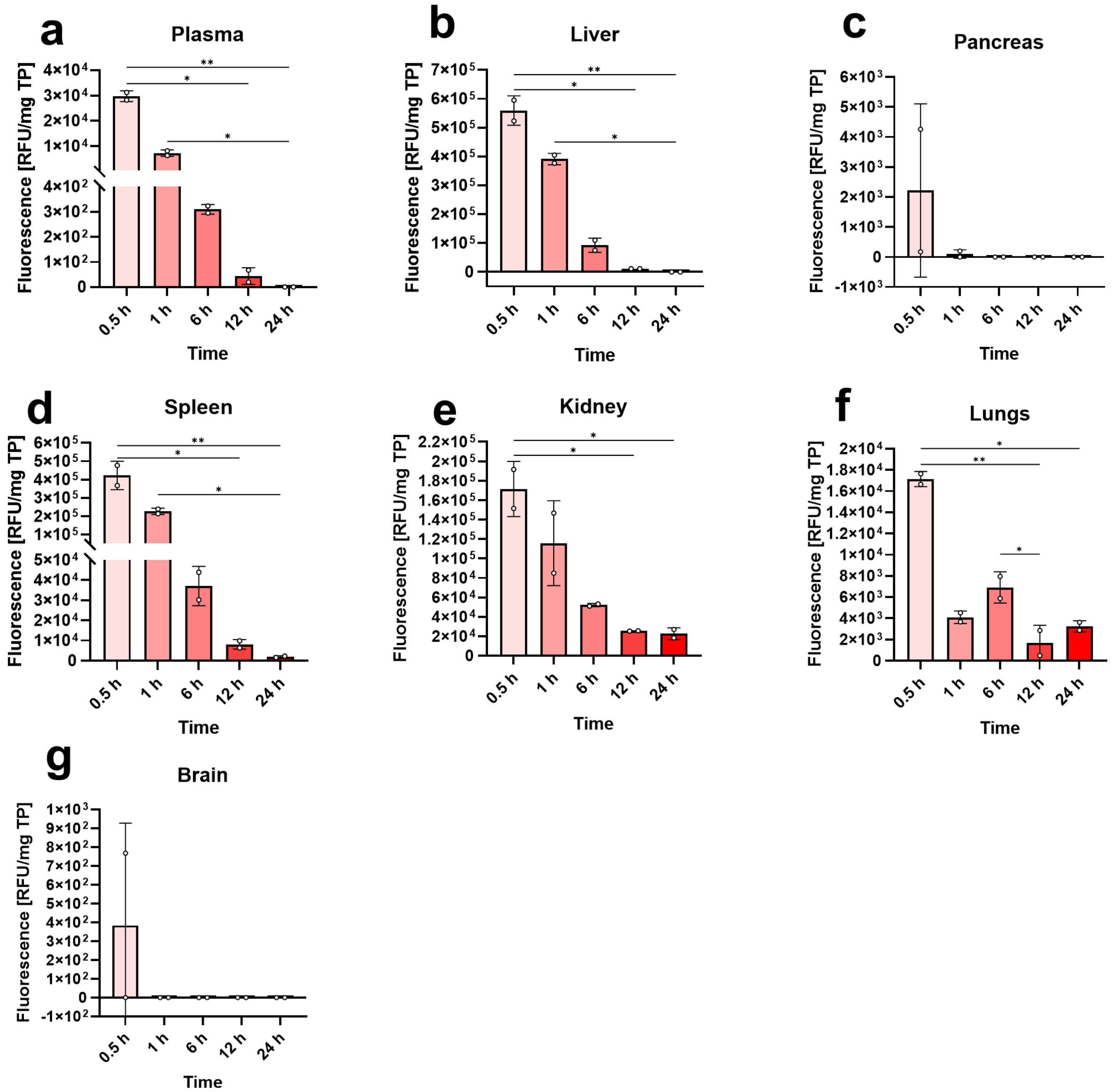

Supplementary Figure 42. Fluorescence-based biodistribution of TriF$_{HS}$–MMAE following intravenous administration. Female BALB/c mice received a single intravenous injection of the TriF$_{HS}$–MMAE conjugate (0.38 mg/kg MMAE; 23.86 mg/kg total conjugate). Plasma and indicated organs were collected at 0.5 h, 1 h, 6 h, 12 h, and 24 h post-injection (n = 2 mice per time point). Fluorescence was quantified in plasma and tissue homogenates using a plate reader and normalized to total protein content (RFU/mg TP). Background autofluorescence was defined using plasma and tissues from an untreated control mouse. For visualization only, negative background-

subtracted values were plotted as 0. Data are shown as mean with SD with individual values. N=2. Statistical analysis: Dunn's test; *p<0.05

Supplementary Fig. 43

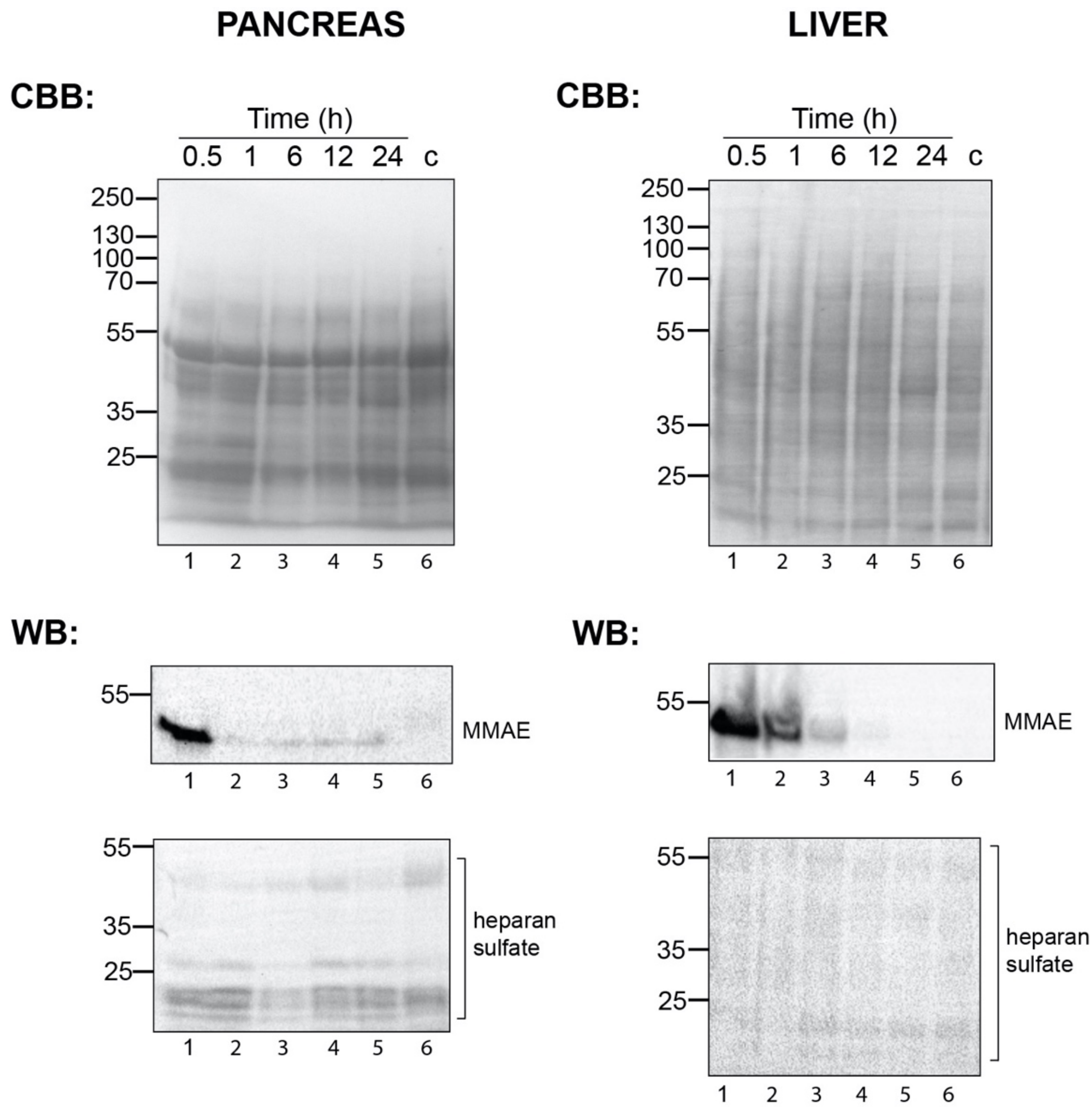

Supplementary Figure 43. Western-blot analysis of tissues (pancreas and liver) from *in vivo* experiment of mice treated with $TriF_{HS}$-MMAE. Each line represents mice euthanized after 30 min, 1 h, 6 h, 12 h, 24 h or mouse untreated (c).

Supplementary Fig. 44

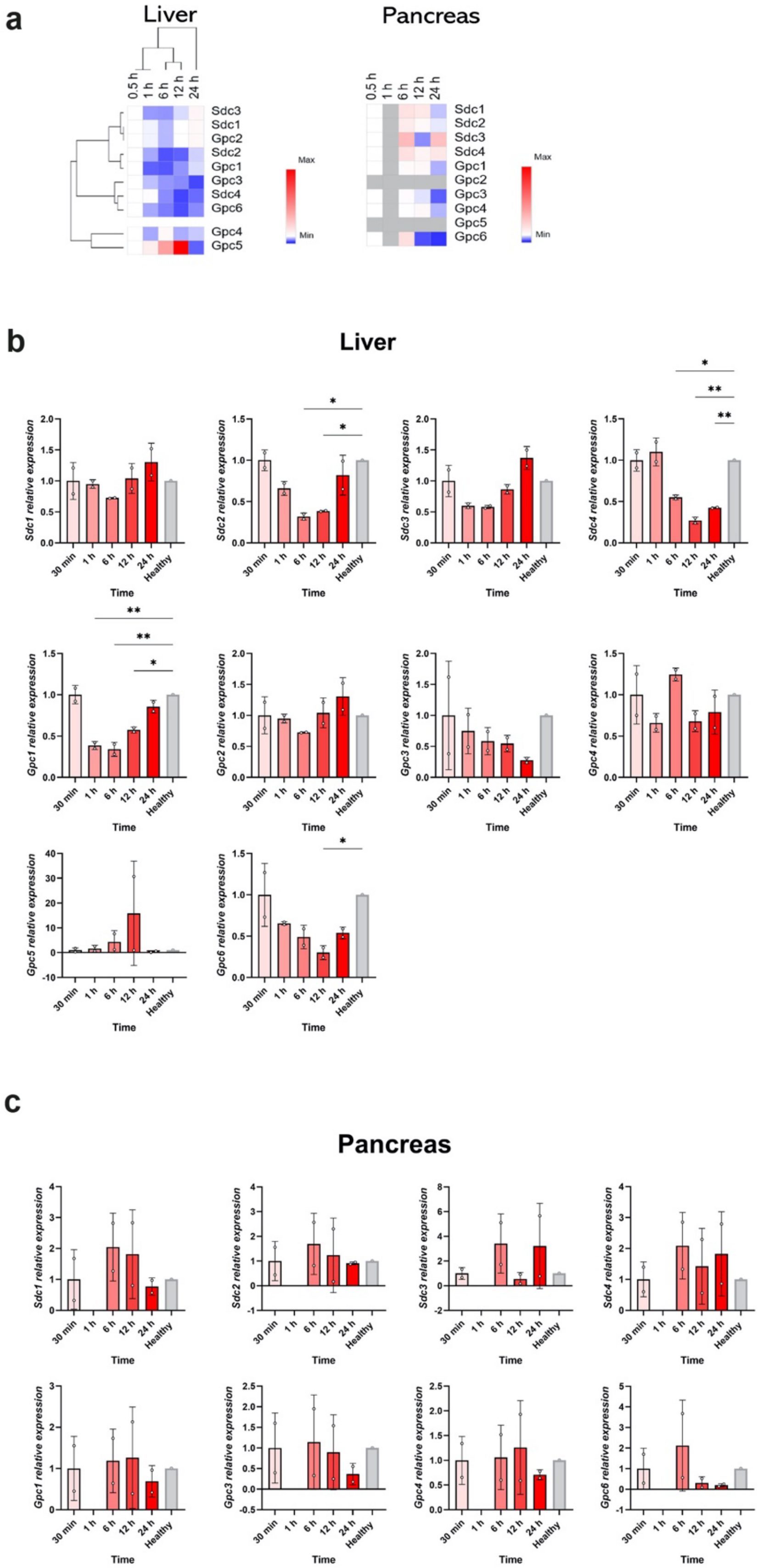

Supplementary Figure 44. The impact of TriF$_{HS}$-MMAE on the expression of proteoglycans in selected organs. a). Heat map showing time-dependent changes in the median expression of proteoglycan-encoding genes in mouse tissues. Hierarchical clustering was performed using Spearman rank correlation. Gray shading indicates nondetectable expression levels b). In liver tissue, conjugate treatment resulted in significant downregulation of Sdc2 and Sdc4 at 6 hours post-administration. In addition, Gpc1 and Gpc4 were significantly downregulated at 1 hour and 12 hours, respectively. c). In pancreatic tissue, no significant changes in the expression of proteoglycan-encoding genes were detected, and most genes exhibited low or nondetectable expression levels. Expression values were averaged and normalized to the mean expression level observed in samples collected 30 minutes after conjugate administration. Data are presented as mean ± standard deviation (SD). Statistical significance was assessed using two-way ANOVA followed by Fisher's LSD test, with $P < 0.05$ considered significant (* < 0.05; ** < 0.01).

Supplementary Fig. 45

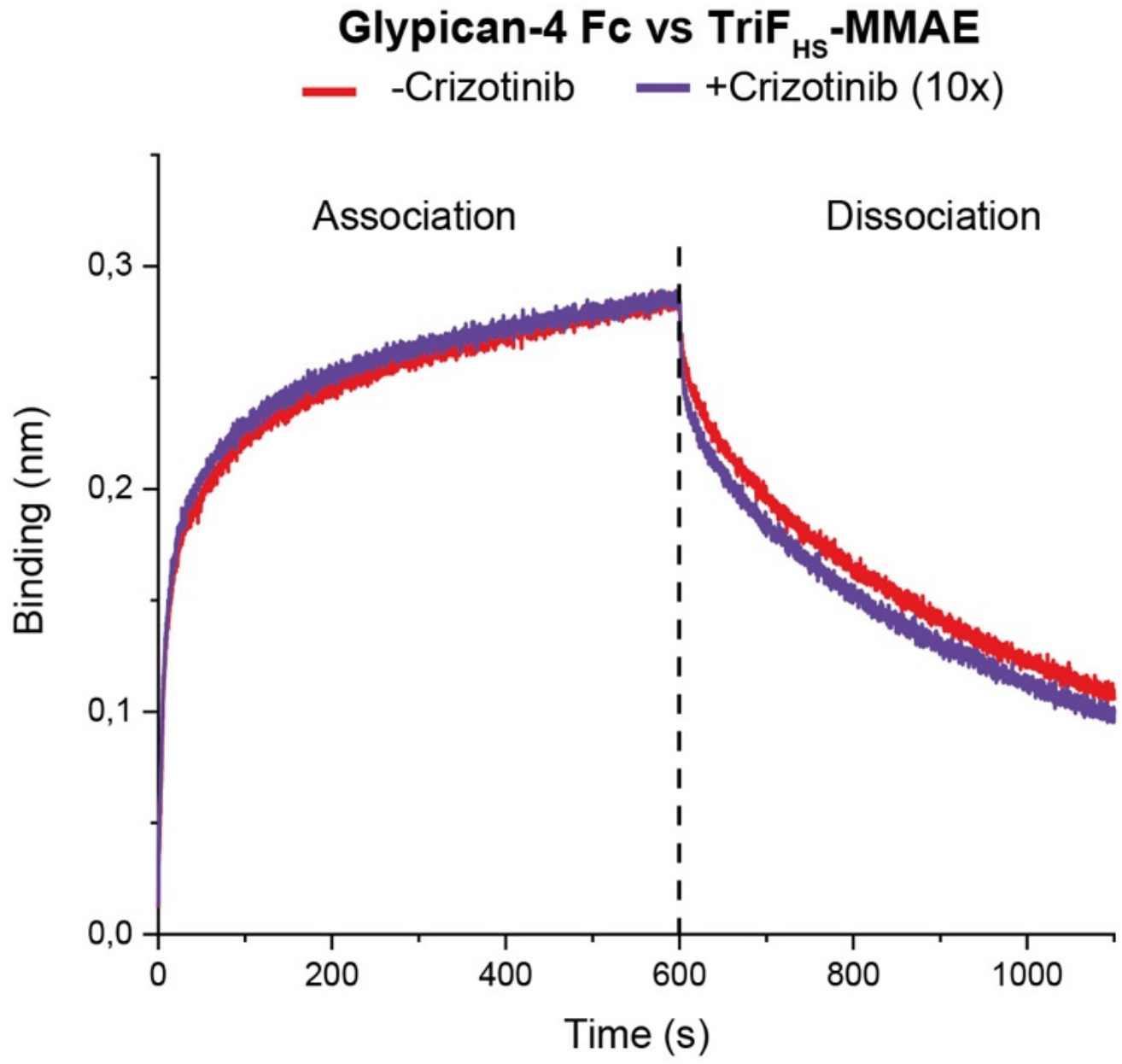

Supplementary Figure 45. Analysis of TriF$_{HS}$-MMAE binding to sensor-immobilized GPC4-Fc Chimera Protein in the presence (purple) or absence (red) of Crizotinib (hydrochloride) using biolayer interferometry (BLI). For +Crizotinib (hydrochloride) binding, sensor was incubated in solution with 10x molar excess of Crizotinib (hydrochloride) (4 μM) compared to TriF$_{HS}$-MMAE (400 nM). Concentration of TriF$_{HS}$-MMAE solution used for binding was equal for both sensors.

Supplementary Fig. 46

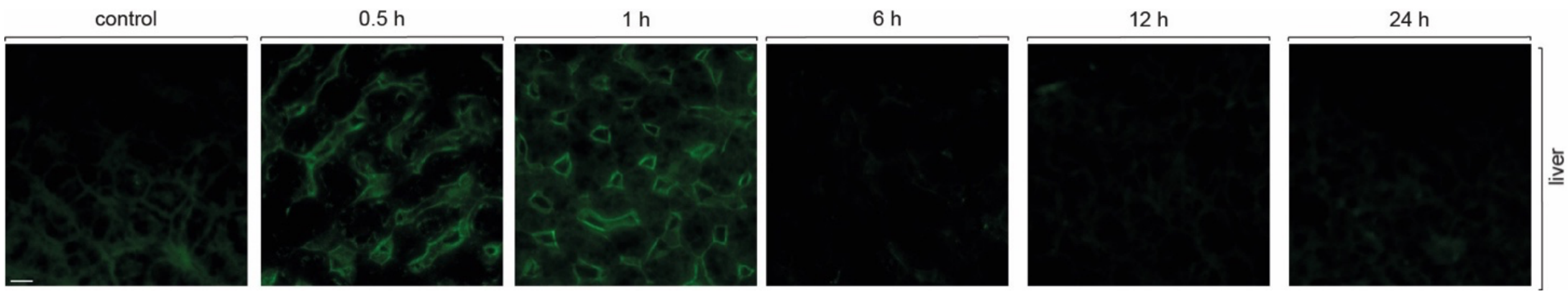

Supplementary Figure 46. Microscopy images of mouse liver over time following TriF$_{HS}$-MMAE administration. Representative images showing changes in fluorescence intensity in mouse liver at the indicated time points (0.5-24 h) before (control) and after TriF$_{HS}$-MMAE injection. Scale bar represents 10 μm.

Supplementary Fig. 47

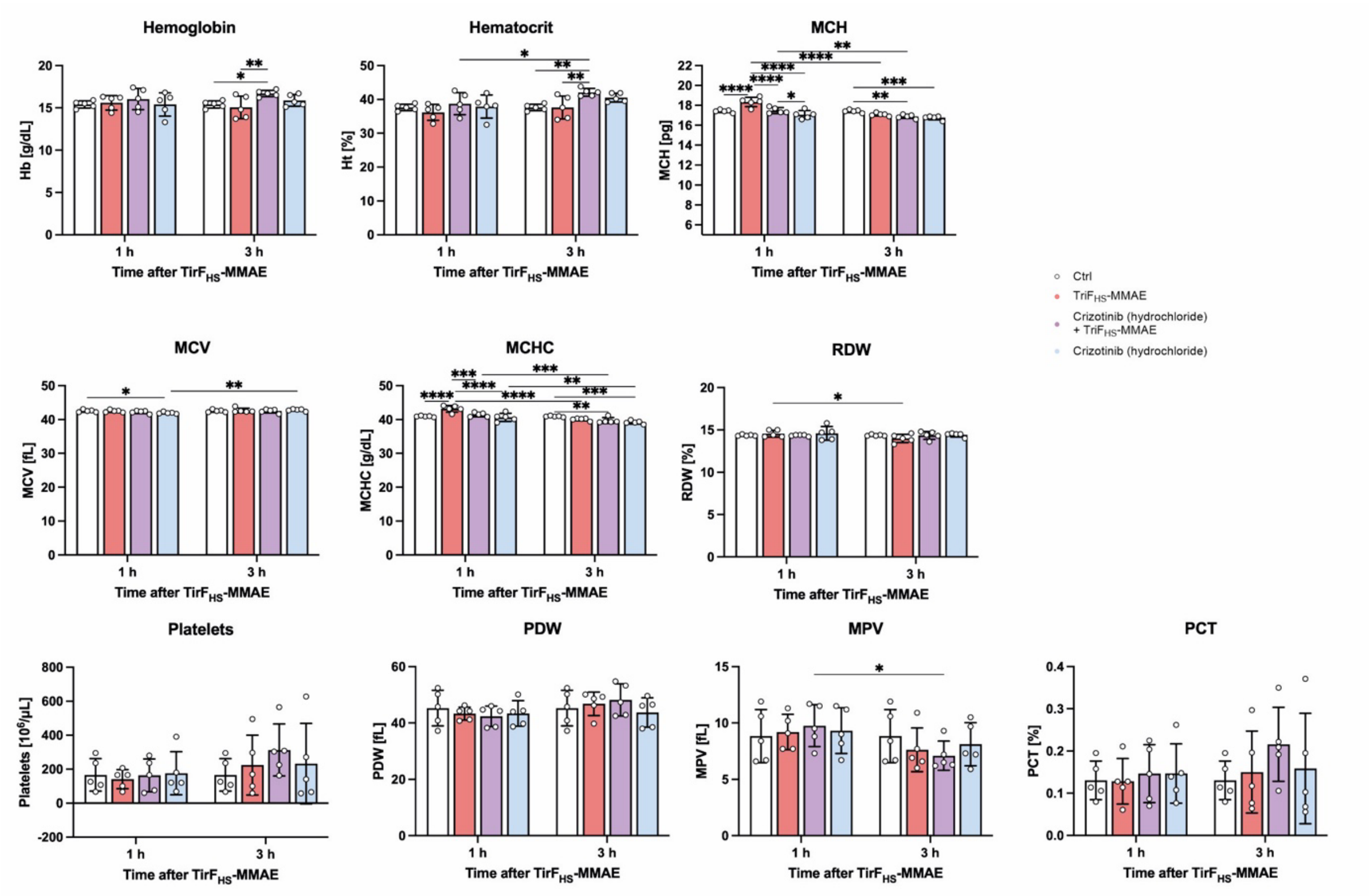

Supplementary Figure 47. Hematological parameters following TriF$_{HS}$–MMAE and Crizotinib (hydrochloride) administration. Peripheral blood was collected 1 h and 3 h after intravenous administration of TriF$_{HS}$–MMAE, oral administration of Crizotinib (hydrochloride), or their combination. Erythrocyte and platelet parameters were analyzed using an automated hematology analyzer. MCH, mean corpuscular hemoglobin; MCV, mean corpuscular volume; MCHC, mean corpuscular hemoglobin concentration; RDW, red cell distribution width; PDW, platelet distribution width; MPV, mean platelet volume; PCT, plateletcrit. Data are presented as mean ± SD with individual data (n = 5 per group). Statistical analysis was performed using two-way ANOVA followed by Fisher's LSD post hoc test. *$p < 0.05$, **$p < 0.01$, ***$p < 0.001$, ****$p < 0.0001$.

Supplementary Fig. 48

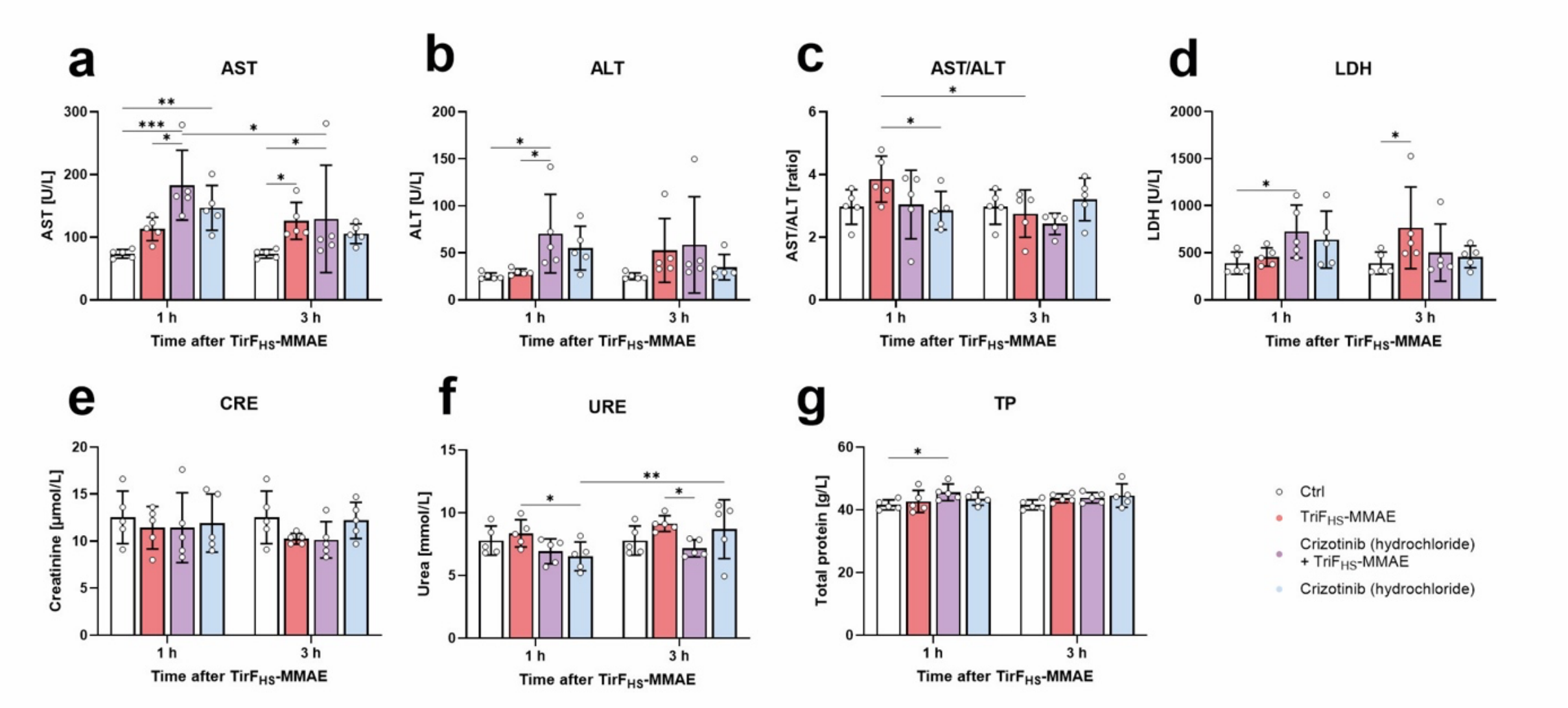

Supplementary Figure 48. Plasma biochemical parameters following TriF$_{HS}$–MMAE and Crizotinib (hydrochloride) administration. Plasma was collected 1 h and 3 h after treatment with TriF$_{HS}$–MMAE and analyzed for markers of hepatic and renal function. a). AST, aspartate aminotransferase; b). ALT, alanine aminotransferase; c). AST/ALT, ratio of aspartate to alanine aminotransferase; d). LDH, lactate dehydrogenase; e). CRE, creatinine; f). UREA, urea; g). TP, total protein. Data are presented as mean ± SD with individual data (n = 5 per group). Statistical analysis was performed using two-way ANOVA followed by Fisher's LSD post hoc test. *p < 0.05, **p < 0.01, ***p < 0.001, ****p < 0.0001

Supplementary Table 1. Table of 118 compounds that act as downregulators of internalization of TriF$_{HS}$ to hTERT-HPNE cells. Table consist of information about name of downregulator, its target and normalized value of the change in fluorescence intensity, which corresponds to changes in endocytosis efficiency.

Name of Compound	Target	Change	Name of Compound	Target	Change
Tivantinib	Apoptosis; c-Met/HGFR	0,38946382	SU1498	VEGFR	0,503597371
CID44216842	Ras	0,430061984	MT-802	Btk; PROTACs	0,396274724
ML141	Apoptosis; Ras	0,404674609	Polyphyllin I	Akt; Apoptosis; Autophagy; JNK; mTOR; PDK-1	0,48182879
MitoPQ	ROS Kinase	0,541261154	Cucurbitacin I	JAK; STAT	0,470558306
Elzovantinib	c-Fms; c-Met/HGFR; Src	0,538409506	Entrectinib	Anaplastic lymphoma kinase (ALK); Autophagy; ROS Kinase; Trk Receptor	0,344564677
GSK1059615	Apoptosis; mTOR; PI3K	0,544549679	SEL120-34A (monohydrochloride)	CDK	0,483386855
Bromosporine	Apoptosis; CDK; Epigenetic Reader Domain; HIV	0,370328893	IRAK inhibitor 4 (trans)	IRAK	0,518758387
SNS-314 (mesylate)	Aurora Kinase	0,267359662	LJH685	Apoptosis; Ribosomal S6 Kinase (RSK)	0,532454923
TAO Kinase inhibitor 1	MAP3K	0,434090096	UNC569	TAM Receptor	0,454495923
Zapnometinib	Bacterial; Influenza Virus; MEK	0,519755818	Skepinone-L	Autophagy; p38 MAPK	0,547984846
Gandotinib	FGFR; FLT3; JAK; VEGFR	0,412845425	FIIN-2	FGFR	0,460787462
FLT3-IN-6	FLT3	0,426709838	Cilengitide	Apoptosis; Autophagy; Integrin; PD-1/PD-L1; STAT	0,530382692
SAR405	Autophagy; PI3K	0,512683393	Lapatinib (ditosylate monohydrate)	Autophagy; EGFR; Ferroptosis	0,432111462
Syk-IN-4	Syk	0,363314862	Gallein	PI3K	0,496733923
Merestinib	c-Met/HGFR; Discoidin Domain Receptor; FLT3; ROS Kinase	0,471378932	JWG-071	ERK	0,448587923
ML-098	Ras	0,487683143	FLT3-IN-2	FLT3	0,453466
GW284543	MEK	0,442272635	WF-47-JS03	RET	0,523784
Fangchinoline	Apoptosis; Autophagy; FAK; HIV	0,549502705	CZ415	mTOR	0,449334692
GSK-3β inhibitor 3	Apoptosis; GSK-3	0,437399528	H-89 (dihydrochloride)	Autophagy; PKA	0,533356077

IC261	Apoptosis; Casein Kinase	0,486229049	Cu(II)GTSM	Amyloid-β; GSK-3	0,519911846
CDK-IN-2	CDK	0,537928549	hVEGF-IN-1	VEGFR	0,433161154
WZ4003	AMPK	0,474316782	Factor D inhibitor 6	Complement System	0,52541862
ASK1-IN-2	Apoptosis; MAP3K	0,544401994	MPT0B392	Apoptosis; Caspase; JNK; Microtubule/Tubulin	0,39273185
SCH900776	Checkpoint Kinase (Chk)	0,400166821	RIP2 Kinase Inhibitor 3	RIP kinase	0,54357746
Galunisertib	TGF-β Receptor	0,53841898	Src Inhibitor 1	Src	0,48479938
BO-264	Apoptosis; FGFR	0,530861067	Adagrasib	Ras	0,35204869
Lapatinib	Autophagy; EGFR; Ferroptosis	0,283003747	RAS inhibitor Abd-7	Ras	0,41737222
Mevociclib	CDK	0,439405995	Mitoxantrone (dihydrochloride)	Apoptosis; Endogenous Metabolite; Orthopoxvirus; PKC; Topoisomerase	0,54295094
Stattic	Apoptosis; STAT	0,492344546	Crizotinib (hydrochloride)	Anaplastic lymphoma kinase (ALK); Autophagy; c-Met/HGFR; ROS Kinase	0,35162635
Aspirin	Apoptosis; Autophagy; Caspase; COX; Mitophagy; NF-κB; p38 MAPK; Virus Protease	0,542750125	MLS-573151	Ras	0,34098516
CCT020312	Autophagy; PERK	0,362194505	CDK9-IN-7	Apoptosis; CDK	0,52365712
FMF-04-159-2	CDK	0,454789759	Aldometanib	AMPK	0,43710186
Epertinib (hydrochloride)	EGFR	0,416646711	7rh	Discoidin Domain Receptor	0,5300157
PI3K-IN-36	PI3K	0,468270691	STAT3-IN-3	Apoptosis; STAT	0,53765365
HMN-176	Polo-like Kinase (PLK)	0,526318568	GW406108X	Autophagy; Kinesin; ULK	0,51252103
AT7867 (dihydrochloride)	Akt; PKA; Ribosomal S6 Kinase (RSK)	0,475865279	Dihydrodiol-Ibrutinib	Btk	0,48482132
Coenzyme Q0	Akt; Apoptosis; Autophagy; Bcl-2 Family; Caspase; COX; EGFR; Interleukin Related; MMP; mTOR; NF-κB; NO Synthase; PARP; Reactive	0,474115487	DIF-3	CDK; GSK-3	0,32615332

	Oxygen Species; TNF Receptor				
SC-43	Apoptosis; Phosphatase ; STAT	0,426038448	RLX-33	ERK	0,28554177
TP-5801	Others	0,084045859	Platycodin D	AMPK; Wnt	0,51149587
O-Demethyl Lenvatinib (hydrochloride)	Drug Metabolite	0,420390547	SH-4-54	STAT	0,4733228
GNF362	Phosphatase	0,501945	Fasudil (Hydrochloride)	Autophagy; Calcium Channel; HIV; PKA; PKC; ROCK	0,54288302
A-443654	Akt	0,547627109	Buparlisib	Apoptosis; PI3K	0,52932276
Ribociclib succinate	CDK	0,446133906	CASIN	Apoptosis; Ras	0,53344761
MAX-40279	FGFR; FLT3	0,413431797	Bentamapimod	JNK	0,44568224
Bohemine	CDK; ERK	0,462280938	Skp2 Inhibitor C1	E1/E2/E3 Enzyme	0,39904528
Tomatidine	Autophagy; Endogenous Metabolite; JNK; NF-κB	0,255313281	AAL993	VEGFR	0,50553037
Borussertib	Akt	0,386579063	Almonertinib (mesylate)	EGFR	0,46972331
Niclosamide	Antibiotic; Parasite; STAT	0,336905703	ARS-1323-alkyne	Ras	0,32433783
KPLH1130	PDHK	0,547441797	OSU-T315	Apoptosis; Autophagy; Integrin	0,42432736
Rigosertib (sodium)	Apoptosis; PI3K; Polo-like Kinase (PLK)	0,476062421	Cobimetinib (racemate)	MEK	0,52738256
Ki20227	c-Fms; c-Kit; PDGFR; VEGFR	0,4755022	ASP5878	FGFR	0,37811166
Selatinib	EGFR	0,535050613	IACS-13909	Phosphatase; SHP2	0,51343636
BGT226 (maleate)	Apoptosis; Autophagy; mTOR; PI3K	0,492384555	WAY-181187	5-HT Receptor	0,53527383
JI-101	Ephrin Receptor; PDGFR; VEGFR	0,399455131	4SC-203	FLT3; VEGFR	0,54003549
Osimertinib (mesylate)	EGFR	0,488623494	Fingolimod (hydrochloride)	LPL Receptor; PAK	0,19197573
Paltimatrectinib	Trk Receptor; Tyrosinase	0,328137574	Necrolr2	CDK; Mixed Lineage Kinase; RIP kinase	0,52337576
IMD-0560	IKK	0,486444699	Tirbanibulin	Microtubule/Tubulin ; Src	0,51321827
M2N12	CDK	0,263974979	(1E)-CFI-400437 (dihydrochloride)	Polo-like Kinase (PLK)	0,38006189
BRD7389	Ribosomal S6 Kinase (RSK)	0,433980916	Bakuchiol	Autophagy; p38 MAPK	0,28800717

Supplementary Table 2. Table of 131 compounds that are upregulators of internalization of TriF$_{HS}$ to hTERT-HPNE cells. Table consist of information about name of upregulator, its target and normalized value of the change in fluorescence intensity, which corresponds to changes in endocytosis efficiency.

Name of Compound	Target	Change	Name of Compound	Target	Change
APS6-45	Ras	1,564811447	SU14813 (maleate)	c-Kit; PDGFR; VEGFR	1,623655914
SU5408	VEGFR	1,832165221	3BrB-PP1	Others	1,595278873
HKI-357	EGFR	1,598185702	ESI-08	Ras	1,596526159
Esculentoside H	JNK; NF-κB	1,551636604	PF-06263276	JAK	1,768651635
THZ1 (Hydrochloride)	CDK	1,888341747	Atranorin	Endogenous Metabolite; Ras	1,595278873
IRE1α kinase-IN-2	**IRE1**	1,57180788	hSMG-1 inhibitor 11e	CDK; mTOR; PI3K	1,752436916
Fimepinostat	Apoptosis; HDAC; PI3K	1,602840612	Quercetin (dihydrate)	Apoptosis; PI3K	1,55162386
MID-1	Insulin Receptor	1,668255993	SU6656	Akt; FAK; Src	1,845983371
HPK1-IN-4	MAP4K	1,650575763	CP-673451	PDGFR	1,898493259
(Rac)-BL-918	Autophagy; ULK	1,590379407	GW779439X	Apoptosis; Aurora Kinase; Bacterial	2,16057483
PD 169316	Autophagy; Enterovirus ; p38 MAPK	1,693782167	RWJ-67657	p38 MAPK	1,9156448
SJF620	Btk; PROTACs	1,682017396	Sunitinib	Apoptosis; Autophagy; IRE1; Mitophagy; PDGFR; VEGFR	2,47363109
EOC317	FGFR; VEGFR	1,636068245	GSK180736A	ROCK	1,86860526
Cerdulatinib (hydrochloride)	JAK; Syk	1,584549501	Lirafugratinib (hydrochloride)	EGFR	1,6626045
Decursin	Apoptosis; CXCR; PKC	1,62214426	CC-115	DNA-PK; mTOR	1,57607413
GSK269962A	ROCK	1,888092375	Pumecitinib	JAK	2,00485772
Infigratinib	Apoptosis; FGFR	1,668093411	SCH772984	ERK	2,03081058
TCS 21311	GSK-3; JAK; PKC	1,597081087	Autophinib	Autophagy; PI3K	1,62854139
1-Naphthyl PP1	Src	1,665308614	Flavopiridol	Apoptosis; Autophagy; CDK; HIV	1,5884989
ZD-4190	EGFR; VEGFR	1,681945744	Narazaciclib	AMPK; CDK; PDGFR	1,65287219
GSK461364	Polo-like Kinase (PLK)	1,81937193	YS-49	Adrenergic Receptor; Akt; Angiotensin Receptor; PI3K	1,74532922

Tanzisertib	JNK	2,28147856	VEGFR-IN-1	c-Kit; EGFR; VEGFR	1,59188055
Y-33075 (dihydrochloride)	ROCK	1,584778276	HyT36	Others	1,60324255
GDC-0425	Checkpoint Kinase (Chk)	1,635917566	BSJ-4-116	CDK; PROTACs	1,77128207
Ilorasertib (hydrochloride)	Aurora Kinase; PDGFR; VEGFR	1,580961727	Tauroursodeoxycholate (sodium)	Apoptosis; Caspase; Endogenous Metabolite; ERK	1,51943544
XMD17-109	ERK	1,688910697	LY294002	Apoptosis; Autophagy; Casein Kinase; DNA-PK; PI3K	1,72586458
RGB-286638 (free base)	CDK; GSK-3; JAK; MEK	1,605406451	Prexasertib	Apoptosis; Checkpoint Kinase (Chk)	1,91402275
Cirtuvivint	CDK	1,662983425	BLU-945	EGFR	1,61683876
MSC2360844 (hemifumarate)	PI3K	1,61878453	CCT128930	Akt; Apoptosis; Autophagy	1,66909272
DLK-IN-1	MAP3K	1,643646409	Regorafenib (Hydrochloride)	Autophagy; PDGFR; Raf; RET; VEGFR	1,73073074
Cromolyn (sodium)	Calcium Channel; GSK-3	1,658175248	SR-3029	Casein Kinase	1,71288815
MRT67307	Autophagy; IKK; ULK	1,671634463	CPS2	CDK	1,63340755
Nifuroxazide	Antibiotic; Bacterial; STAT	1,562614824	TBK1/IKKε-IN-5	IKK	1,80534518
Momelotinib	Apoptosis; Autophagy; JAK	1,565306667	AZD3229	c-Kit	1,59051608
STK16-IN-1	Others	1,597608782	SL 0101-1	Ribosomal S6 Kinase (RSK)	1,62854139
Pazopanib (Hydrochloride)	Autophagy; c-Fms; c-Kit; FGFR; PDGFR; VEGFR	1,697206971	GNE0877	LRRK2	1,69180146
Cerdulatinib	JAK; Syk	1,662213013	NSC-370284	STAT; TET Protein	1,74532922
Hispidulin	Pim	1,795459238	Senexin A	CDK	2,16544099
Hck-IN-1	HIV; Src	1,602992468	Artesunate	Ferroptosis; Parasite; STAT; Virus Protease	1,58040112
Enitociclib	CDK	1,753735672	Fraxinellone	HIF/HIF Prolyl-Hydroxylase; PD-1/PD-L1; STAT	1,6869353
KIRA6	IRE1	1,765848966	mTOR inhibitor-8	Autophagy; mTOR	1,70802199
IRAK4-IN-1	IRAK	1,748351986	3-IN-PP1	PKD	1,79561287
ALK2-IN-4	TGF-β Receptor	1,580111803	Methyl 2,5-dihydroxycinnamate	EGFR	1,63665165

Desmethyl Erlotinib	Drug Metabolite	1,998693379	Indirubin Derivative E804	IGF-1R	1,68739206
Famitinib	Apoptosis; PDGFR; VEGFR	1,62244999	PHPS1 (sodium)	Phosphatase ; SHP2	2,04845815
AR-13324 M1 metabolite	Drug Metabolite	1,560254286	Regorafenib	Autophagy; c-Kit; FGFR; PDGFR; Raf; RET; VEGFR	1,60764712
PKR-IN-C16	Apoptosis	1,761854153	SC-236	Apoptosis; COX; PPAR	1,56260193
Agerafenib	Raf	1,646041463	MTX-211	EGFR; PI3K	1,67443826
2,6-Dichloro-N-(2-(cyclopropanecarboxamido)pyridin-4-yl)benzamide	JAK	1,670705277	Torkinib	Apoptosis; Autophagy; Mitophagy; mTOR	1,71948344
Tarloxotinib (bromide)	EGFR	1,73397332	PLX7922	Raf	1,63094635
BMS-986176	AAK1	2,331266542	Prexasertib (dimesylate)	Apoptosis; Checkpoint Kinase (Chk)	1,73812283
RAF265	Apoptosis; Autophagy; Raf; VEGFR	1,856220048	AD57	Raf; RET; Ribosomal S6 Kinase (RSK); Src	2,25070597
BMS-986142	Btk	1,747913736	HER2/neu (654-662) GP2	EGFR	1,74278267
PCI-33380	Btk	3,970873967	TC ASK 10	Apoptosis; MAP3K	1,72569657
XMD-17-51 (TFA)	Others	1,890534918	IRE1α kinase-IN-1	IRE1	1,64492589
TCS7010	Apoptosis; Aurora Kinase	1,561326626	ML251	Parasite	1,69773749
Gartisertib	ATM/ATR	1,579636183	PKC-iota inhibitor 1	PKC	2,21653376
HSD1590	ROCK	1,605025164	BMS 777607	c-Met/HGFR; TAM Receptor	1,76763519
MK-0941	Glucokinase	1,624843589	Pexidartinib (hydrochloride)	Apoptosis; c-Fms; c-Kit	1,60454055
p38-α MAPK-IN-1	Autophagy; p38 MAPK	1,591902834	AG-024322	Apoptosis; COX	2,19012796
Peficitinib	JAK	1,71417004	Astragaloside IV	ERK; JNK; MMP	1,61230697
AD80	Raf; RET; Ribosomal S6 Kinase (RSK); Src	1,705263158	LY2109761	Autophagy; TGF-β Receptor	2,02703332
FRAX597	PAK	2,192712551	NU6140	Aurora Kinase; CDK	1,67288497
Tuvusertib	ATM/ATR	2,234817814	CHIR-124	Apoptosis; Checkpoint Kinase (Chk); FLT3; PDGFR	2,25847238
Sovesudil (hydrochloride)	ROCK	1,565991903	PF-AKT400	Akt	1,56260193
ZX-29	Anaplastic lymphoma kinase (ALK); Apoptosis; Autophagy	1,833198381	-	-	-

Supplementary Table 3. Table of statistically significant 39 inhibitors of internalization of $TriF_{HS}$ to hTERT-HPNE cells. Table consist of information about name of inhibitor, its target and UniProt code. Some targets have been changed from proteins to kinases that are in signaling pathways above the protein in question.

Inhibitor	Target	UniProt code
CID44216842	Cdc42	P60953
ML141	Cdc42	P60953
Lapatinib	EGFR	Q504U8
	HER2	P04626
Epertinib (hydrochloride)	EGFR	Q504U8
Selatinib	EGFR	Q504U8
Entrectinib	TRK A	P04629
	TRK B	Q16620
	TRK C	Q16288
	ALK	Q9UM73
IRAK inhibitor 4 (trans)	IRAK 4	Q1RMT8
Lapatinib (ditosylate monohydrate)	EGFR	Q504U8
	HER2	P04626
Mevociclib	CDK 7	P50613
MPT0B392	JNK 1	P45983
	JNK 2	P45984
	JNK 3	P53779
RIP2 Kinase Inhibitor 3	RIPK2	O43353
Adagrasib	Raf1	P04049
RAS inhibitor Abd-7	PI3K	P42336
Mitoxantrone (dihydrochloride)	JNK 1	P45983
	JNK 2	P45984
	JNK 3	P53779
Crizotinib (hydrochloride)	ALK	Q9UM73
MLS-573151	Raf1	P04049
CDK9-IN-7	Cdk 9	P50750
Aldometanib	AMPK α1	Q13131
	AMPK α2	P54646
7rh	Ddr1	Q08345
STAT3-IN-3	STAT 3	P40763
RLX-33	ERK	P27361
Platycodin D	AMPK α1	Q13131
	AMPK α2	P54646
SH-4-54	STAT 3	P40763
Buparlisib	PIK3R4	Q99570
CASIN	CK1a	Q8N752
Bentamapimod	JNK 1	P45983
	JNK 2	P45984
	JNK 3	P53779
Skp2 Inhibitor C1	-	
Almonertinib (mesylate)	EGFR	Q504U8

ARS-1323-alkyne	Raf1	P04049
Cobimetinib (racemate)	MAP2K	A4QPA9
ASP5878	FGFR1	P11362
	FGFR2	P21802
	FGFR3	P22607
	FGFR4	P22455
IACS-13909	SRC	P12931
WAY-181187	FYN	P06241
	ERK 1	P27361
	ERK 2	P28482
4SC-203	FLT3	P36888
Fingolimod (hydrochloride)	AKT 1	P31749
NecroIr2	RIPK3	Q9Y572
Tirbanibulin	SRC	P12931
(1E)-CFI-400437 (dihydrochloride)	PLK4	O00444
Bakuchiol	P38	A0A510GDE6

Supplementary Table 4. Table of statistically significant 32 inducers of internalization of $TriF_{HS}$ to hTERT-HPNE cells. Table consist of information about name of inducer, its target and UniProt code. Some targets have been changed from proteins to kinases that are in signaling pathways above the protein in question.

Inhibitor	Target	UniProt code
IRE1α kinase-IN-2	IRE 1	Q09499
Cerdulatinib (hydrochloride)	JAK 1	P23458
	Syk	P43405
HPK1-IN-4	MAP4K	Q5MD60
PCI-33380	Bruton's tyrosine kinase	Q06187
AR-13324 M1 metabolite	Drug metabolite	-
Y-33075 (dihydrochloride)	Rho	Q13464
AD80	Raf	P04049
	Src	P12931
Peficitinib	JAK 1	P23458
	JAK 2	O60674
	JAK 3	P52333
Cerdulatinib	JAK 1	P23458
	Syk	P43405
Tuvusertib	ATM	Q13315
	ATR	Q13535
TCS 21311	GSK3	P49840
	JAK	P23458
	Protein kinase C	B4DFV1
GDC-0425	Checkpoint Kinase (Chk)	O14757
CP-673451	PDGFR	P16234
SU14813 (maleate)	PDGFR	P16234
	KIT	P10721
XMD-17-51 (TFA)	-	-
Cirtuvivint	CDK 1	P06493
	DYRK 1	Q13627
HSD1590	ROCK 1	Q13464
	ROCK 2	O75116
Gartisertib	ATM	Q13315
	ATR	Q13535
Momelotinib	JAK	P23458
Famitinib	PDGFR	P16234
	VEGFR	P17948
MID-1	IRS1	P35568
GSK269962A	ROCK 1	Q13464
	ROCK 2	O75116

2,6-Dichloro-N-(2-(cyclopropanecarboxamido)pyridin-4-yl)benzamide	JAK	P23458
Agerafenib	Raf	P04049
IRE1α kinase-IN-2	IRE 1	Q09499
MRT67307	IKKb	O14920
	TBK1	A0A494C1M6
	ULK1	O75385
Esculentoside H	JNK 1	P45983
	JNK 2	P45984
PKR-IN-C16	Apoptosis	-
MK-0941	GCK	A0A8C8KJG0
PHPS1 (sodium)	SHP-2	
Prexasertib	Checkpoint Kinase (Chk)	O14757
CHIR-124	Checkpoint Kinase (Chk)	O14757
	PDGFR	P16234
	FLT3	E7ER61
NU6140	CDK 1	P06493
	AurA	O14965
	AurB	Q96GD4
Prexasertib (dimesylate)	Checkpoint Kinase (Chk)	O14757

Supplementary Table 5. TaqMan Probes IDs used in the measurement of mRNA quantitative Real Time PCR.

	Assay Name	Assay ID
Gene Expression Assay	*Sdc1*	Mm00448918_m1
	Sdc2	Mm04207492_m1
	Scd3	Mm01179833_m1
	Sdc4	Mm00488527_m1
	Gpc1	Mm01290371_m1
	Gpc2	Mm00549650_m1
	Gpc3	Mm01311297_m1
	Gpc4	Mm00515035_m1
	Gpc5	Mm00615599_m1
	Gpc6	Mm00516235_m1
	GAPDH	Mm99999915_g1
	SDHA	Mm01352366_m1
	Actb	Mm02619580_g1

Amino acid sequences:

1. FGF1$_{HS}$

MANYKKPKLLYSSNGGHFLRILPDGTVDGTRDRSDPHIQLQLIAESVGEVYIKSTETG
QYLAMDTDGLLYGSQTPNEESLFLERLEENGAATYISKKHAEKNWFVGLKKNGSSK
RGPRTHYGQKAILFLPLPVSSD

2. GFPp-FGF1$_{HS}$

MSSGLVPRGSHMRDHMVLHEYVNAAGITGGTKGEELFTGVVPILVELDGDVNGHE
FSVRGEGEGDATIGKLTLKFICTTGKLPVPWPTLVTTLTYGVQCFSRYPDHMKRHDF
FKSAMPEGYVQERTISFKDDGKYKTRAVVKFEGDTLVNRIELKGTDFKEDGNILGHK
LEYNFNSHDVYITADKQENGIKAEFTVRHNVEDGSVQLADHYQQNTPIGDGPVLLP
DNHYLSTQTVLSKDPNEKGSGGTANYKKPKLLYSSNGGHFLRILPDGTVDGTRDRS
DPHIQLQLIAESVGEVYIKSTETGQYLAMDTDGLLYGSQTPNEESLFLERLEENGAA
TYISKKHAEKNWFVGLKKNGSSKRGPRTHYGQKAILFLPLPVSSDSGGSGGLPETG
GHHHHHH

Supplementary Movie S1. Internalization kinetics of TriF$_{HS}$ (Alexa 488) to hTERT-HPNE cells. Nuclei were stained using NucBlue Live. Images were captured every 1 second.
